# New variochelins from soil-isolated *Variovorax* sp. H002

**DOI:** 10.3762/bjoc.20.63

**Published:** 2024-04-02

**Authors:** Jabal Rahmat Haedar, Aya Yoshimura, Toshiyuki Wakimoto

**Affiliations:** 1 Faculty of Pharmaceutical Sciences, Hokkaido University, Kita 12, Nishi 6, Sapporo 060-0812, Japan

**Keywords:** antimicrobial activity, siderophore, variochelin, *Variovorax*

## Abstract

The soil bacterial genus *Variovorax* produce distinct photoreactive siderophores that may play a crucial role in the iron cycle within the rhizosphere. This study focused on exploring the natural products of the soil-isolated *Variovorax* sp. H002, leading to the isolation of variochelins A–E (**1**–**5**), a series of lipohexapeptide siderophores. NMR and MS/MS analyses revealed that these siderophores share a common core structure – a linear hexapeptide with β-hydroxyaspartate and hydroxamate functional groups, serving in iron-binding coordination. Three new variochelins C–E (**3**–**5**) were characterized by varied fatty acyl groups at their *N*-termini; notably, **4** and **5** represent the first variochelins with *N*-terminal unsaturated fatty acyl groups. Furthermore, the variochelin biosynthetic gene cluster was identified through draft genome sequencing and gene knockout experiments. Compounds **1**–**5** exhibited antimicrobial activities against Gram-negative bacteria, including several soil-isolated plant pathogens.

## Introduction

Almost all organisms require iron as a crucial cofactor of enzymes essential for their physiological functions, encompassing primary and secondary metabolisms [1]. However, in an aerobic environment, iron predominantly exists in the form of insoluble ferric oxide, diminishing its bioavailability. To mitigate this problem, many microorganisms utilize specialized metabolites known as siderophores to efficiently sequester iron. Siderophores are abundantly produced under iron-deficient growth conditions and secreted through dedicated transporters to form the siderophore Fe(III) complex extracellularly [2–3]. Subsequently, the stable and water soluble siderophore Fe(III) complex is transported across the cell membrane into the cytoplasm, where the iron is released from the complex by reduction, generating free Fe(II) ions that are accessible for the cell [2–3].

In addition to the siderophores forming the stable Fe(III) complexes described above, certain siderophores create Fe(III) complexes with the ability to release Fe(II) ions in a light-responsive manner within the extracellular environment. The released Fe(II) ions in the ocean serve as a nutrient source for phytoplankton and other organisms [4]. The distinctive structural feature of these siderophores is the α-hydroxy carboxylic acid (i.e., β-hydroxyaspartate), relevant to a photoreactive functional group [2,4]. In 2016, using the gene encoding a hydroxylase specific to the aspartate residue as a query, Nett et al. performed genome mining of photoreactive siderophores and isolated two such siderophores, variochelins A and B, from the soil bacterium *Variovorax boronicumulans* [5].

The *Variovorax* spp. belonging to plant growth-promoting rhizobacteria (PGPR) enhance plant development across several conditions, such as increased levels of auxin plant hormone (IAA) [6] and reduced ethylene levels [7]. They also play a crucial ecological role in the degradation of environmentally harmful pollutants [8–10]. Moreover, these bacteria produce unique photoreactive siderophores other than variochelins, including vacidobactin from *V. paradoxus* S110, variobactin from *V. paradoxus* P4B, and imaqobactin from *Variovorax* sp. RKJM285, suggesting their significant role in interactions with both plants and other microbes in near-surface soil through iron photocycling [5,11–12].

In this study, we isolated three new congeners of variochelin-type siderophores, variochelins C–E (**3**–**5**), along with two known compounds, variochelins A (**1**) and B (**2**), from *Variovorax* sp. Their structures were elucidated by a combination of NMR, ESIMS/MS, and chemical derivatization. The analysis of the draft genome sequence of the H002 strain identified the variochelin biosynthetic gene cluster (*var*), which encodes PKS (polyketide synthase) and NRPS (non-ribosomal peptide synthetase) genes. Finally, the siderophores isolated in this study exhibited antibacterial activity against several bacteria, including soil-isolated plant pathogens.

## Results and Discussion

### Isolation and structure elucidation

**Figure 1:**
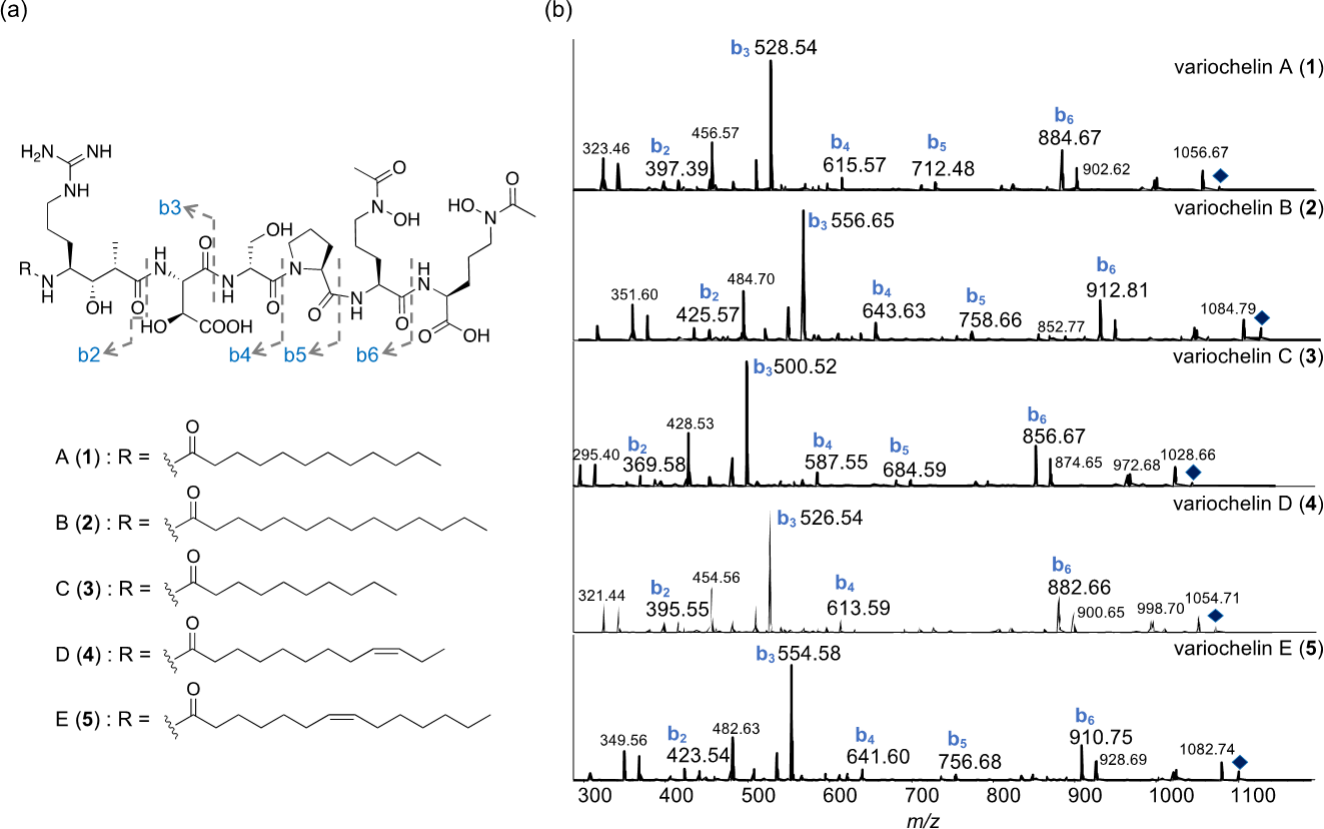
(a) Chemical structures and (b) ESIMS/MS of variochelins A–E (**1**–**5**) isolated from *Variovorax* sp. H002. Parent ions are *m*/*z* 1075.08 [M + H]^+^ (**1**), *m*/*z* 1103.18 [M + H]^+^ (**2**), *m*/*z* 1047.06 [M + H]^+^ (**3**), *m*/*z* 1072.95 [M + H]^+^ (**4**), and *m*/*z* 1101.14 [M + H]^+^ (**5**). Parent ions are represented by diamonds.

In this study, we explored the secondary metabolite potential of the soil-isolated *Variovorax* sp. H002, domesticated from the Medicinal Plant Garden of the Faculty of Pharmaceutical Sciences, Hokkaido University. To stimulate siderophore production, H002 was grown in modified minimal medium containing casamino acids and glucose as nitrogen and carbon sources, respectively, in the presence of HP-20 resin. Upon cultivation for one week, the non-polar resin was collected and washed thoroughly with water to remove salt and carbohydrate residues. After the retained siderophores were extracted with methanol, the obtained crude extract was further fractionated through several rounds of column chromatography, guided by a colorimetric-based chrome azurol sulfonate siderophore assay (CAS assay) [13], to yield five compounds (**1**–**5**, Figure 1). The production of these siderophores was high under iron-starvation conditions with 0.1 µM FeCl_3_ in the media, and began to decrease when the concentration of Fe^3+^ ions reached 0.5 µM (Figure S1, Supporting Information File 1).

The HRESIMS data of the major compound **1** showed a molecular weight of *m*/*z* 535.7912 for the [M − 2H]^2−^ ion, inferring a chemical formula of C_47_H_83_O_17_N_11_ (calcd 535.7911 for the double-negative ion). A combination of ^1^H NMR and COSY analyses revealed a peptidic structure comprising two *N*^δ^-acetyl-*N*^δ^-hydroxyornithine residues, a proline (Pro), a serine (Ser), a β-hydroxyaspartic acid (Hya), and a unique γ-amino acid biosynthetically originating from an arginine and a methyl malonate (4-amino-7-guanidino-3-hydroxy-2-methylheptanoic acid), with the amino group acylated by a saturated fatty acid. Subsequent HMBC and MS/MS analyses indicated that the planar structure of **1** is consistent with the known siderophore variochelin A, originally reported from *V. boronicumulans* BAM-48 by Nett et al. [5].

The NMR spectra of **2** are superimposable on those of **1**. However, the MS/MS of **2** differs in the fragmentation ion corresponding to a 4-acylamino-7-guanidino-3-hydroxy-2-methylheptanoate, indicating that the *N*-terminal fatty acyl group is a tetradecanoic acid in **2** (Figure 1 and Figure S10 in Supporting Information File 1). Therefore, compound **2** is concluded to be the known lipohexapeptide variochelin B [5].

**Figure 2:**
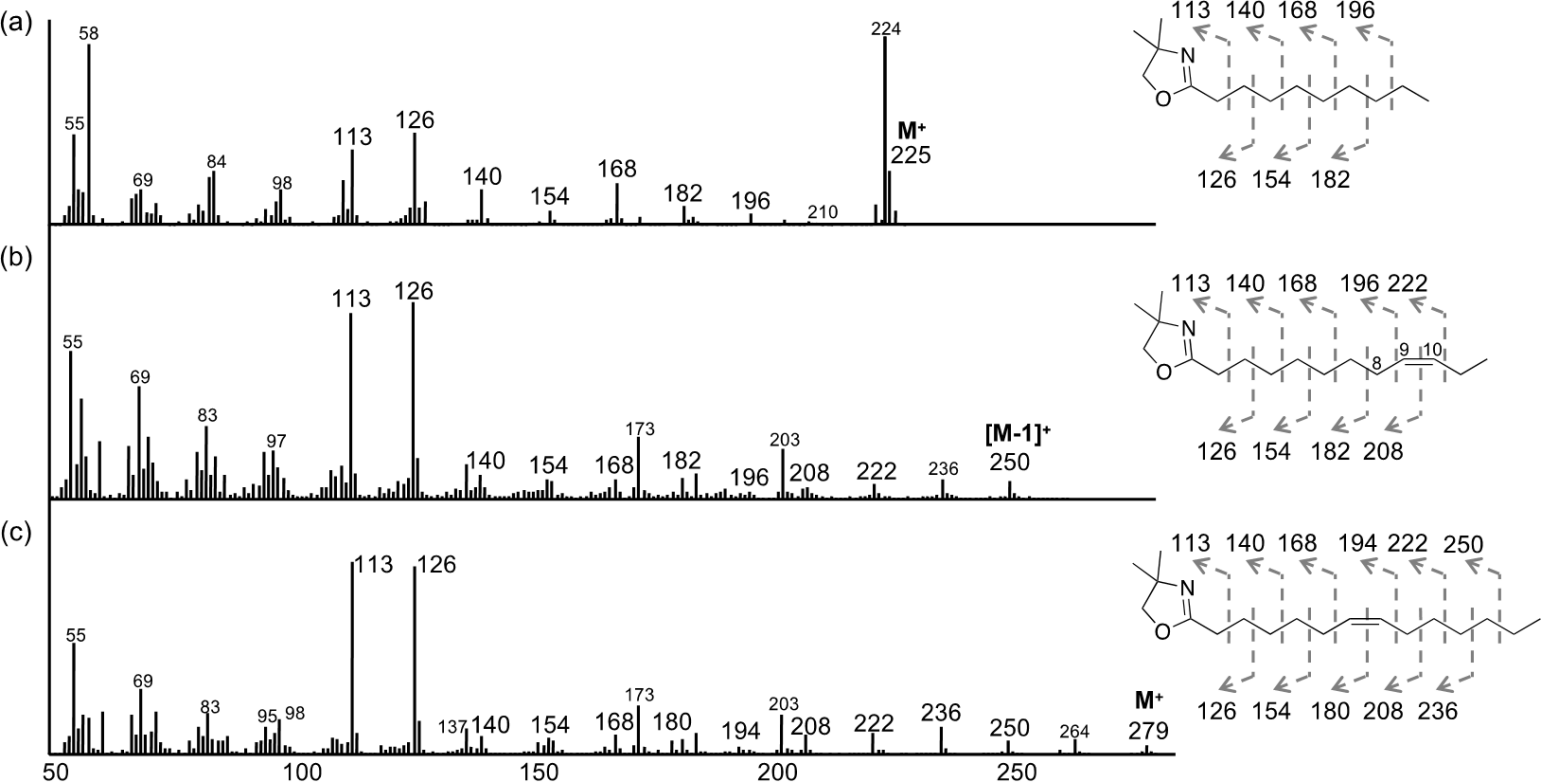
EIMS of DMOX derivatized free fatty acids derived from variochelins C–E (**3**–**5**). (a) **3**, (b) **4**, and (c) **5**.

Compound **3** has the chemical formula of C_45_H_79_O_17_N_11_, as suggested by the HRESIMS data (*m*/*z* 1044.5603 for the [M − H]^−^ ion), indicating the loss of two methylene groups (C_2_H_4_) from **1**. In addition, the MS/MS fragmentation profile of **3** was consistent with those of **1** and **2**, except for the loss of 28 mass units in the *N*-terminal *N*^γ^-acyl-γ-amino acid moiety (Figure 1 and Figure S18 in Supporting Information File 1). Subsequently, to determine the acyl chain, the hydrolysate of **3** was treated with 2-amino-2-methyl-1-propanol to yield a 4,4-dimethyloxazoline (DMOX) derivative of the fatty acid, which was then subjected to GC–MS [14–15]. The EIMS of the derivatized acyl moiety of **3** displayed two typical prominent ions at *m*/*z* 113 and *m*/*z* 126, which are diagnostic for the DMOX derivative [14–16] (Figure 2). The profile, with a molecular ion at *m*/*z* 225 [M]^+^ and the loss of each successive methylene group by *m*/*z* 196, 182, 168, 154, and 140, allowed us to deduce the fatty acyl moiety in **3** as an decanoic acid. Thus, we determined **3** to be a new siderophore termed variochelin C, with an *N*-terminal decanoate.

Compound **4** has the chemical formula of C_47_H_81_O_17_N_11_, as suggested by HRESIMS (*m*/*z* 534.7837 for [M − 2H]^2−^ ion), inferring the loss of two hydrogen atoms from **1**. The ^1^H NMR spectrum showed additional olefinic proton signals at δ_H_ 5.35, as compared with that of **1**. In line with this, two sp^2^ carbon signals at δ_C_ 130.6 and 131.0 were detected in the ^13^C NMR spectrum, demonstrating the presence of an olefin in **4**. The comparative analysis of the MS/MS profiles of **1** and **4** (Figure 1 and Figure S26 in Supporting Information File 1) revealed that all detected *b* ions in **4** were two mass units smaller than those in **1**, suggesting that the *N*-terminal acyl moiety is an unsaturated dodecenoic acid. To further confirm the acyl group, the DMOX derivative prepared from **4** was subjected to GC–MS analysis. The EIMS spectrum showed two typical prominent ions at *m*/*z* 113 and *m*/*z* 126, as well as two ions at *m*/*z* 222 and *m*/*z* 208, corresponding to the loss of methylene groups (14 mass units). More importantly, a gap of 12 mass units between *m*/*z* 196 (C8) and 208 (C9) was clearly detected, which indicated the position of the double bond between carbons C9 and C10 [14–15] and established the *N*-terminal acyl moiety in **4** as 9-dodecenoic acid. Therefore, **4** is a new analog, variochelin D, possessing an unsaturated fatty acyl moiety.

Finally, based on HRESIMS data, the chemical formula of **5** was deduced as C_49_H_85_O_17_N_11_ (1098.6071 [M − H]^−^, calcd. 1098.6052), indicating the loss of two hydrogen atoms from variochelin B (**2**). Similar to **4**, compound **5** also has a double bond in its fatty acyl moiety, as suggested by the MS/MS analysis (Figure 1 and Figure S34 in Supporting Information File 1). Subsequent GC–MS analysis of the DMOX derivative allowed us to deduce the fatty acyl chain in **5** as a 7-tetradecenoic acid, confirming **5** as another new variochelin analog, variochelin E.

The absolute configurations of the newly isolated congeners (**3**–**5**) were determined by Marfey’s analysis [17–18]. The absolute configurations of the amino acid constituents, Pro, Ser and two modified ornithine residues, in **3**–**5** were assigned as ʟ, ᴅ, and ʟ, respectively (Figures S25, S33, and S41, Supporting Information File 1). The geometry of the olefins in **4** and **5** was determined to be *Z* based on the ^13^C chemical shifts of allylic carbons [19].

Unfortunately, we were unable to determine the absolute configurations of Hya and three stereocenters in 4-amino-7-guanidino-3-hydroxy-2-methylheptanoic acid. ʟ-*threo*-Hya is reportedly present in **1**, based on the elution order of FDAA (*N*^α^-(5-fluoro-2,4-dinitrophenyl)-ʟ-alaninamide) derivatives [5]. In addition, a combination of NOESY and bioinformatics analyses proposed all three stereocenters of 4-amino-7-guanidino-3-hydroxy-2-methylheptanoic acid as *S* configured [5]. The stereochemistry of these moieties in **3**–**5** are discussed in the next section, regarding the variochelin biosynthetic gene cluster.

### Biosynthetic pathway of variochelins

**Figure 3:**
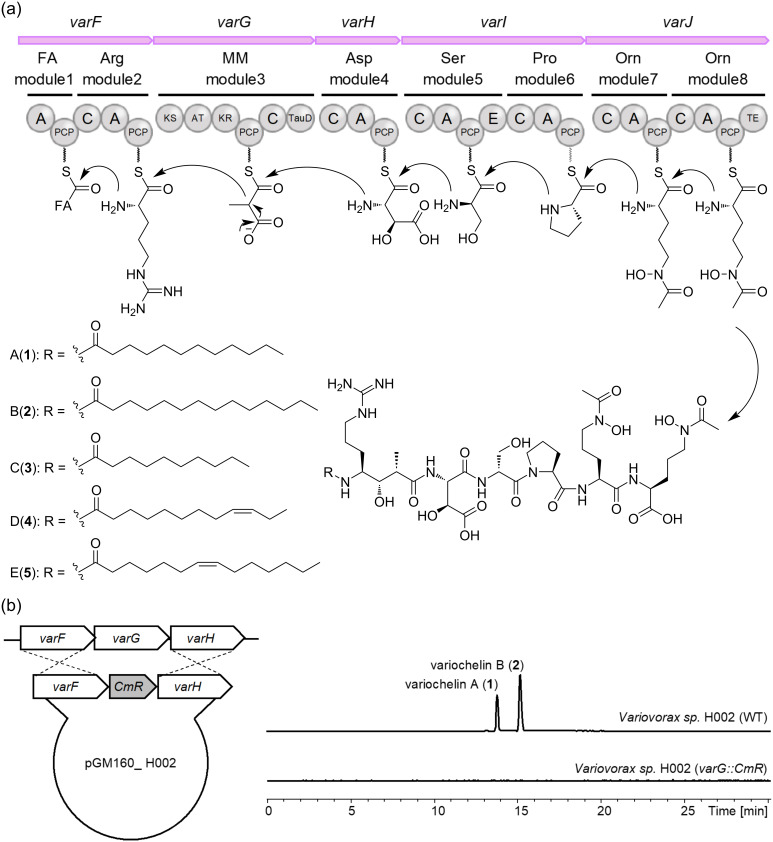
(a) Plausible biosynthetic pathway of variochelin A–E (**1**–**5**). The circles represent domains: A: adenylation domain, C: condensation domain, PCP: peptide carrier protein, E: epimerization domain, TE: thioesterase domain, KS: ketosynthase domain, AT: acyl transferase domain, KR: ketoreductase domain, TauD: taurine dioxygenase domain. Substrate specificity of A and KS domains is presented at the top of each module (FA: fatty acyl, Arg: arginine, MM: methyl malonyl, Asp: aspartic acid, Ser: serine, Pro: proline, Orn: ornithine). (b) Validation of *var* biosynthetic gene cluster. The plasmid constructed for the generation of the *varG* null-mutant strain (*varG::cmR*) using the *cmR* gene cassette (left), and HPLC-profile comparison of the *Variovorax* sp. H002 wild type strain and the *varG::cmR* strain monitored at 210 nm (right).

Nett et al. reported the variochelin biosynthetic gene cluster (NCBI accession number KT900023) encoded in *V. boronicumulans*, although it has not yet been experimentally validated. In the draft genome sequence of *Variovorax* sp. H002, we also identified a *var* gene cluster containing NRPS and PKS genes: the domain organizations of NRPS and PKS, and the adjacently encoded modification enzymes, were comparable to those of the gene cluster reported by Nett et al. with 92–99% identity at the protein level [5] (Figure 3a and Figure S42 in Supporting Information File 1). To investigate whether the identified *var* biosynthetic gene cluster is responsible for variochelin production, we substituted *varG* encoding the polyketide synthase (PKS) module with a chloramphenicol resistance (*cmR*) gene cassette, using homologous recombination (Figure 3b). As a result, the *varG* knock-out mutant no longer produced **1**–**5**, validating that the *var* biosynthetic gene cluster is indispensable for variochelin production in *Variovorax* sp. H002 (Figure 3b). As **1**–**5** share a common biosynthetic pathway, the Hya and the 4-amino-7-guanidino-3-hydroxy-2-methylheptanoic acid moieties in **3**–**5** have the same absolute configurations as those of **1**. In particular, the β-hydroxy group at the Asp residue is introduced by the hydroxylase domain (TauD) embedded in VarG. This type of hydroxylase reportedly generates ʟ-*erythro*-Hya, in agreement with the proposed structures of variochelins [20].

### Biological activity

The antimicrobial activities of **1**–**5** were evaluated against several Gram-negative and Gram-positive bacteria (Table 1). As a result, **1**–**5** inhibited the growth of the Gram-negative bacteria *Escherichia coli* and *Burkholderia multivorans*, but lacked activity against *Bacillus cereus* and *Kocuria rhizophila*. We subsequently investigated the antimicrobial properties of **1** against several known Gram-negative plant pathogens isolated from the same collection site as *Variovorax* sp. H002. Remarkably, variochelin A (**1**) moderately inhibited seven out of eight tested plant pathogens, suggesting that *Variovorax* sp. H002 modulates the local rhizosphere microbiome by utilizing variochelins (Table 2).

**Table 1:** Antibacterial activities of variochelins A (**1**), B (**2**), C (**3**), and E (**5**) against representative Gram-negative and Gram-positive bacteria.

	MIC (μg/mL)			

Compound	*E.coli*	*B. multivorans*	*B. cereus*	*K. rhizophila*

**1**	6.15	9.98	≥125	≥125
**2**	4.47	14.66	≥125	≥125
**3**	10.14	17.84	≥125	≥125
**5**	11.77	19.23	≥125	≥125

**Table 2:** Antibacterial activities of variochelin A (**1**) against some known soil-isolated plant pathogens.

Bacteria	MIC (μg/mL)

*Burkholderia plantarii*	8.49
*Burkholderia multivorans*	10.28
*Agrobacterium tumefaciens*	16.45
*Erwinia rhapontici*	≥125
*Pseudomonas syringae*	25.46

*Pantoea agglomerans*	21.47

*Ralstonia basilensis*	34.39

*Paraburkholderia* Sp. 60	29.03

## Conclusion

In this study, we reported the isolation of new variochelin congeners **3**–**5**, as well as the known variochelins **1** and **2**, from soil-isolated *Variovorax* sp. H002. The lipopeptide siderophores **1**–**5** share the same amino acid residues containing a combination of two hydroxamates and α-hydroxycarboxylate functional groups, which are capable of forming a photoreactive Fe(III) complex [5]. The common peptide headgroup with the additional repertoire of hydrophobic tails at the *N*-termini (**3**–**5**) resembles those of other amphiphilic marine siderophores such as marinobactins and aquachelins exhibiting the membrane affinity [21] as well as the self-assembling ability to form iron-containing vesicles [22]. This study presents another suite of amphiphilic peptidic siderophores originating in the terrestrial environment. Whether these siderophores play a role in iron acquisition through functions similar to or different from their marine counterparts is an intriguing question that will be answered by future studies.

## Experimental

### General experiments

All chemicals were purchased from Wako Pure Chemical Industries (Osaka, Japan), Kanto Chemicals (Tokyo, Japan) and Nacalai Tesque (Kyoto, Japan).

Optical rotations were measured on a JASCO P-1030 polarimeter. NMR spectra were recorded on a JEOL ECA 500 (500 MHz) spectrometer. Chemical shifts are denoted in δ (ppm) relative to residual solvent peaks as internal standards (CD_3_OD: δ_H_ 3.31, δ_C_ 49.0). LC–MS experiments and ESI–TOF MS/MS analyses were performed with an amaZon SL-NPC (Bruker Daltonics) or LCMS-2020 (Shimadzu) spectrometer coupled with a Shimadzu HPLC system equipped with an LC-20AD intelligent pump. GC–MS experiments were performed with a Shimadzu-QP2010 Ultra system. HRESIMS analyses were performed with a Thermo Scientific LTQ Orbitrap XL (LTQ XL coupled with LTQ QR) spectrometer.

### Isolation of variochelins A–E (**1**–**5**)

An overnight seed culture of *Variovorax* sp. H002 was inoculated into 100 mL freshly prepared modified minimal M9 media, containing M9 salts (0.7% K_2_HPO_4_, 0.2% KH_2_PO_4_, 0.01% MgSO_4_, 0.1% (NH_4_)_2_SO_4_, 0.06% NaCl), 1% glucose, 1% casamino acids, and HP20 resin (Diaion, Mitsubushi Chemical), in forty baffled acrylic flasks. After growth at 30 °C/140 rpm for seven days, the cultures were filtered through cotton to obtain the HP20 resin, which was packed into a column for chromatography. The packed resin was washed with water several times and then eluted with an excess of methanol. The collected methanol fraction was concentrated in vacuo to obtain a crude extract. Subsequently, the crude extract was subjected to Sephadex LH-20 column chromatography, with methanol as the mobile phase. After monitoring the fractions by a CAS assay, the positive fractions were concentrated and purified by semi-preparative HPLC on a Cosmosil 5C_18_-MS-II column (10 mm ID × 250 mm, Nacalai Tesque), using a gradient system with MeCN/H_2_O (3:7 to 7:3) as the mobile phase for 30 minutes to yield variochelins A (**1**, 10.0 mg), B (**2**, 20.1 mg), C (**3**, 3.0 mg), D (**4**, 2.1 mg), and E (**5**, 5.3 mg).

Variochelin A (**1**) HRESIMS *m*/*z*: [M − 2H]^2−^, calcd for C_47_H_83_O_17_N_11_, 535.7911; found, 535.7912, Δ 0.2 ppm for C_47_H_83_O_17_N_11_; 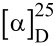 −7.5, (*c* 1.78, MeOH), ^1^H and ^13^C NMR chemical shifts see Table S1 (Supporting Information File 1).

Variochelin B (**2**) HRESIMS *m*/*z*: [M − H]^−^ calcd for C_49_H_87_O_17_N_11_, 1100.6209; found, 1100.6218, Δ 0.8 ppm for; C_49_H_87_O_17_N_11_; 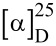 −11.1, (*c* 0.23, MeOH), ^1^H and ^13^C NMR chemical shifts see Table S1 (Supporting Information File 1).

Variochelin C (**3**) HRESIMS *m*/*z*: [M − H]^−^ calcd for C_45_H_79_O_17_N_11_, 1044.5583; found, 1044.5603, Δ 1.9 ppm for C_45_H_79_O_17_N_11_; 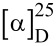 −13.8, (*c* 0.25, MeOH), ^1^H and ^13^C NMR chemical shifts see Table S1 (Supporting Information File 1).

Variochelin D (**4**) HRESIMS *m*/*z*: [M − 2H]^2−^ calcd for C_47_H_81_O_17_N_11_, 534.7833; found, 534.7837, Δ 0.8 ppm for C_47_H_81_O_17_N_11_; 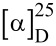 −20.9, (*c* 0.19, MeOH), ^1^H and ^13^C NMR chemical shifts see Table S1 (Supporting Information File 1).

Variochelin E (**5**) HRESIMS *m*/*z*: [M − H]^−^ calcd for C_49_H_85_O_17_N_11_, 1098.6052; found, 1098.6071, Δ 1.7 ppm for C_49_H_85_O_17_N_11_; 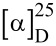 −19.7, (*c* 0.21, MeOH), ^1^H and ^13^C NMR chemical shifts see Table S1 (Supporting Information File 1).

### Preparation of fatty acid methyl ester (FAME) derivatives

0.1 mg of **1** or **2** were hydrolyzed with 6 M HCl at 110 °C overnight. The obtained hydrolysates were freeze dried, resuspended in 100 µL of methanol, and then treated with 100 µL of trimethylsilyldiazomethane, and the mixtures were kept at room temperature for one hour. The reaction mixtures were concentrated in vacuo, dissolved in acetone, and then injected into a GC–MS system, Zebron ZB-WAX (30 m × 0.25 mm ID × 0.25 µm, Phenomenex), using He as the gas source at a flow rate of 1.41 mL/min. The initial temperature (120 °C) was held for one minute, increased to 230 °C at a rate of 10 °C/min, and then held at 230 °C for six minutes.

### Preparation of 2-alkenyl-4,4-dimethyloxazoline (DMOX) fatty acid derivatives

0.2 mg of **3**–**5** were hydrolyzed with 6 M HCl at 110 °C overnight. The obtained hydrolysates were treated with 250 µL of 2-amino-2-methylpropanol in screw-capped vials. After flushing with nitrogen, the vials were sealed and heated at 180 °C for 18 hours. The reaction mixtures were then cooled down, resuspended in 3 mL of dichloromethane, and washed three times with 1 mL of water. The organic layer was dried with anhydrous Na_2_SO_4_ and concentrated in vacuo. The samples were redissolved in acetone, and then injected into the aforementioned GC–MS system.

### Determination of amino acid configuration by Marfey’s method

A 100 µg portion of each compound (**3**–**5**) was hydrolyzed with 6 M HCl at 110 °C overnight. The lyophilized hydrolysates were dissolved in water (160 µL), and treated with saturated NaHCO_3_ (200 µL) and 1% ʟ-FDLA in acetone (160 µL). The mixtures were heated at 40 °C for 30 minutes, quenched with 1 M HCl (200 µL), and concentrated in vacuo. The residue was dissolved in DMSO (400 µL) and passed through a membrane filter, and then injected into an LC–MS system, Cosmosil 5C_18_-MS-II (2 mm ID × 100 mm, Nacalai Tesque) (oven 40 °C; flowrate 0.2 mL/min) using a gradient of MeCN/H_2_O (1:9 to 6:4) containing 0.1% formic acid for 45 minutes. The standards were processed in the same manner.

### Whole genome analysis of *Variovorax* sp. H002

*Variovorax* sp. H002 was streaked onto a 2R2A agar plate and grown at 30 °C overnight. The resulting colonies were inoculated into 5 mL of LB in a 15 mL Falcon tube. After growth at 30 °C/140 rpm for 1 day, the culture was pelleted by centrifugation. The cell pellet was incubated at 37 °C for 1 h with 10 mg/mL lysozyme. After 10% SDS was added to the tube, the mixture was incubated at room temperature, 60 °C, and then 0 °C, for 5 min durations. AcOK (5 M) and phenol/CHCl_3_/isoamyl alcohol (25:24:1) were then added, and the resulting solution was gently mixed by inversion and centrifuged at 7,000*g* for 10 min. The upper aqueous phase was collected in a new tube. This partitioning step was repeated twice. Isopropanol was added to the aqueous phase obtained after AcOK treatment, and the solution was mixed by inversion and centrifuged at 7,000*g* for 10 min. The pellet was washed with 70% EtOH, dried, and dissolved in TE buffer. The extracted genomic DNA was quantified by a Qubit v3.0 fluorometer (Life Technologies, Thermo Fisher Scientific, Inc.).

The *Variovorax* sp. H002 genome was sequenced by a DNBSEQ for short-lead sequencing and an Oxford nanopore GridION X5 for long-lead sequencing.

For the short-lead sequencing, the library was prepared using 100 ng of the gDNA with an MGIEasy FS DNA Library Prep Set (MGI), following the manufacturer’s protocol. The gDNA was fragmented enzymatically to approximately 400 bp. As a result of sequencing (150 bp × 2) and quality filtering (Q > 30), a total of 1.4 Gbp reads were obtained. The genome size of *Variovorax* sp. H002 was roughly estimated to be 8 Mb.

For the long-lead sequencing, 1,000 ng of the gDNA was treated with Short Read Eliminator XS (Circulomics) to remove fragments shorter than 10 kb. The resultant gDNA was subjected to library preparation with a Ligation Sequencing Kit (SQK-LSK 109), following the 1D genomic DNA by ligation protocol. The library was applied to a MinION flowcell (FLO MIN106 R9.41revD) operated by the MinKNOW (20.06.9) software, and then processed by Guppy basecaller (4.0.11) in the high accuracy mode. As a result, a total of 0.4 Gbp reads were obtained.

The reads were subjected to hybrid de novo assembly using SPAdes (v0.4.8) [23]. The assemblies were subjected to BGC prediction using antiSMASH (6.0.1) [24].

### Generation of Δ*varG* mutant

The 1 kb upstream and downstream regions flanking *varG* were amplified by PCR, using *Variovorax* sp. H002 genomic DNA as the template. The chloramphenicol-resistance gene (*cmR*) and a linearized recipient plasmid pRED were amplified by PCR, using pRED as the template. The primer sets used to amplify the above-mentioned four fragments are listed in Table S2 (Supporting Information File 1). The four fragments were combined by Gibson Assembly Reaction (New England Biolabs) to generate a disruption cassette: upstream-*cmR*-downstream. After the transformation of *E. coli* DH5α with the cassette-pRED, a *HindIII* site was inserted on the end of the cassette sequence to yield the disruption plasmid, pGM160-Δ*varG*. The cassette was sequenced for validation. The disruption plasmid was introduced into *E coli* S17-1 λpir by electroporation.

Both *Variovorax* sp. H002 and *E. coli* S17-1 λpir were cultured overnight at 30 °C in 2xR2A and LB, respectively. The overnight cultures were washed with 2xR2A three times, and the pellets were resuspended with 500 mL of 2xR2A. The OD_600_ values of the donor (*E. coli* S17-1 λpir) and recipient (*Variovorax* sp. H002) cells were set to 2.0 and mixed in a 1:1 ratio. The mixtures were spotted onto 2xR2A plates and incubated at 30 °C for two days. The colonies were collected and spread onto 2xR2A agar plates with chloramphenicol (30 µg/mL) and kanamycin (50 µg/mL). The plates were incubated at 30 °C until colonies formed. The disruption of *varG* was confirmed by PCR amplification.

### Effect of ferric ion (Fe^3+^) concentration on production of variochelins

An overnight culture of *Variovorax* sp. H002 in 2xR2A liquid media was transferred to small baffled acrylic flasks containing 15 mL of freshly prepared modified M9 media fortified with 0, 0.5, 1, 5, 10, or 50 µM (final concentration) of FeCl_3_·6H_2_O. Upon cultivation at 30 °C/140 rpm for seven days, the cultures were centrifuged, and the supernatants were applied to pre-washed Sep-Pak C_18_ cartridges, which were then washed with water and eluted with methanol. The collected methanolic fractions were dried and resuspended in 0.5 mL of methanol. The particulate-free sample solutions (up to 100 µL) were injected into a Cosmosil 5C_18_-MS-II column (10 mm ID × 250 mm, Nacalai Tesque) attached to an HPLC with an autosampler, at a flow rate of 0.8 mL/min at 40 °C. Each analysis was performed using an aqueous acetonitrile mobile phase.

### Biological activities of variochelins

Assays for all compounds were performed in triplicate, according to the Clinical Laboratory Standards Institute testing standard, in 96-well plate microbroth dilution assays. Compounds were tested against Gram-negative bacteria (*Escherichia coli* JW5503, *Bukholderia multivorans* NBRC 102086 and *Bukholderia plantarii* NBRC 104884) and Gram-positive bacteria *(Bacillus cereus* NBRC 15305 and *Kocuria rhizophila* NBRC 12708). In addition, several in-house collections of plant pathogens including *Erwinia rhapontici*_D020, *Pantoea agglomerans*_G054_1, *Agrobacterium tumefaciens*_K021_1, *Pseudomonas syringae*_E029, *Ralstonia basilensis*_M004 and *Paraburkholderia* Sp. 60 isolated from the same location (Botanical Garden of Faculty of Pharmacy Hokkaido University) with *Variovorax* sp. H002 were selected as tested bacteria against variochelin A (**1**). Briefly, the overnight seed cultures of tested bacteria in Mueller–Hinton (MH) media were diluted with a fresh MH media to obtain a final OD_600_ of 0.05. The diluted bacteria were subsequently transferred into a 96-well plate (100 µL/well) and exposed to an appropriate concentration of variochelins. After an incubation at 30 °C for 22 h, the optical density was recorded at 600 nm using plate reader, to determine percent growth inhibition.

## Supporting Information

File 1 Additional figures and tables, NMR and MS spectra.

## Data Availability

All data that supports the findings of this study is available in the published article and/or the supporting information to this article.
