# Supplementary material for: New variochelins from soil-isolated Variovorax sp. H002
Source: Beilstein J Org Chem. 2024 Apr 2;20:692–700. doi: 10.3762/bjoc.20.63 (PMC10999976; doi:10.3762/bjoc.20.63)
Supplement: File 1 — Additional figures and tables, NMR and MS spectra. [file Beilstein_J_Org_Chem-20-692-s001.pdf]

BEILSTEIN JOURNAL OF ORGANIC CHEMISTRY

Supporting Information

for

New variochelins from soil-isolated *Variovorax* sp. H002

Jabal Rahmat Haedar, Aya Yoshimura and Toshiyuki Wakimoto

Beilstein J. Org. Chem. **2024,** *20,* 692–700. doi:10.3762/bjoc.20.63

Additional figures and tables, NMR and MS spectra

License and Terms: This is a supporting information file under the terms of the Creative Commons Attribution License (https://creativecommons.org/licenses/by/4.0). Please note that the reuse, redistribution and reproduction in particular requires that the author(s) and source are credited and that individual graphics may be subject to special legal provisions.
The license is subject to the *Beilstein Journal of Organic Chemistry* terms and conditions: (https://www.beilstein-journals.org/bjoc/terms)

Table of contents

Figure S1. Effect of various iron concentrations on variochelin B (**2**) production...........S3
Figure S2. ESIMS/MS spectrum of variochelin A (**1**).. S4
Figure S3. ^{1}H NMR spectrum of variochelin A (**1**) (500 MHz, DMSO-d_6)......................... S5
Figure S4. ^{1}H NMR spectrum of variochelin A (**1**) (500 MHz, methanol-d_4)...................... S6
Figure S5. ^{13}C NMR spectrum of variochelin A (**1**) (125 MHz, methanol-d_4)..................... S7
Figure S6. COSY spectrum of variochelin A (**1**) (500 MHz, methanol-d_4).......................... S8
Figure S7. HSQC spectrum of variochelin A (**1**) (500 MHz, methanol-d_4).......................... S9
Figure S8. HMBC spectrum of variochelin A (**1**) (500 MHz, methanol-d_4) S10
Figure S9. EIMS of dodecanoic acid methyl ester in variochelin A (**1**) S11
Figure S10. ESIMS/MS spectrum of variochelin B (**2**) S12
Figure S11. ^{1}H NMR spectrum of variochelin B (**2**) (500 MHz, DMSO-d_6)....................... S13
Figure S12. ^{1}H NMR spectrum of variochelin B (**2**) (500 MHz, methanol-d_4) S14
Figure S13. ^{13}C NMR spectrum of variochelin B (**2**) (125 MHz, methanol-d_4).................. S15
Figure S14. COSY spectrum of variochelin B (**2**) (500 MHz, methanol-d_4)........................ S16
Figure S15. HSQC spectrum of variochelin B (**2**) (500 MHz, methanol-d_4)........................ S17
Figure S16. HMBC spectrum of variochelin B (**2**) (500 MHz, methanol-d_4)....................... S18
Figure S17. EIMS of tetradecanoic acid methyl ester in variochelin B (**2**) S19
Figure S18. ESIMS/MS spectrum of variochelin C (**3**) S20
Figure S19. ^{1}H NMR spectrum of variochelin C (**3**) (500 MHz, DMSO-d_6)....................... S21
Figure S20. ^{1}H NMR spectrum of variochelin C (**3**) (500 MHz, methanol-d_4) S22
Figure S21. ^{13}C NMR spectrum of variochelin C (**3**) (125 MHz, methanol-d_4).................. S23
Figure S22. COSY spectrum of variochelin C (**3**) (500 MHz, methanol-d_4)........................ S24
Figure S23. HSQC spectrum of variochelin C (**3**) (500 MHz, methanol-d_4)........................ S25
Figure S24. HMBC spectrum of variochelin C (**3**) (500 MHz, methanol-d_4)....................... S26
Figure S25. Marfey's analysis of variochelin C (**3**)... S27
Figure S26. ESIMS/MS spectrum of variochelin D (**4**)... S28
Figure S27. ^{1}H NMR spectrum of variochelin D (**4**) (500 MHz, DMSO-d_6) S29
Figure S28. ^{1}H NMR spectrum of variochelin D (**4**) (500 MHz, methanol-d_4)................... S30
Figure S29. ^{13}C NMR spectrum of variochelin D (**4**) (125 MHz, methanol-d_4).................. S31
Figure S30. COSY spectrum of variochelin D (**4**) (500 MHz, methanol-d_4)........................ S32
Figure S31. HSQC spectrum of variochelin D (**4**) (500 MHz, methanol-d_4)........................ S33
Figure S32. HMBC spectrum of variochelin D (**4**) (500 MHz, methanol-d_4) S34
Figure S33. Marfey's analysis of variochelin D (**4**)... S35
Figure S34. ESIMS/MS spectrum of variochelin E (**5**) S36
Figure S35. ^{1}H NMR spectrum of variochelin E (**5**) (500 MHz, methanol-d_4) S37
Figure S36. ^{1}H NMR spectrum of variochelin E (**5**) (500 MHz, DMSO-d_6)....................... S38
Figure S37. ^{13}C NMR spectrum of variochelin E (**5**) (125 MHz, methanol-d_4) S39
Figure S38. COSY spectrum of variochelin E (**5**) (500 MHz, methanol-d_4)........................ S40
Figure S39. HSQC spectrum of variochelin E (**5**) (500 MHz, methanol-d_4)........................ S41
Figure S40. HMBC spectrum of variochelin E (**5**) (500 MHz, methanol-d_4)....................... S42
Figure S41. Marfey's analysis of variochelin E (**5**) S43
Figure S42. Comparison of Variochelin BGCs identified in
Variovorax boronicumulans BAM-48 and *Variovorax* sp. H002.................... S44

Figure S43. Validation of *var* biosynthetic gene cluster in *Variovorax* sp. H002 .... S45
Figure S44. Extracted ion chromatogram showing deletion of *varG* abolishes variochelin A–E (**1**–**5**) production in *Variovorax* sp. H002. .... S46
Figure S45. Selected COSY and HMBC correlations of variochelin A–E (**1**–**5**) in methanol-d_4 .... S47
Table S1. ^{1}H (500 MHz) and ^{13}C (125 MHz) NMR data of variochelins A–E (**1**–**5**) in methanol-d_4 (δ in ppm).. S48
Table S2. Primers, strains and plasmids used in this study.. S52
Table S3. Annotation of *var* biosynthetic gene cluster in *Variovorax* sp. H002 .... S53

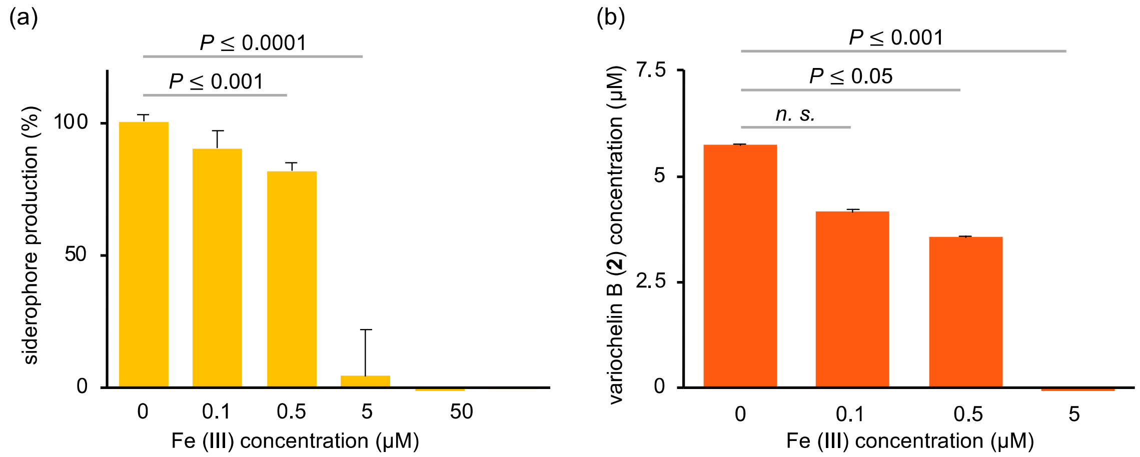

Figure S1. The effect of various iron concentrations on (a) total siderophore production indicated by CAS assay activity (n = 3), and (b) production of variochelin B (**2**) indicated by calculation of total area under curve (n = 3).

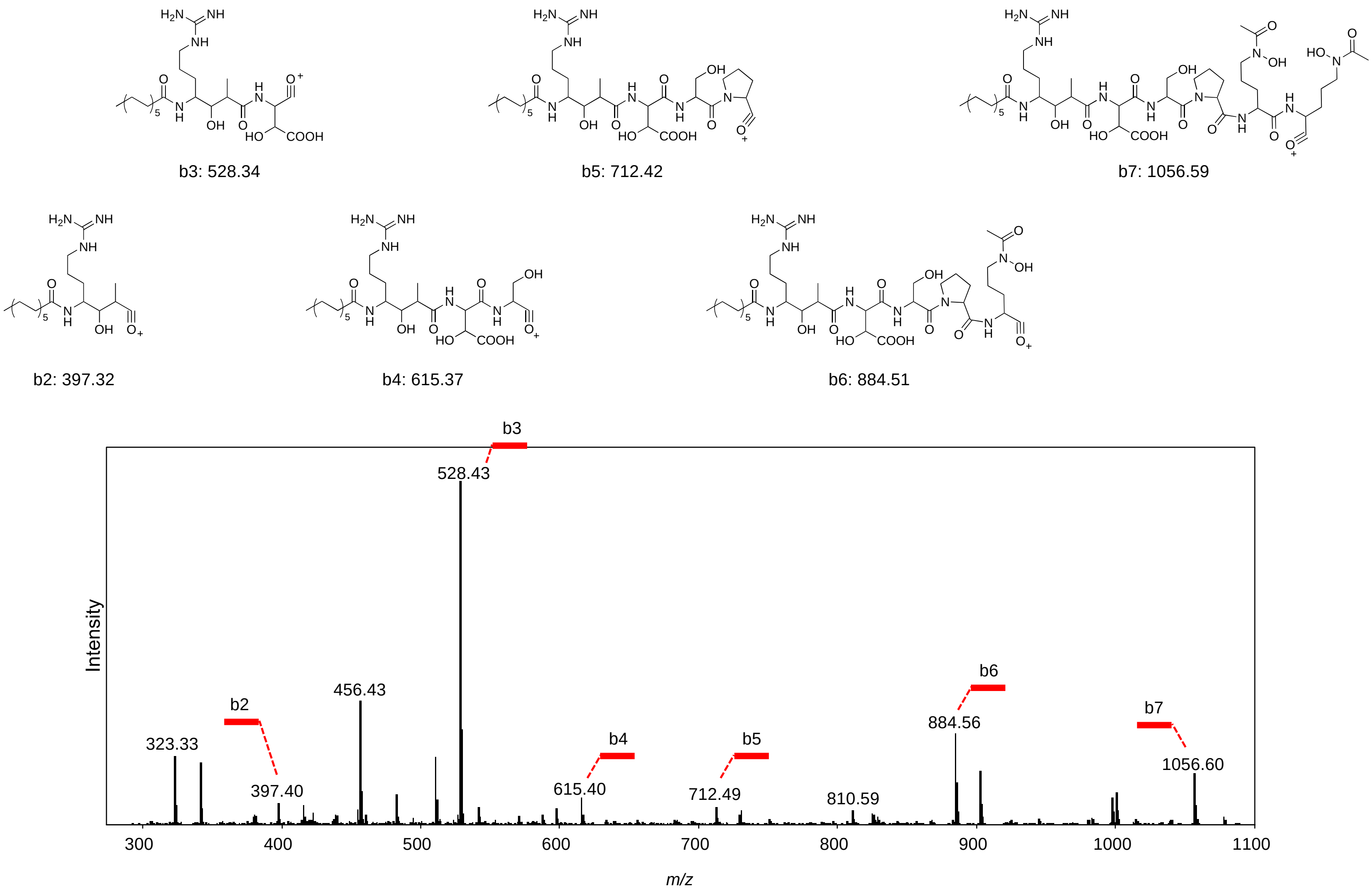

Figure S2. ESIMS/MS analysis of variochelin A (**1**). Structures and *m/z* of predicted fragmentation ions were described the top. An ion peak of $[M + H]^+$ at *m/z* 1075.08 was chosen as a parent ion.

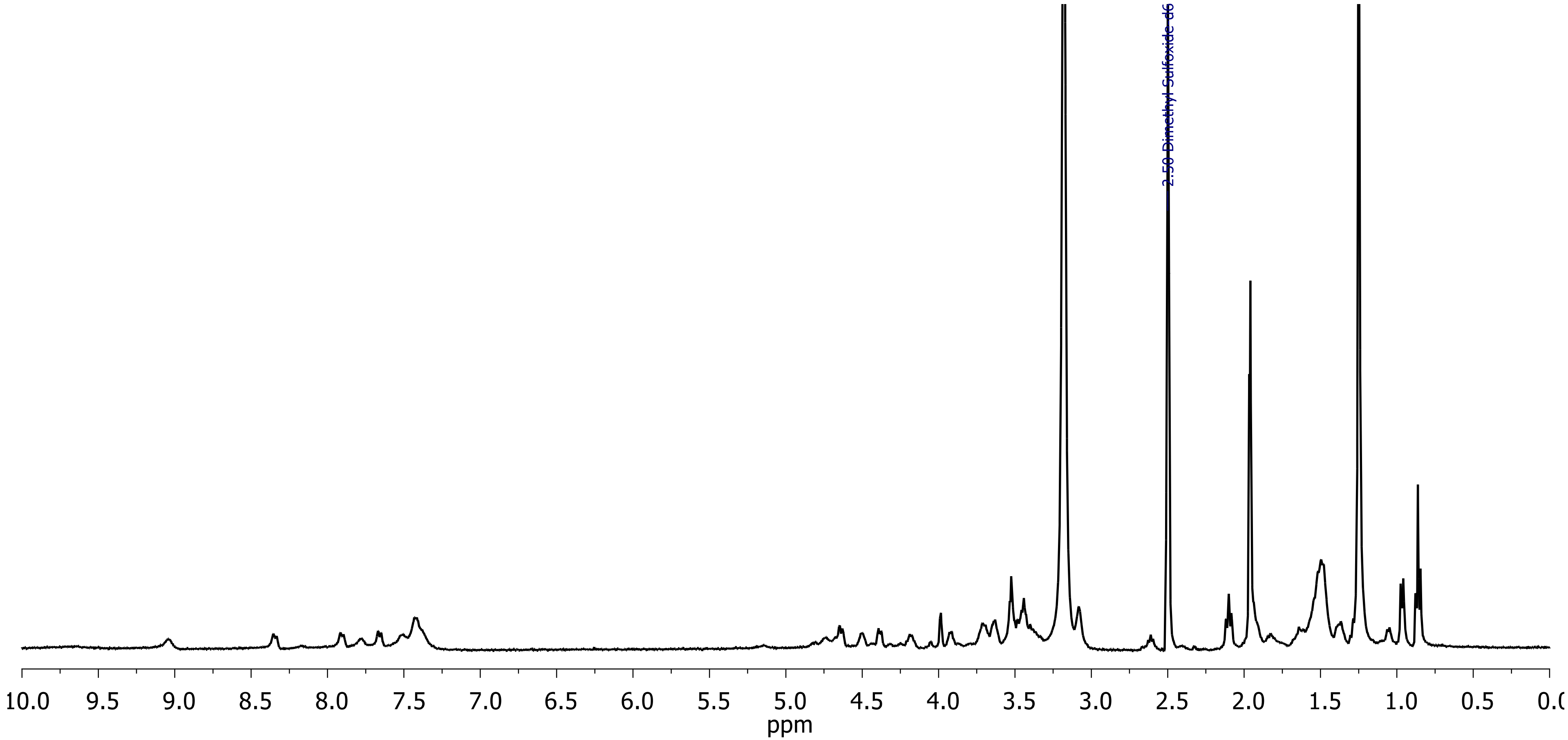

Figure S3. ^{1}H NMR spectrum of variochelin A (**1**) (500 MHz, DMSO-d_6).

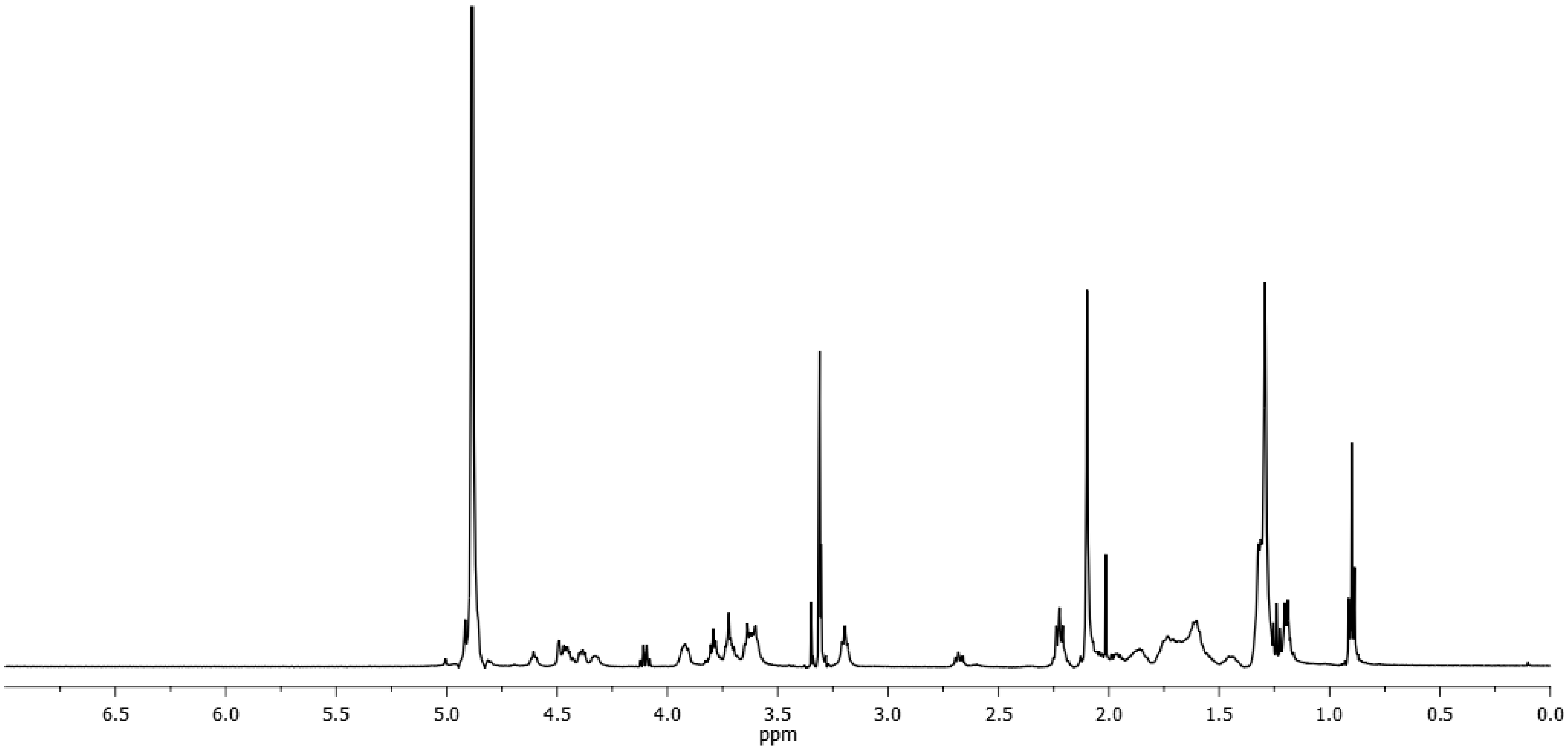

Figure S4. ^{1}H NMR spectrum of variochelin A (**1**) (500 MHz, methanol-d_4).

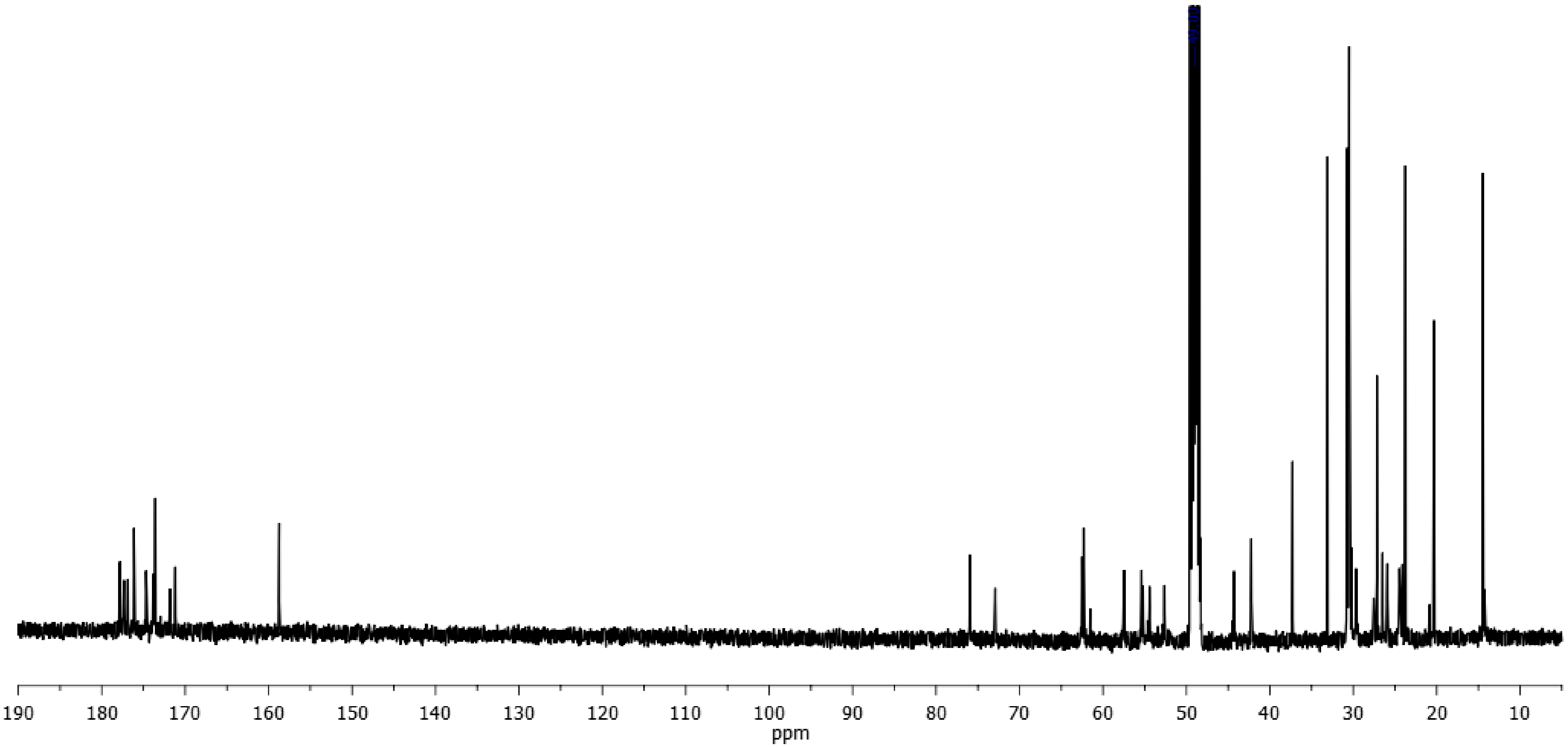

Figure S5. ^{13}C NMR spectrum of variochelin A (**1**) (125 MHz, methanol-d_4).

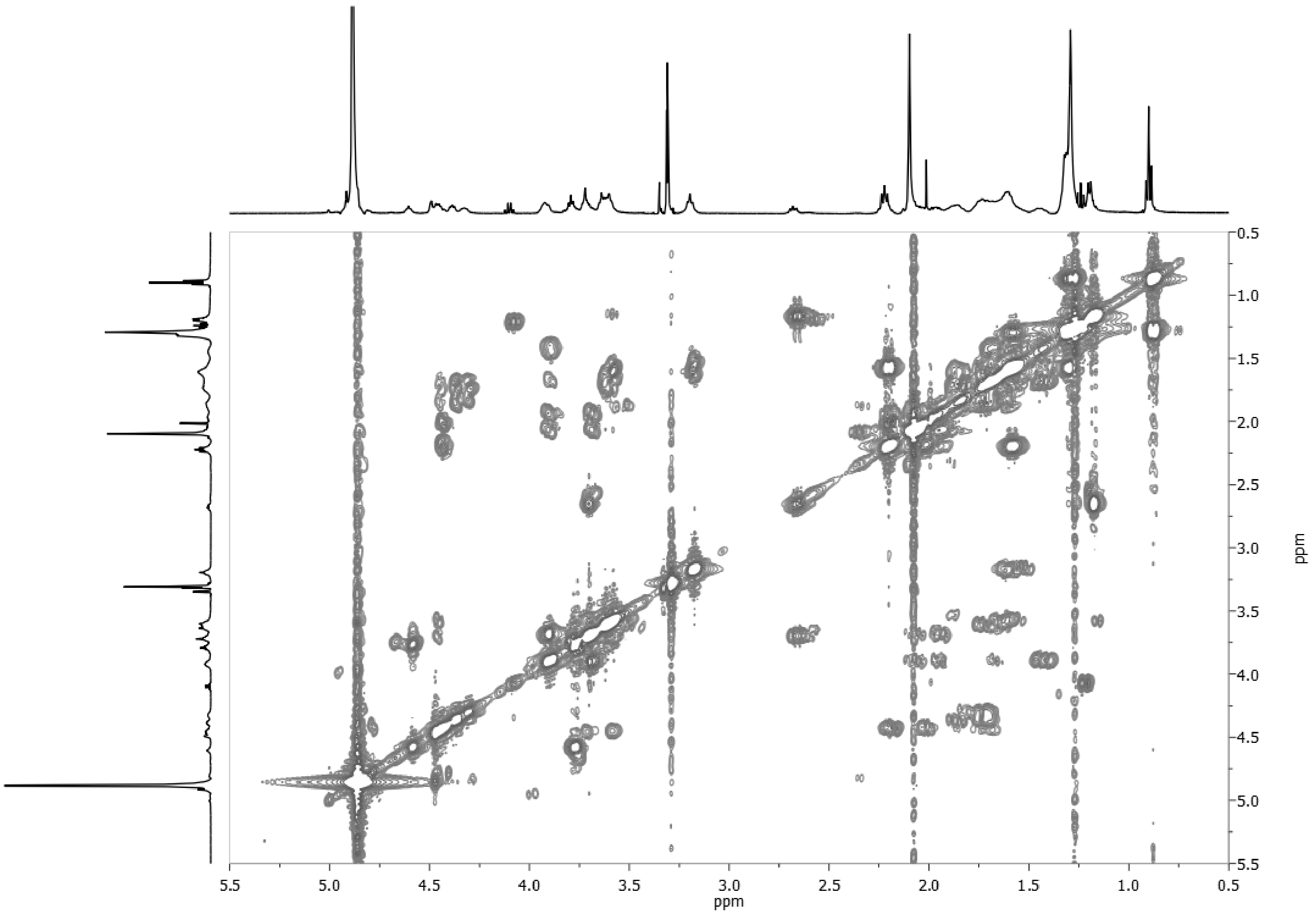

Figure S6. COSY spectrum of variochelin A (**1**) (500 MHz, methanol-d_4).

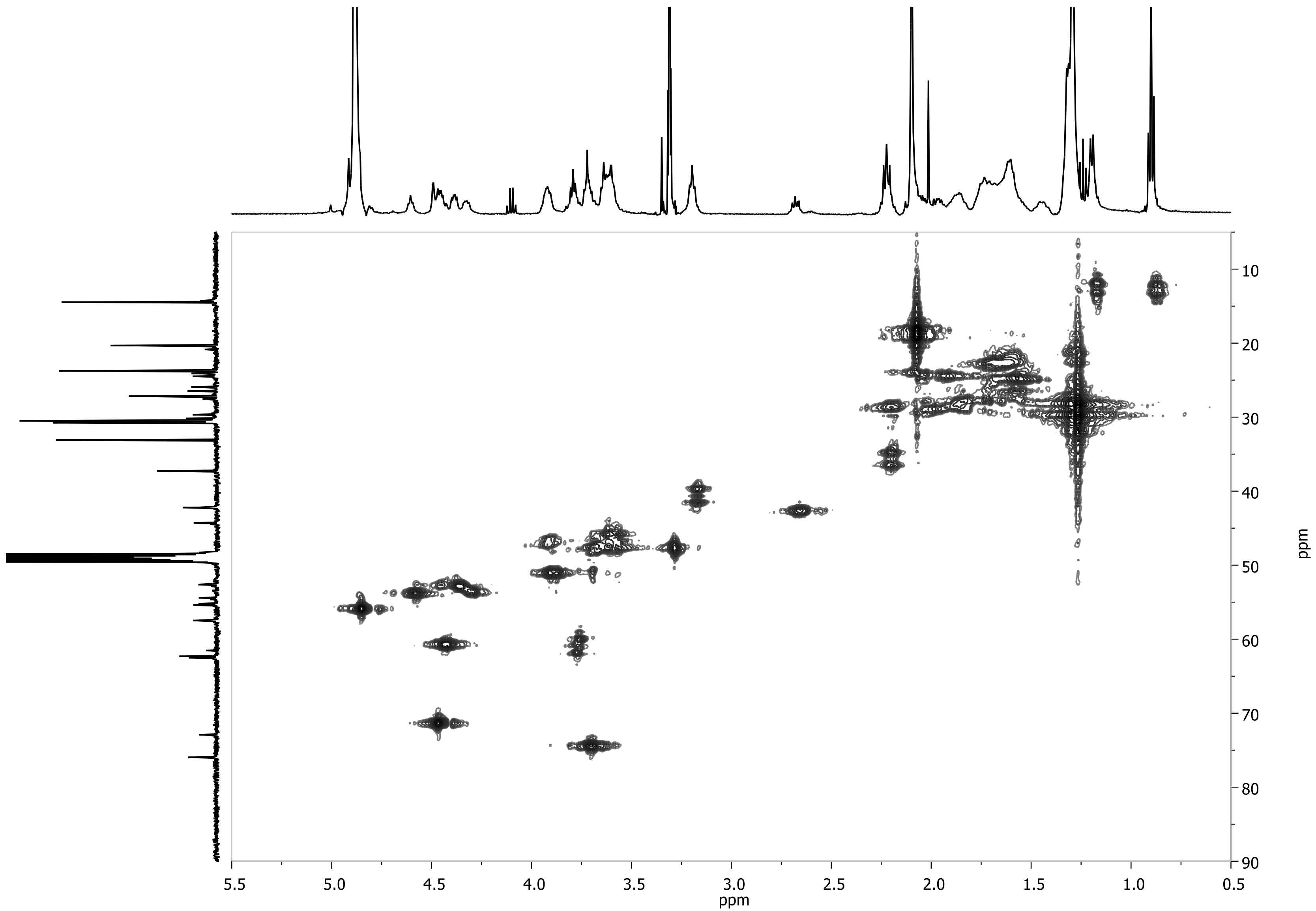

Figure S7. HSQC spectrum of variochelin A (**1**) (500 MHz, methanol-d_4).

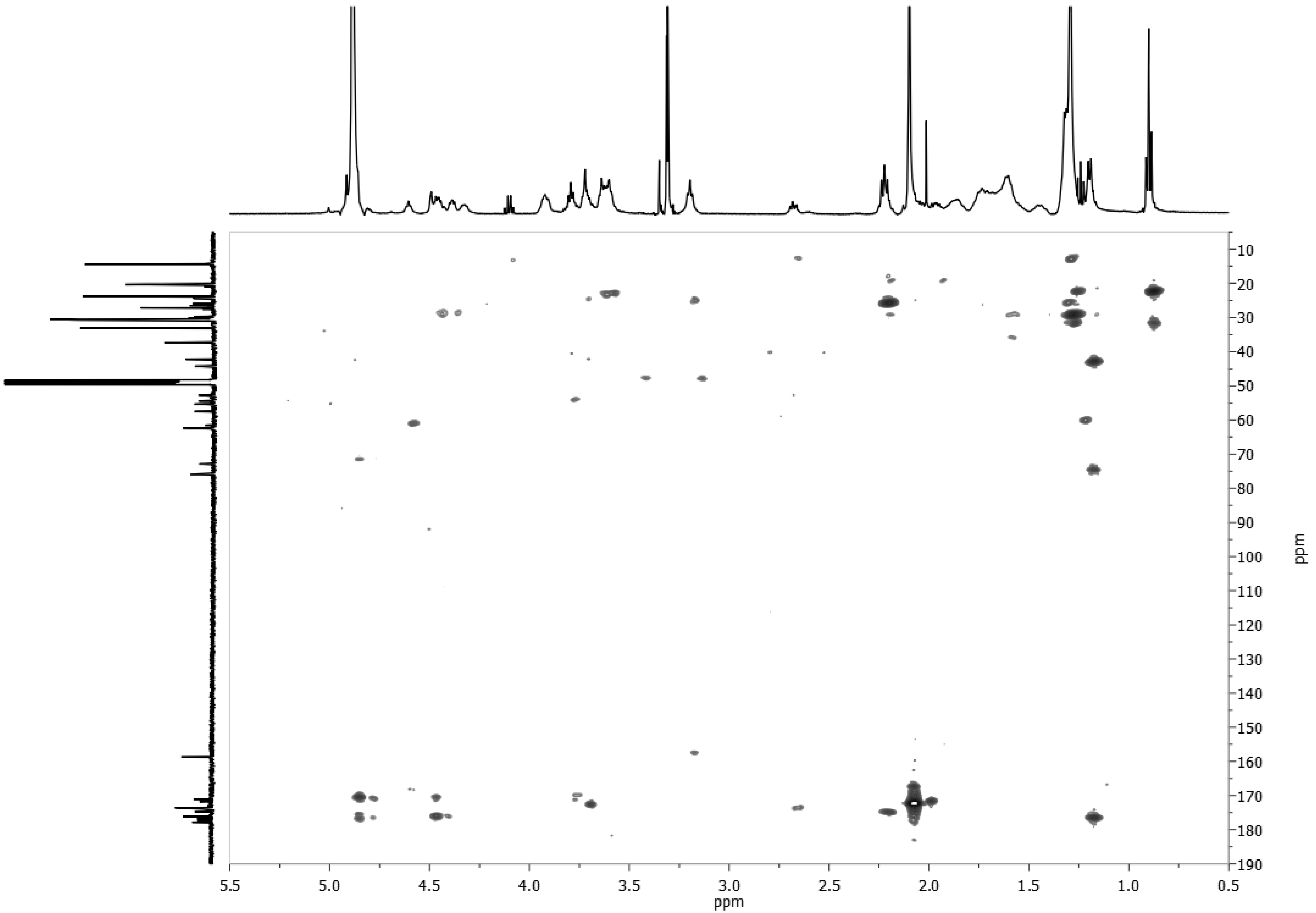

Figure S8. HMBC spectrum of variochelin A (**1**) (500 MHz, methanol-d_4).

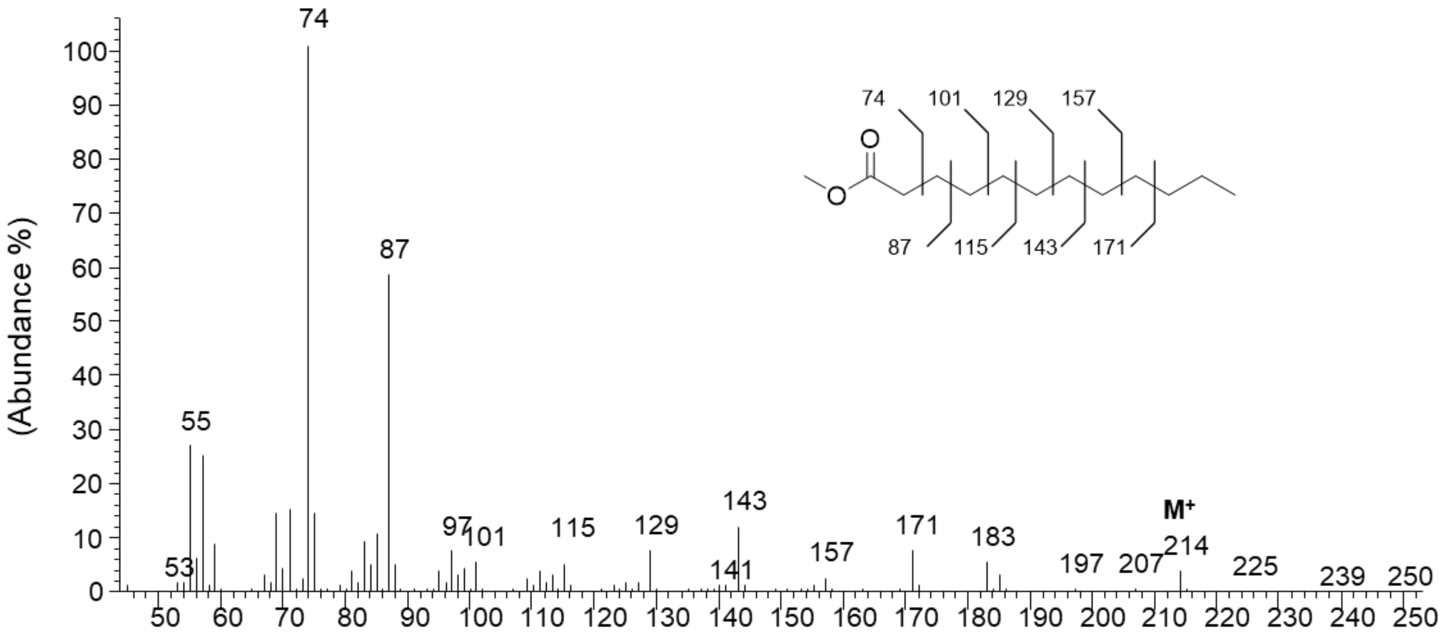

Figure S9. EIMS of dodecanoic acid methyl ester in variochelin A (**1**).

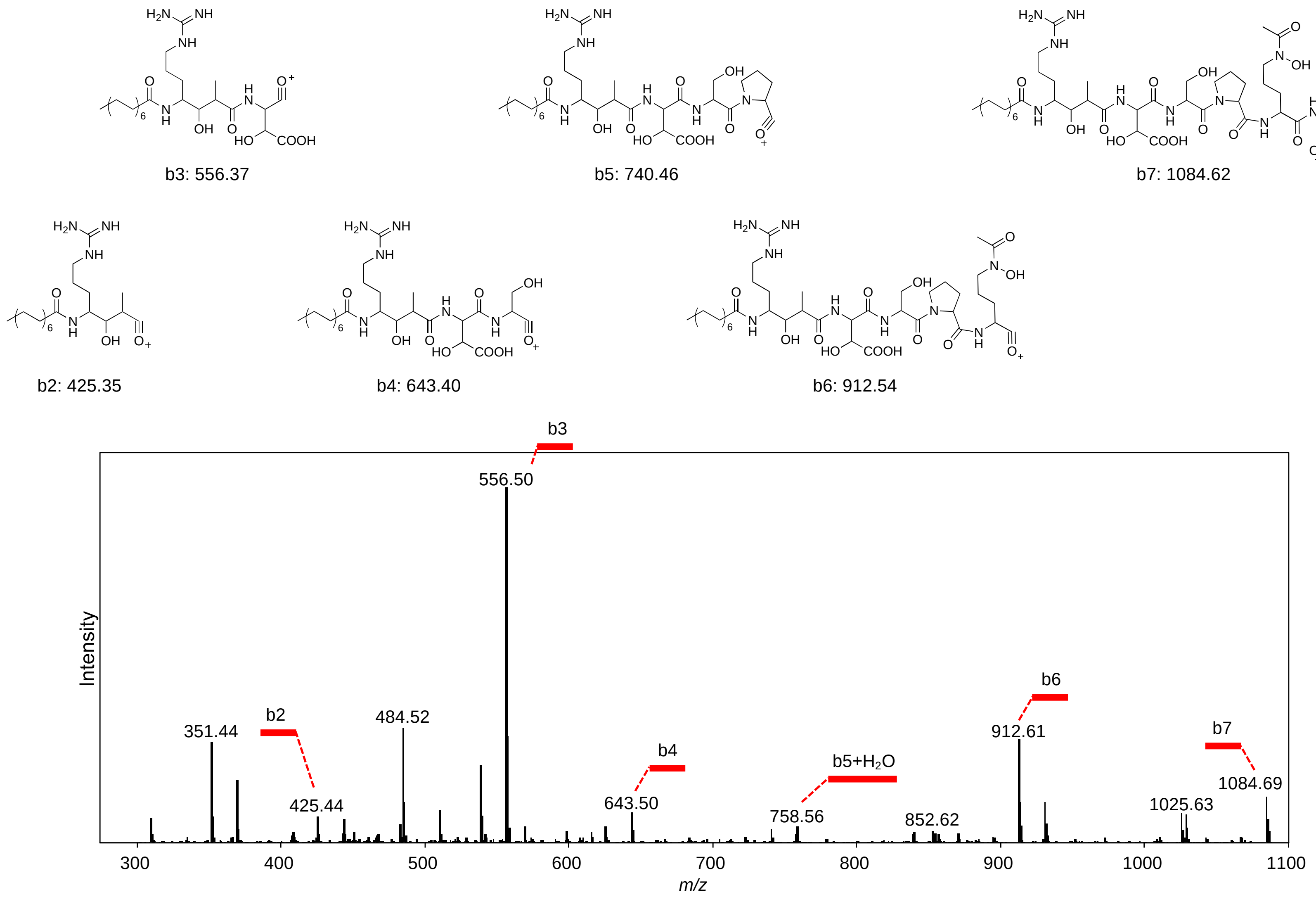

Figure S10. ESIMS/MS analysis of variochelin B (**2**). Structures and *m/z* of predicted fragmentation ions were described the top. An ion peak of $[M + H]^+$ at *m/z* 1103.18 was chosen as a parent ion.

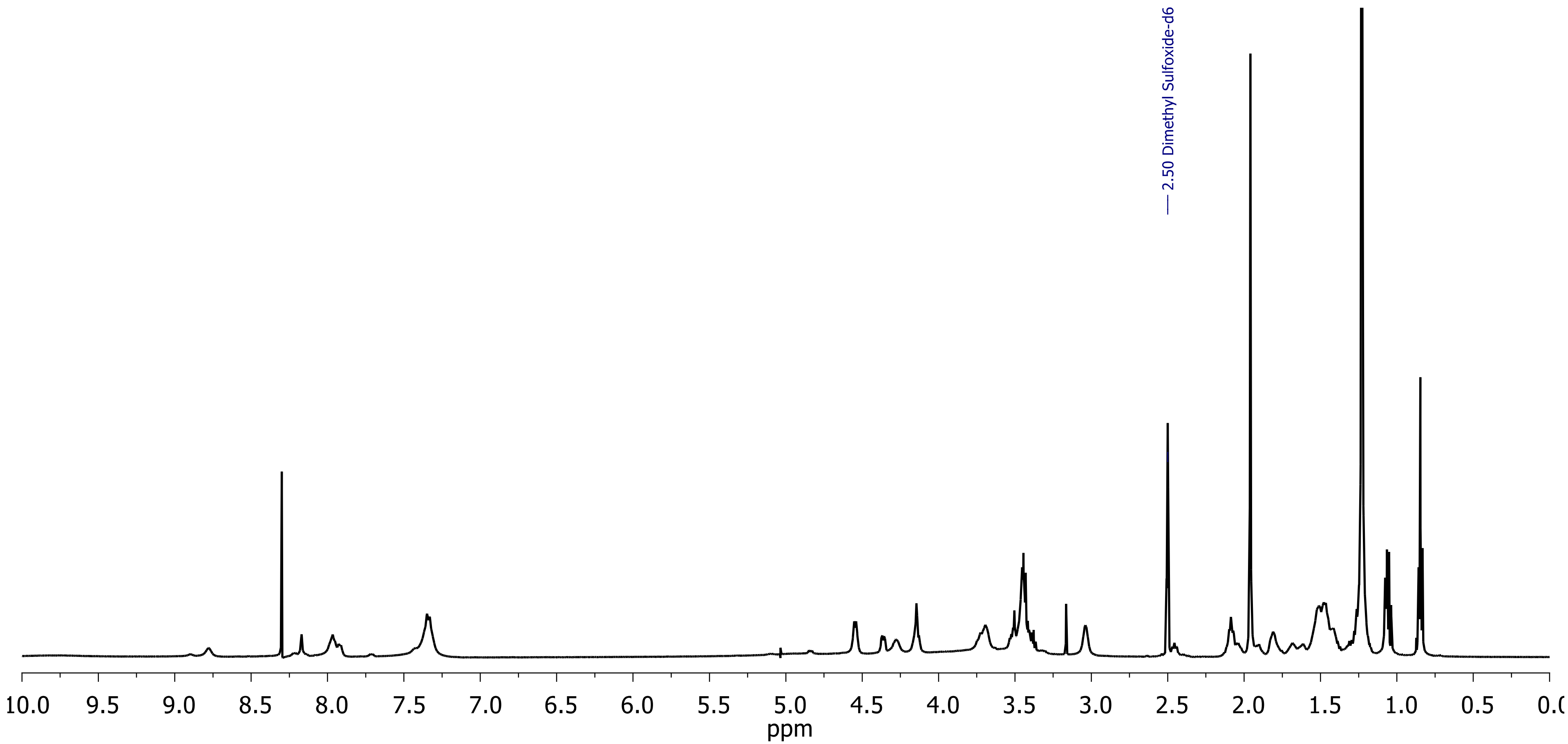

Figure S11. ^{1}H NMR spectrum of variochelin B (**2**) (500 MHz, DMSO-d_6).

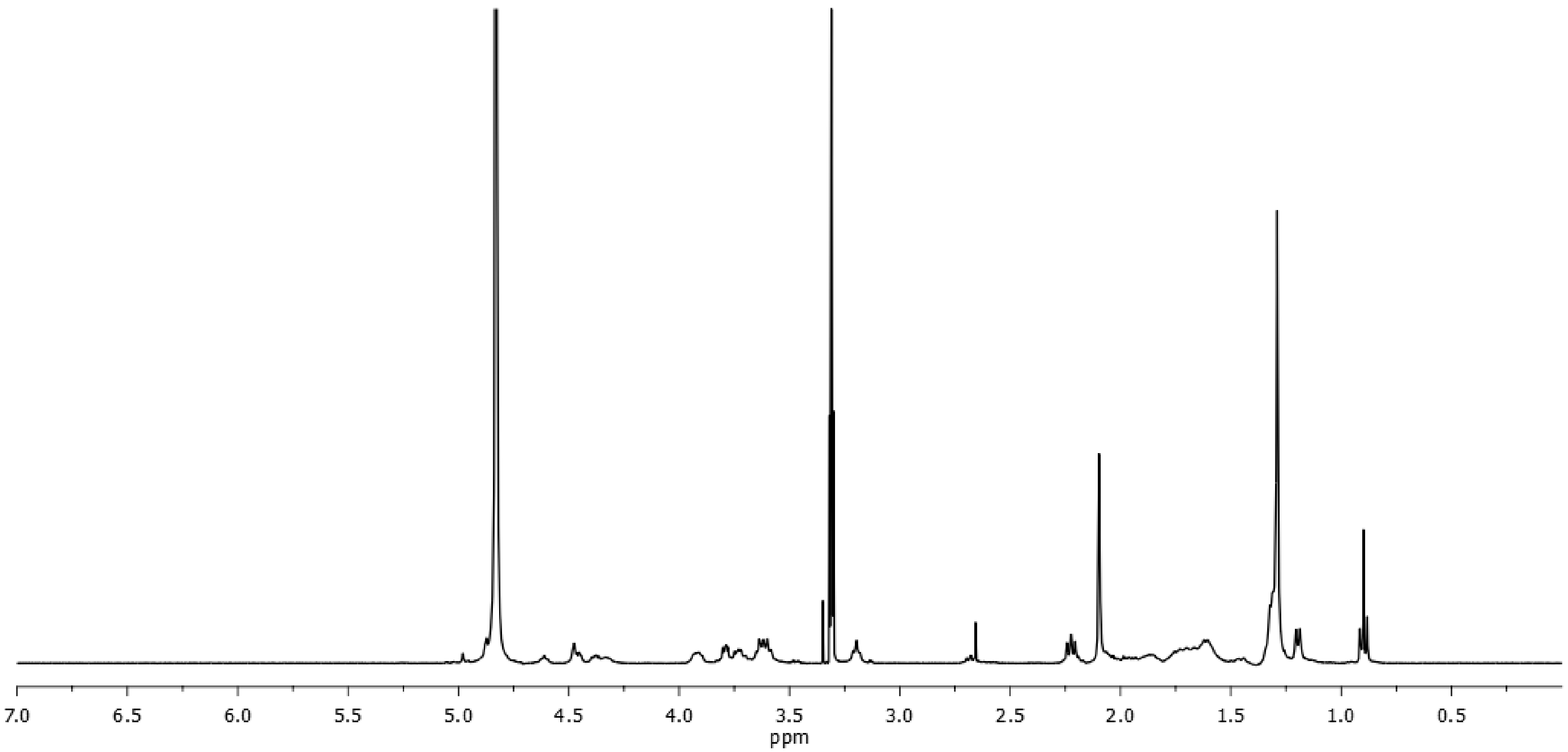

Figure S12. ^{1}H NMR spectrum of variochelin B (**2**). (500 MHz, methanol-d_4).

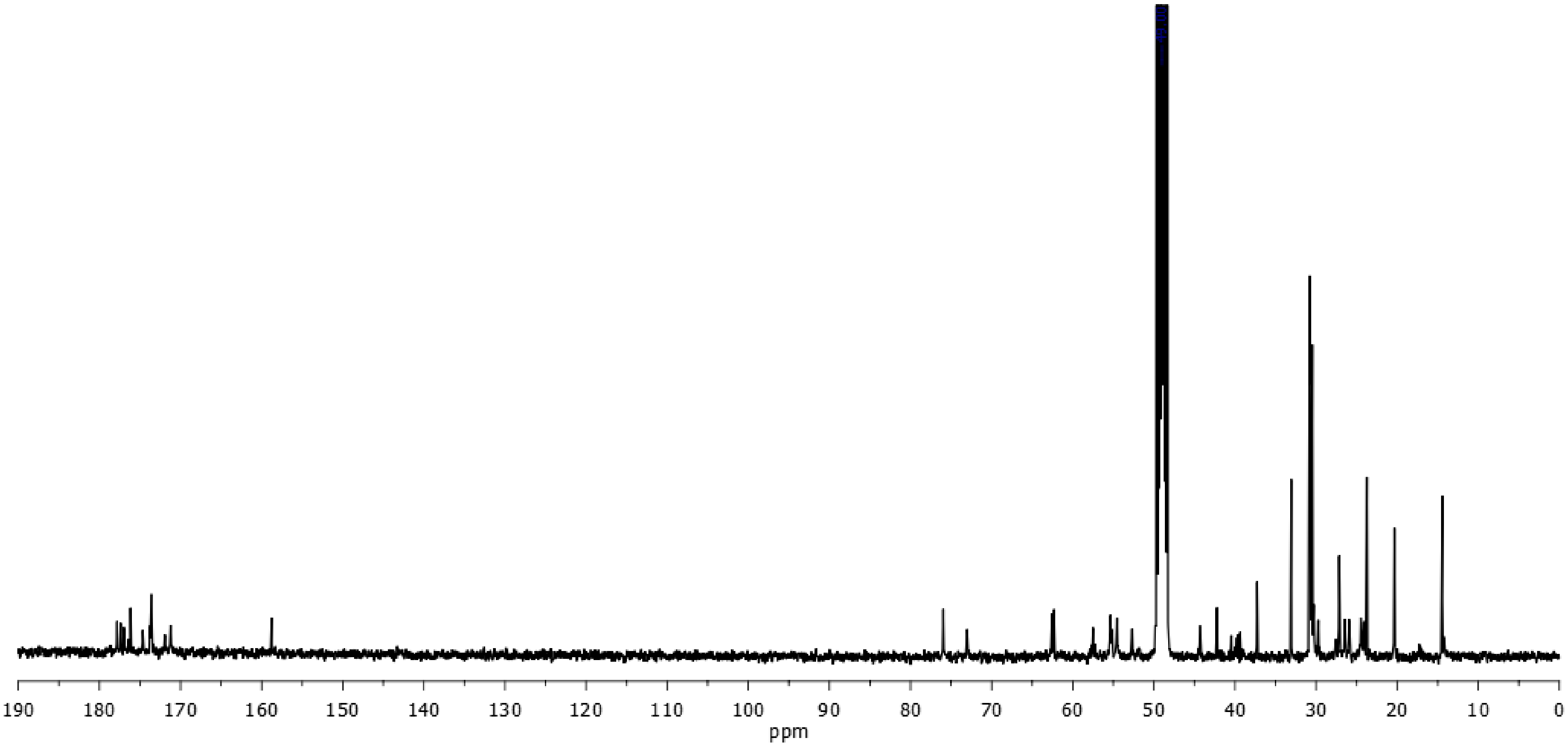

Figure S13. ^{13}C NMR spectrum of variochelin B (**2**). (125 MHz, methanol-d_4).

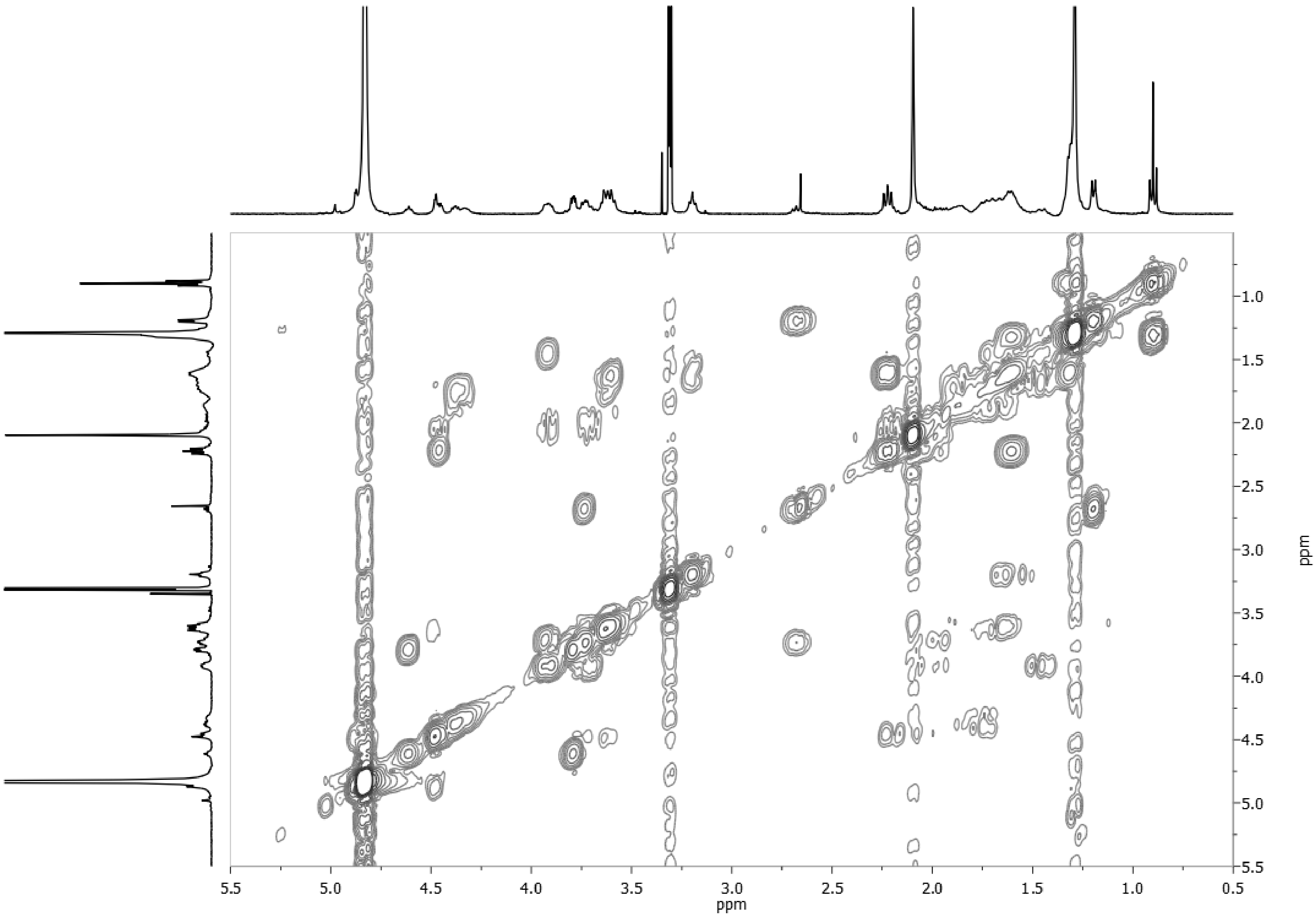

Figure S14. COSY spectrum of variochelin B (**2**). (500 MHz, methanol-d_4).

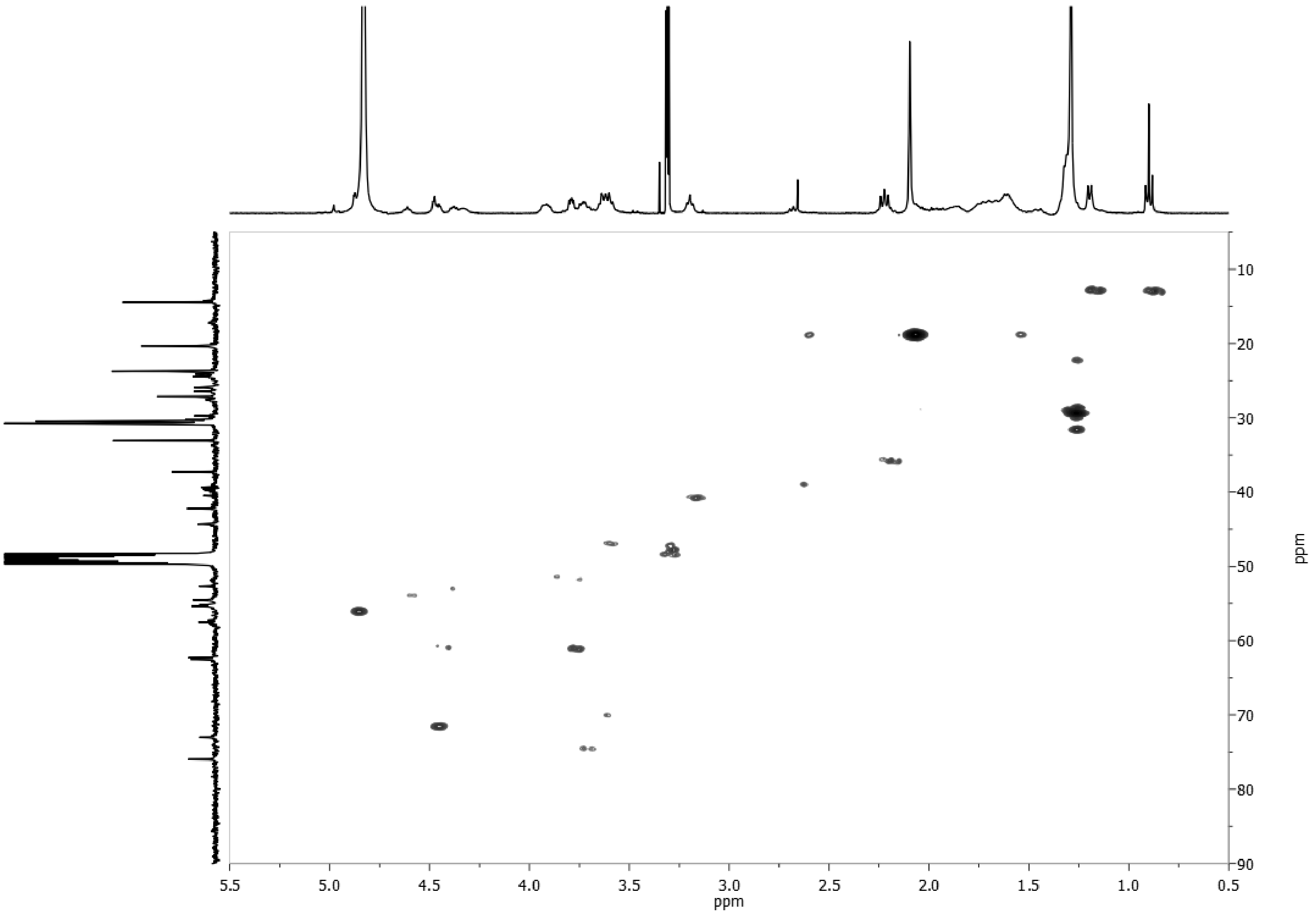

Figure S15. HSQC spectrum of variochelin B (**2**). (500 MHz, methanol-d_4).

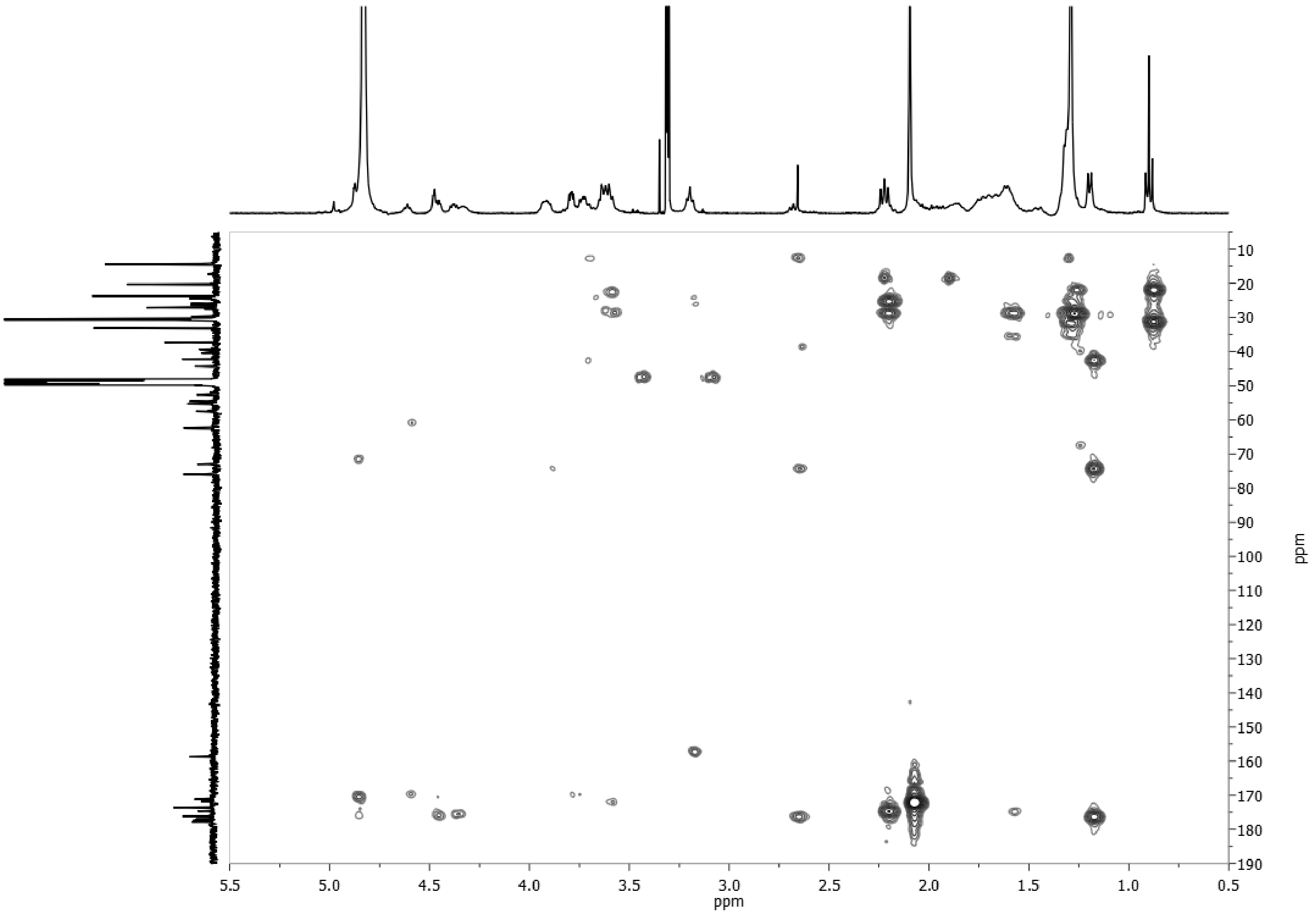

Figure S16. HMBC spectrum of variochelin B (**2**). (500 MHz, methanol-d_4).

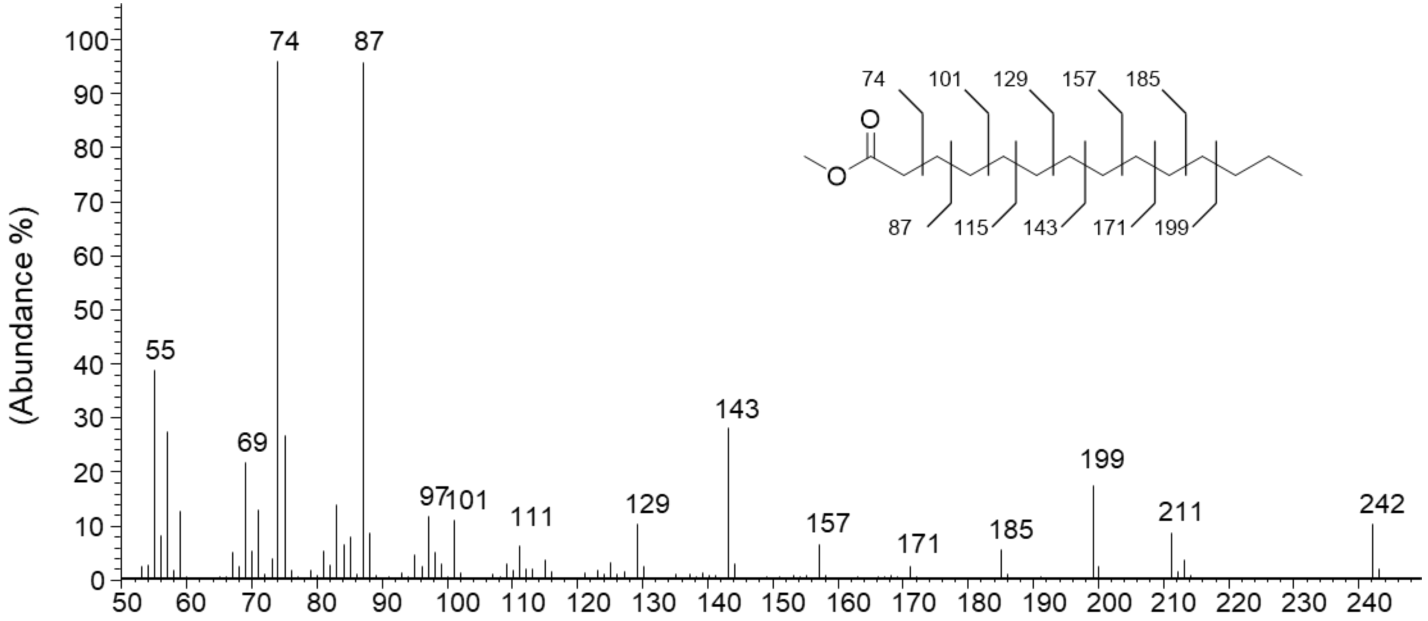

Figure S17. EIMS of tetradecanoic acid methyl ester in variochelin B (**2**).

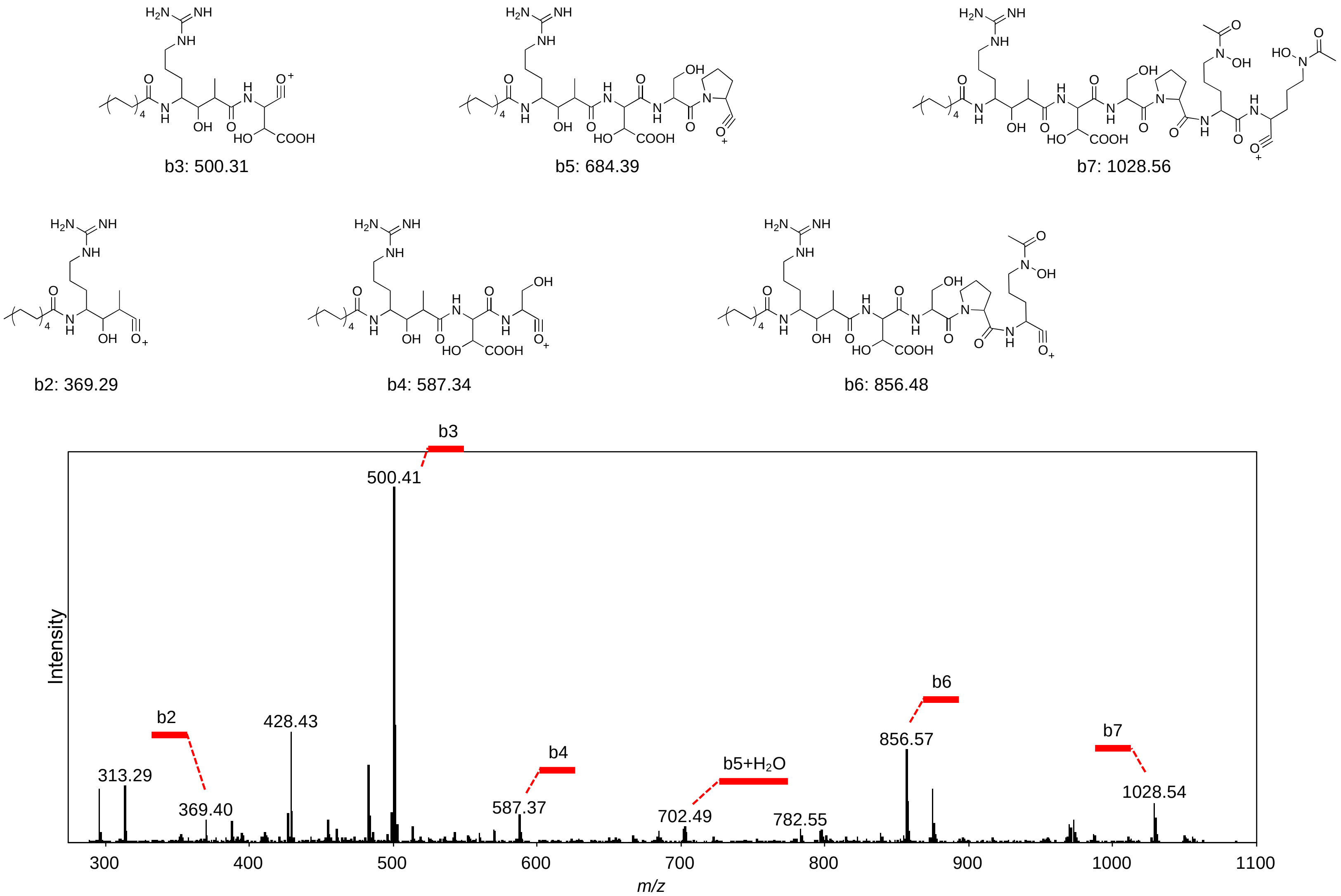

Figure S18. ESIMS/MS analysis of variochelin C (**3**). Structures and *m/z* of predicted fragmentation ions were described the top. An ion peak of $[M + H]^+$ at *m/z* 1047.06 was chosen as a parent ion.

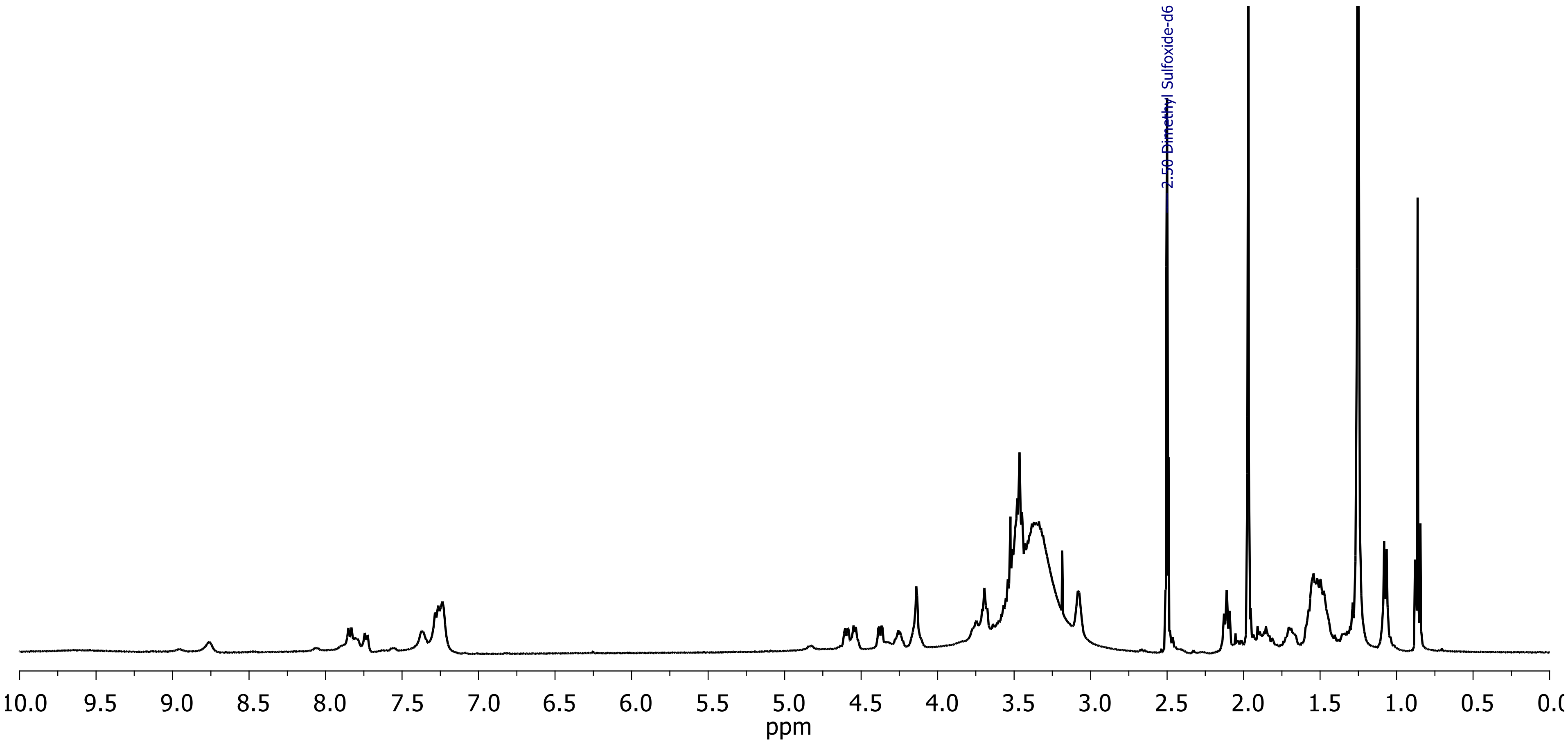

Figure S19. ^{1}H NMR spectrum of variochelin C (**3**) (500 MHz, DMSO-d_6).

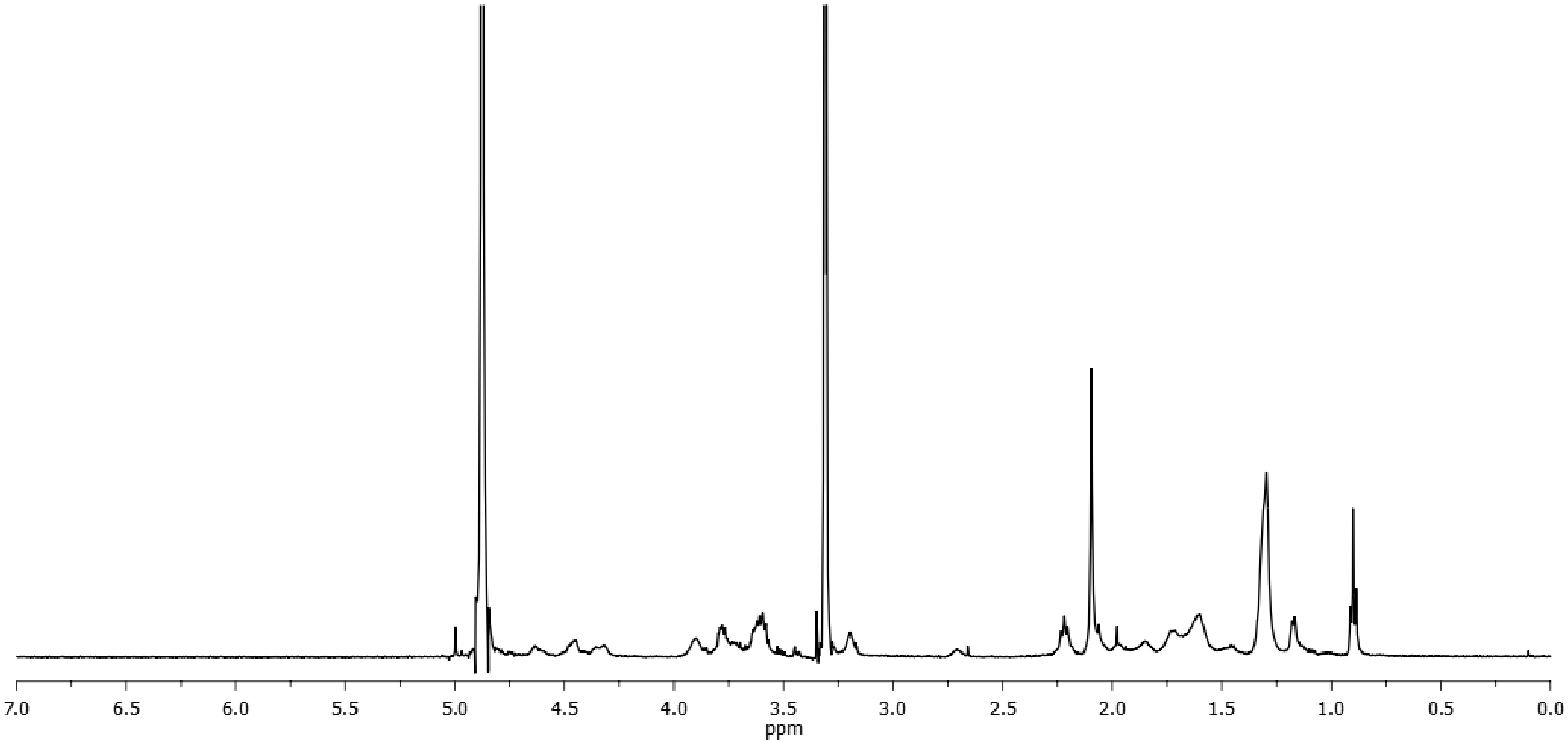

Figure S20. ^{1}H NMR spectrum of variochelin C (**3**). (500 MHz, methanol-d_4).

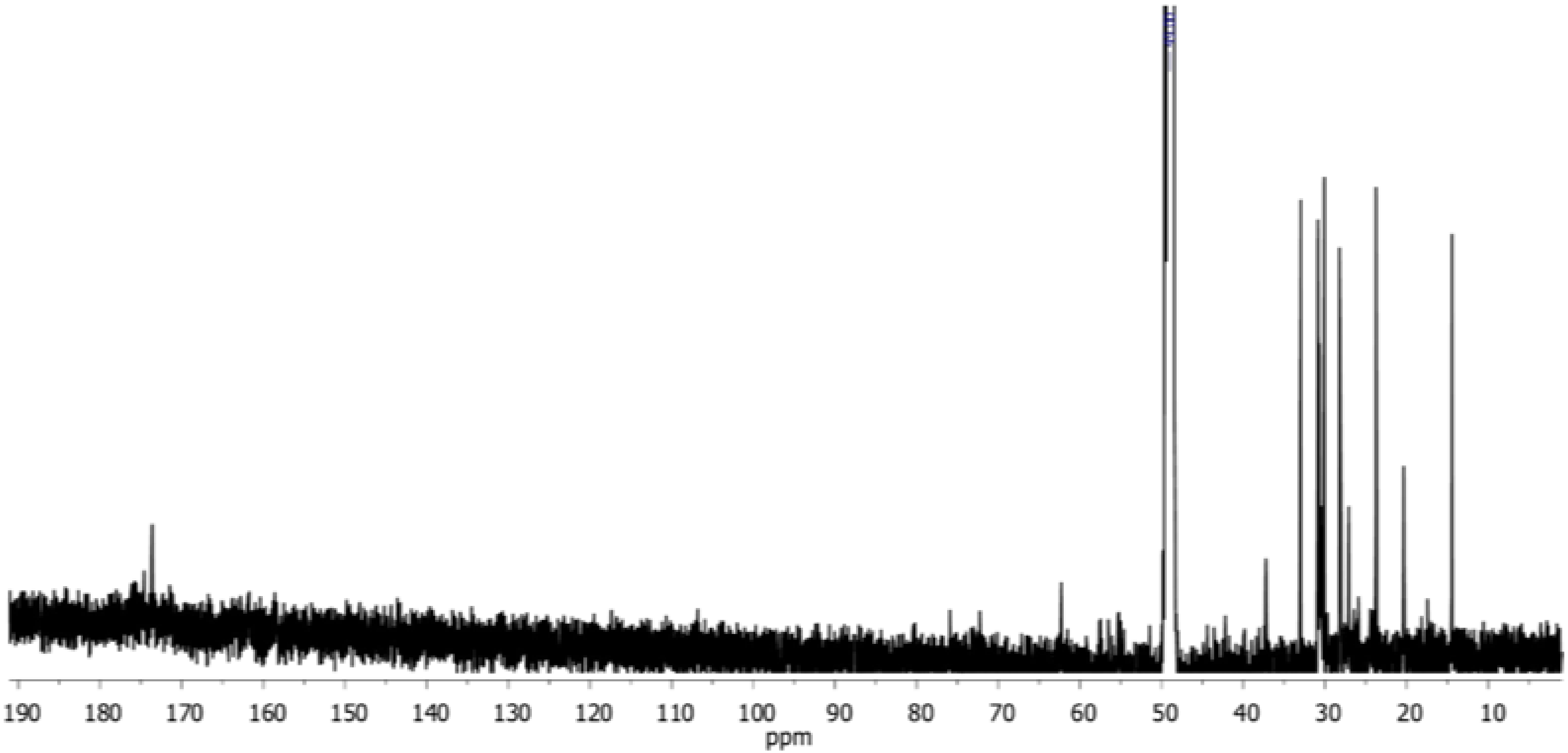

Figure S21. ^{13}C NMR spectrum of variochelin C (**3**). (125 MHz, methanol-d_4).

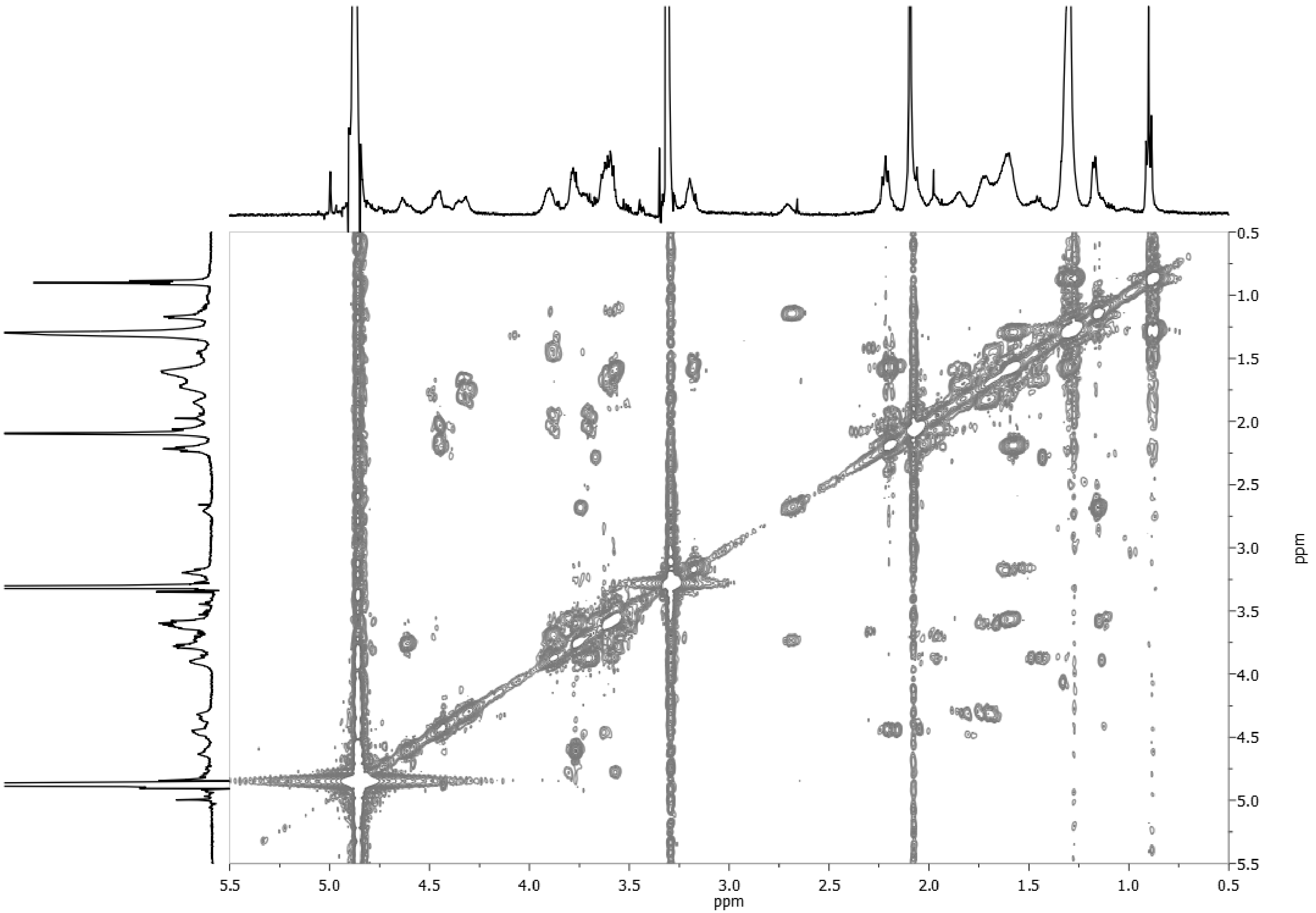

Figure S22. COSY spectrum of variochelin C (**3**). (500 MHz, methanol-d_4).

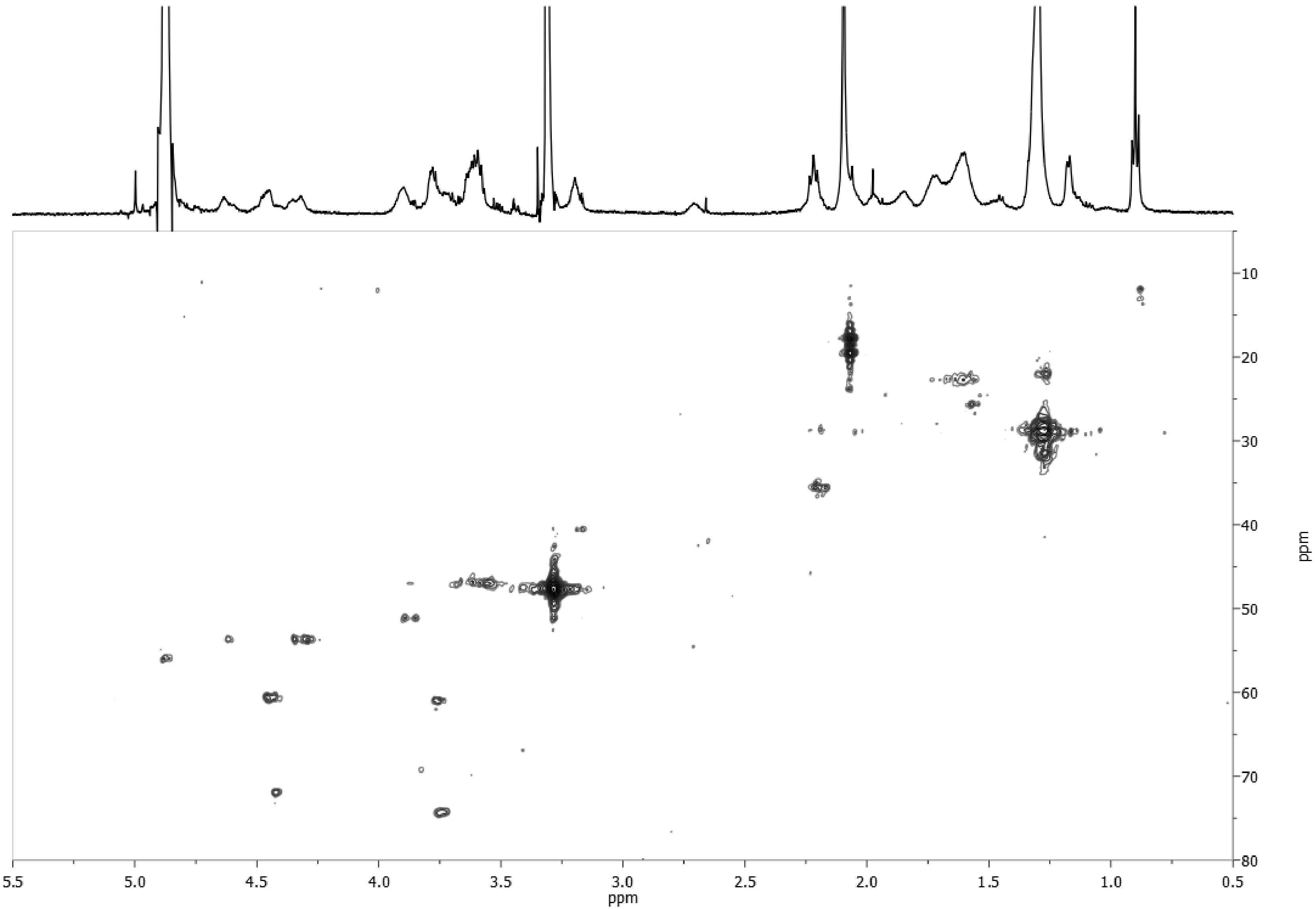

Figure S23. HSQC spectrum of variochelin C (**3**). (500 MHz, methanol-d_4).

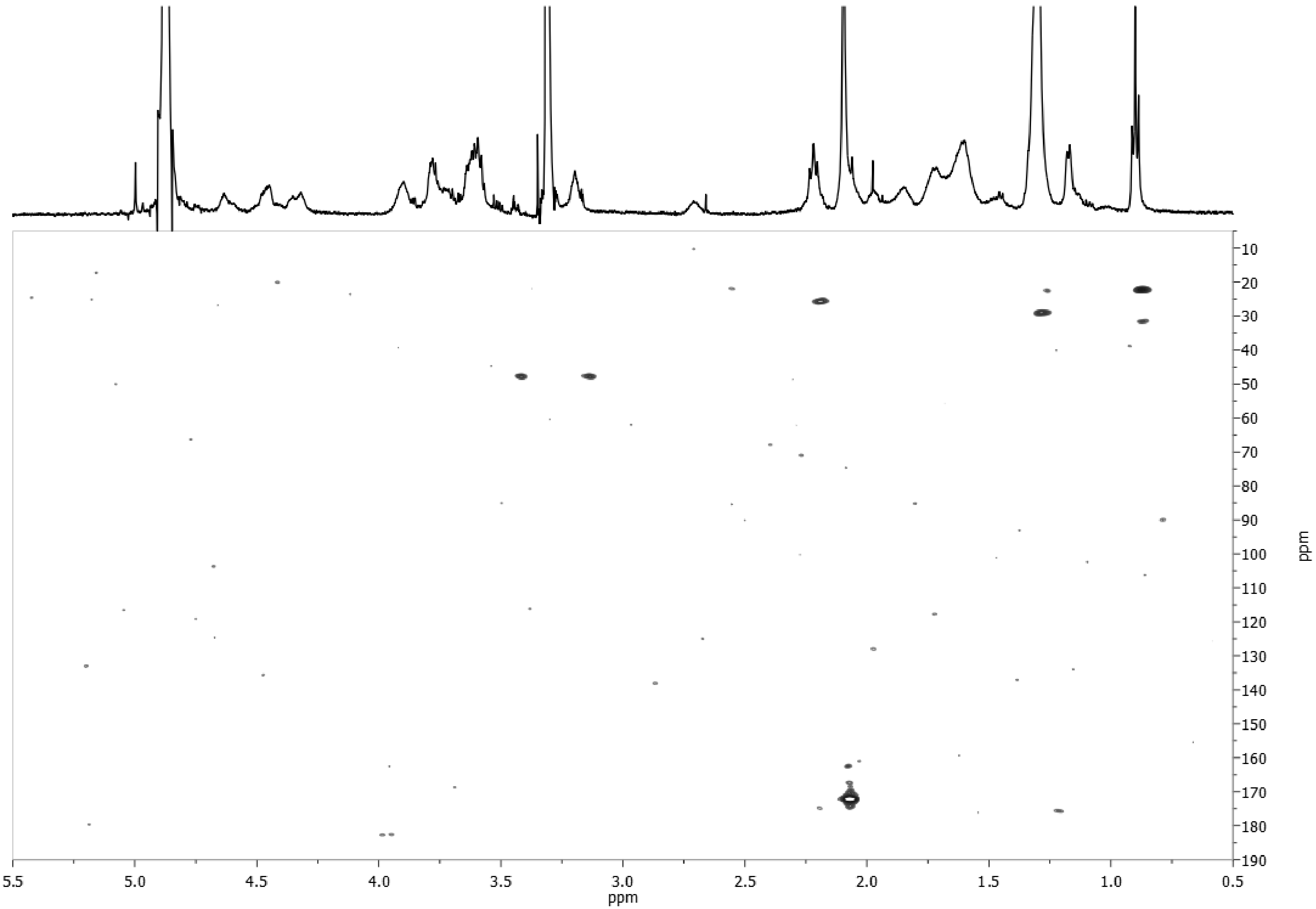

Figure S24. HMBC spectrum of variochelin C (**3**). (500 MHz, methanol-d_4).

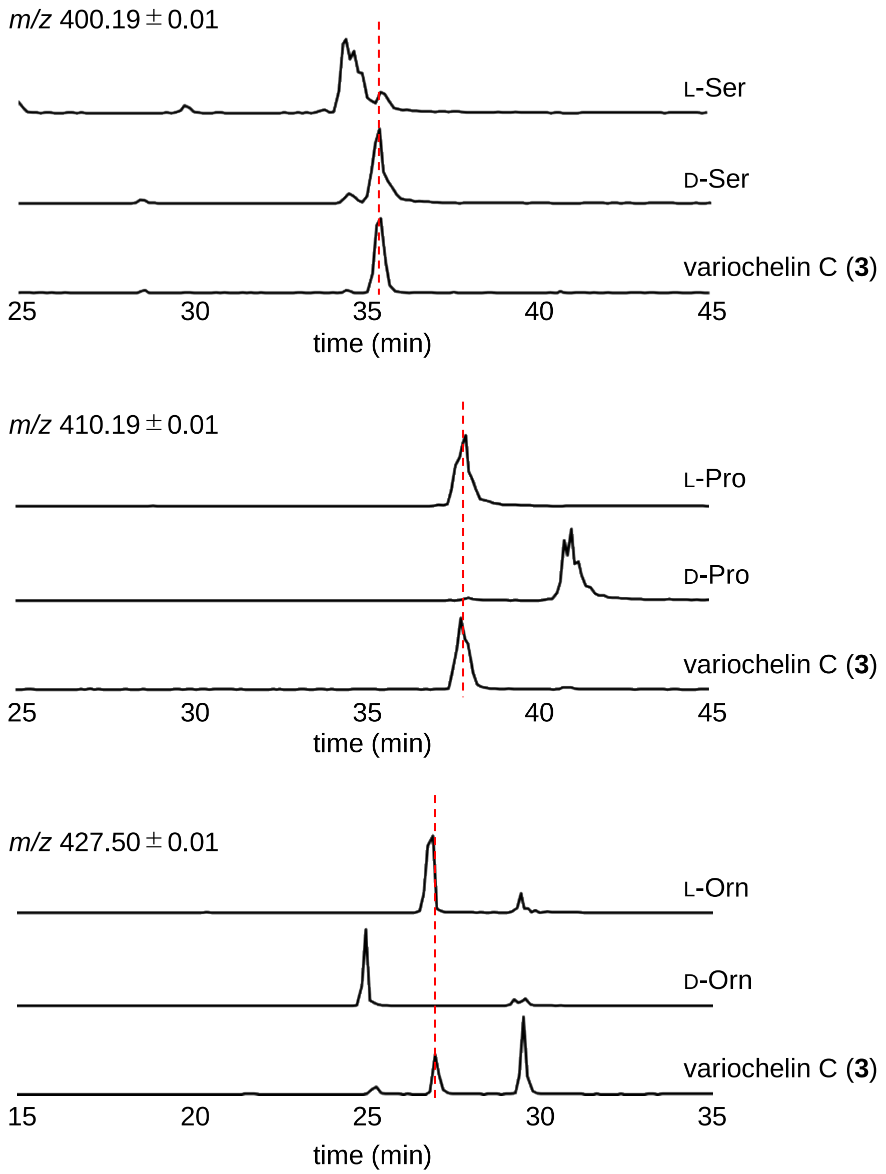

Figure S25. Extracted ion chromatograms from advanced Marfey's analyses of variochelin C (**3**).

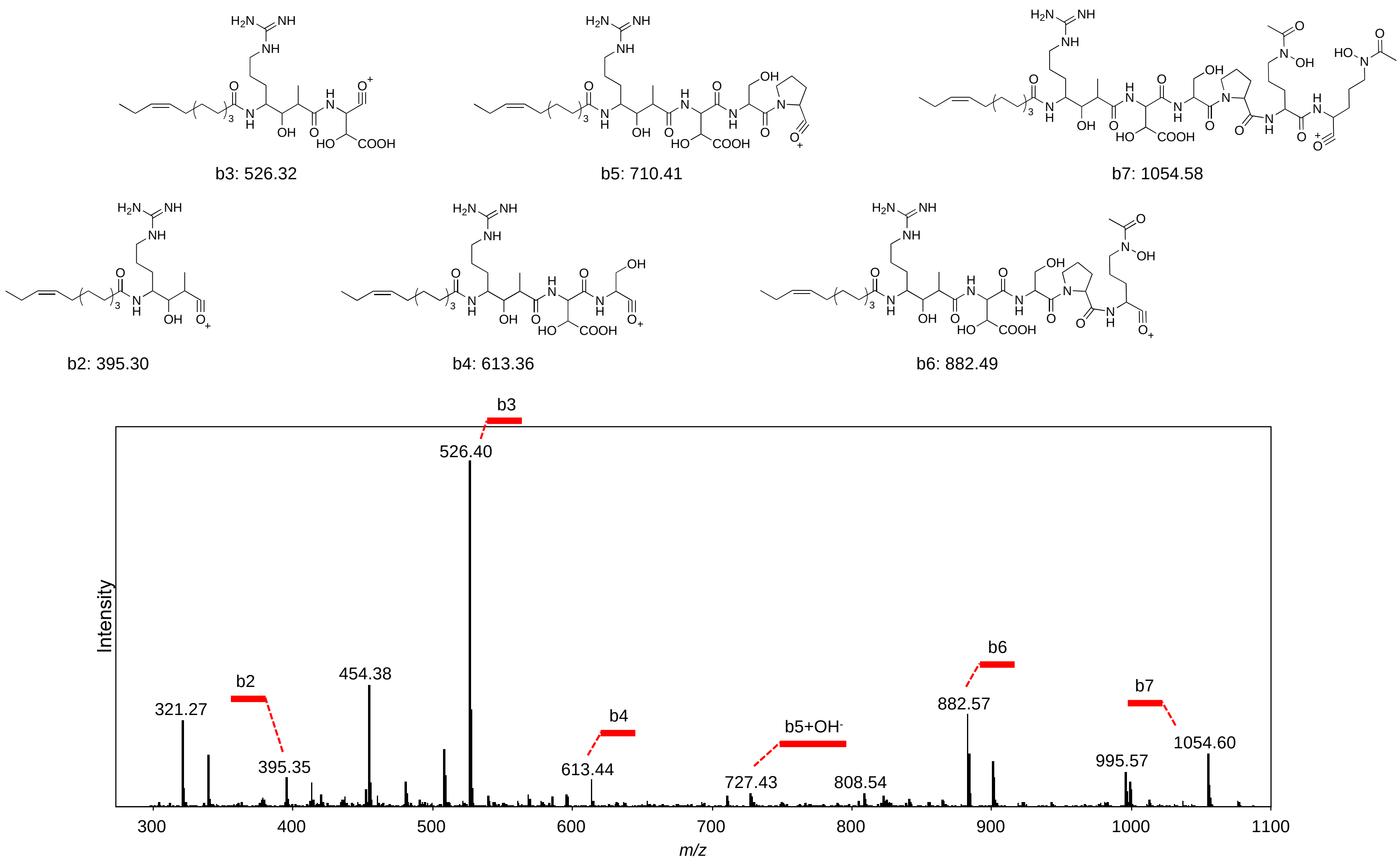

Figure S26. ESIMS/MS analysis of variochelin D (**4**). Structures and *m/z* of predicted fragmentation ions were described the top. An ion peak of $[M + H]^+$ at *m/z* 1072.95 was chosen as a parent ion.

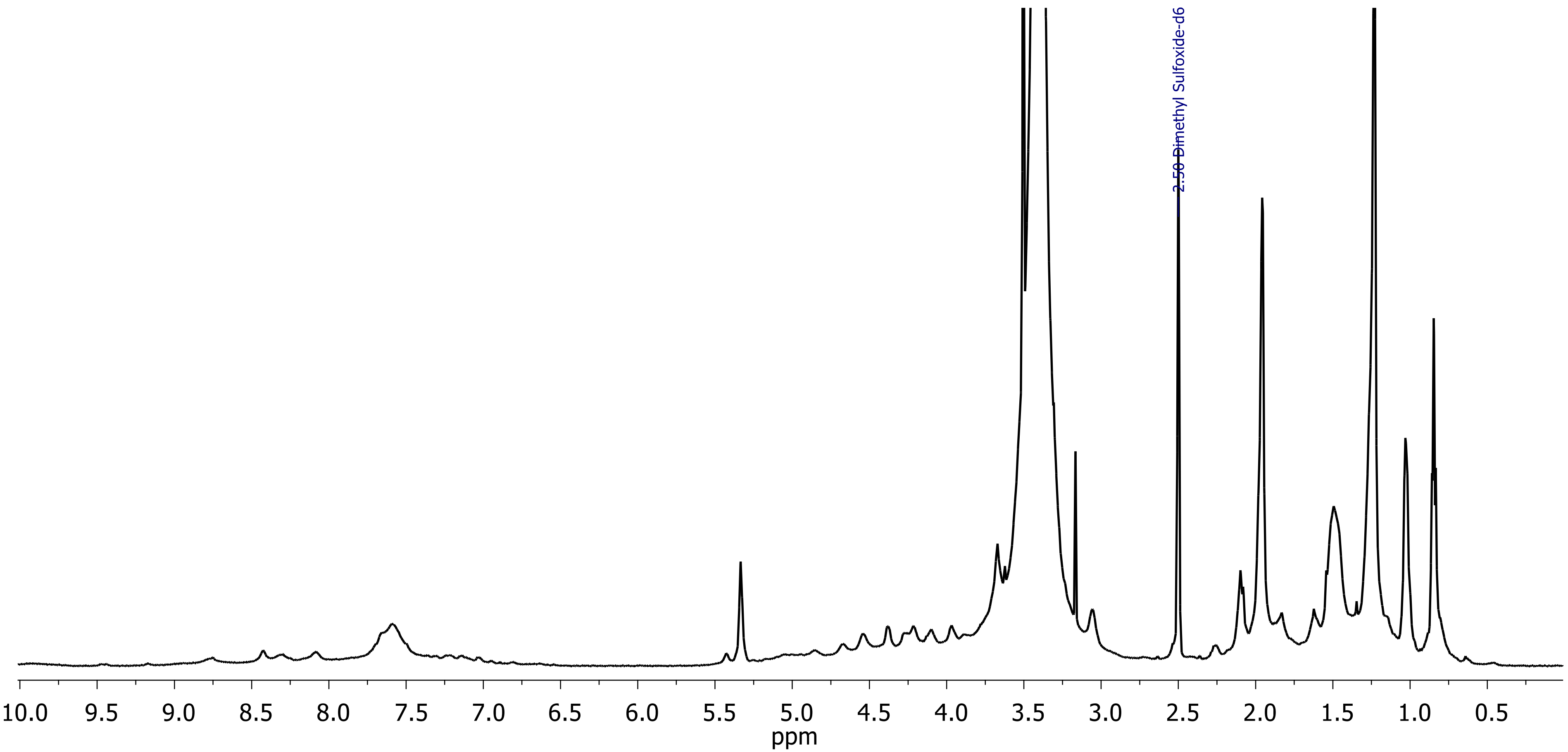

Figure S27. ^{1}H NMR spectrum of variochelin D (**4**) (500 MHz, DMSO-d_6).

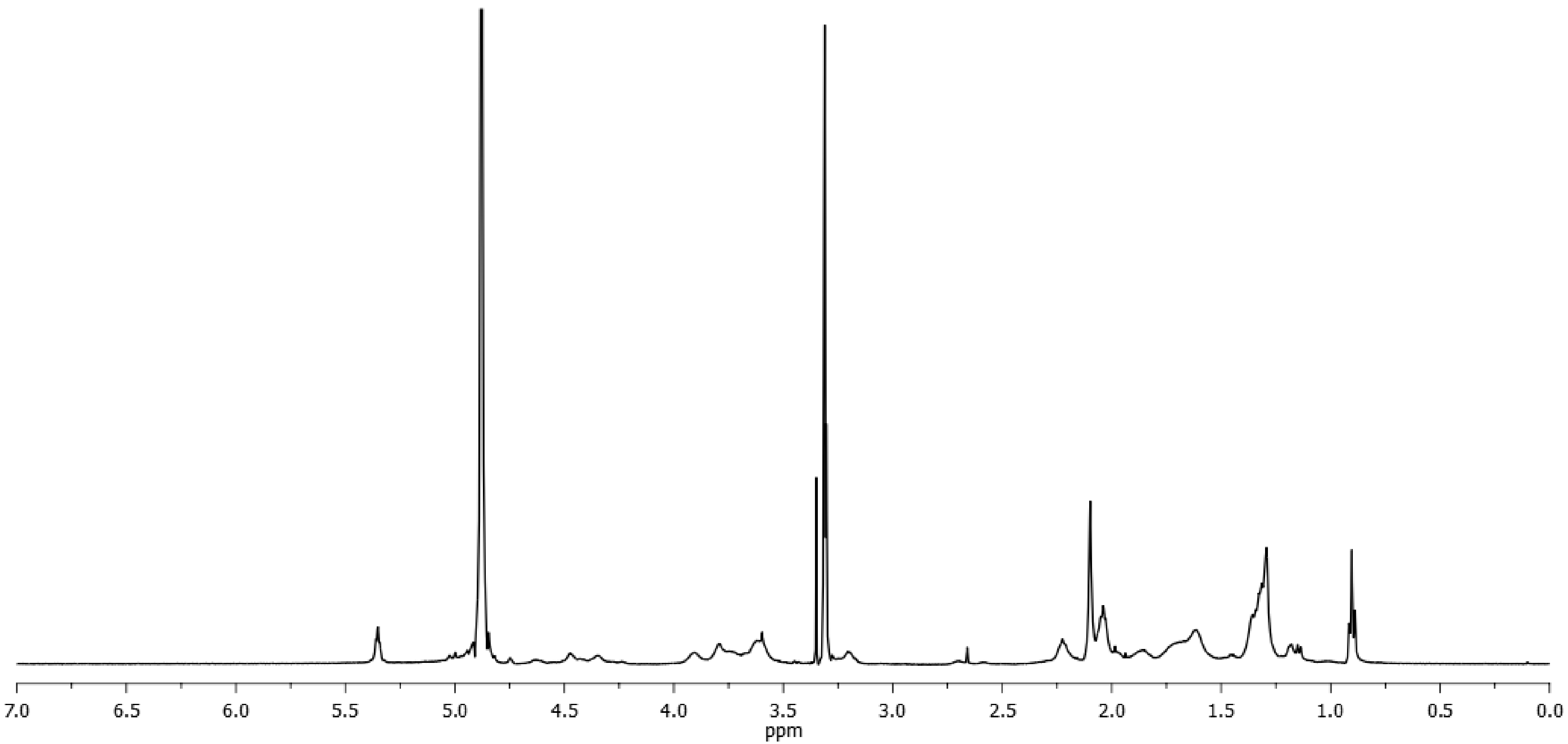

Figure S28. ^{1}H NMR spectrum of variochelin D (**4**). (500 MHz, methanol-d_4).

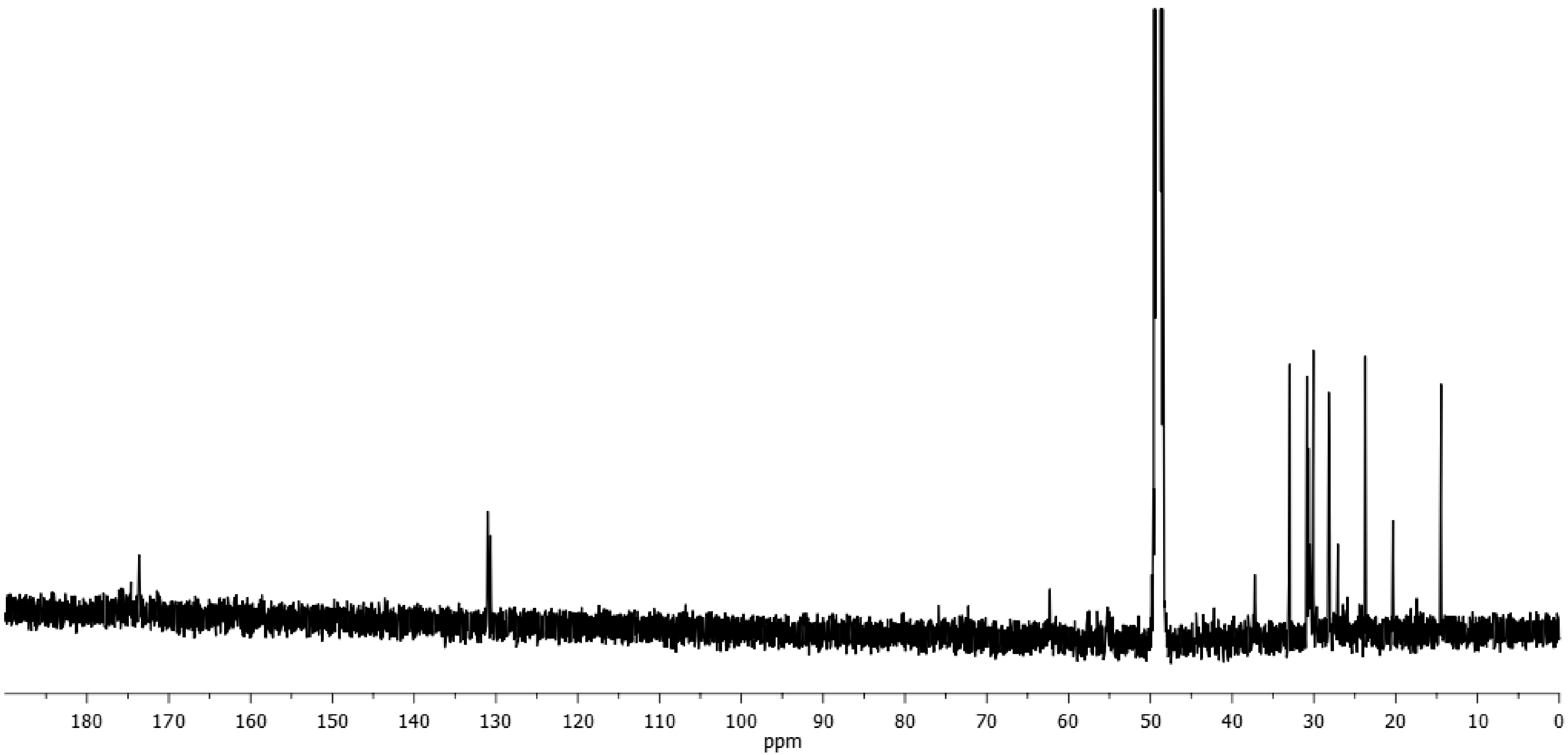

Figure S29. ^{13}C NMR spectrum of variochelin D (**4**). (125 MHz, methanol-d_4).

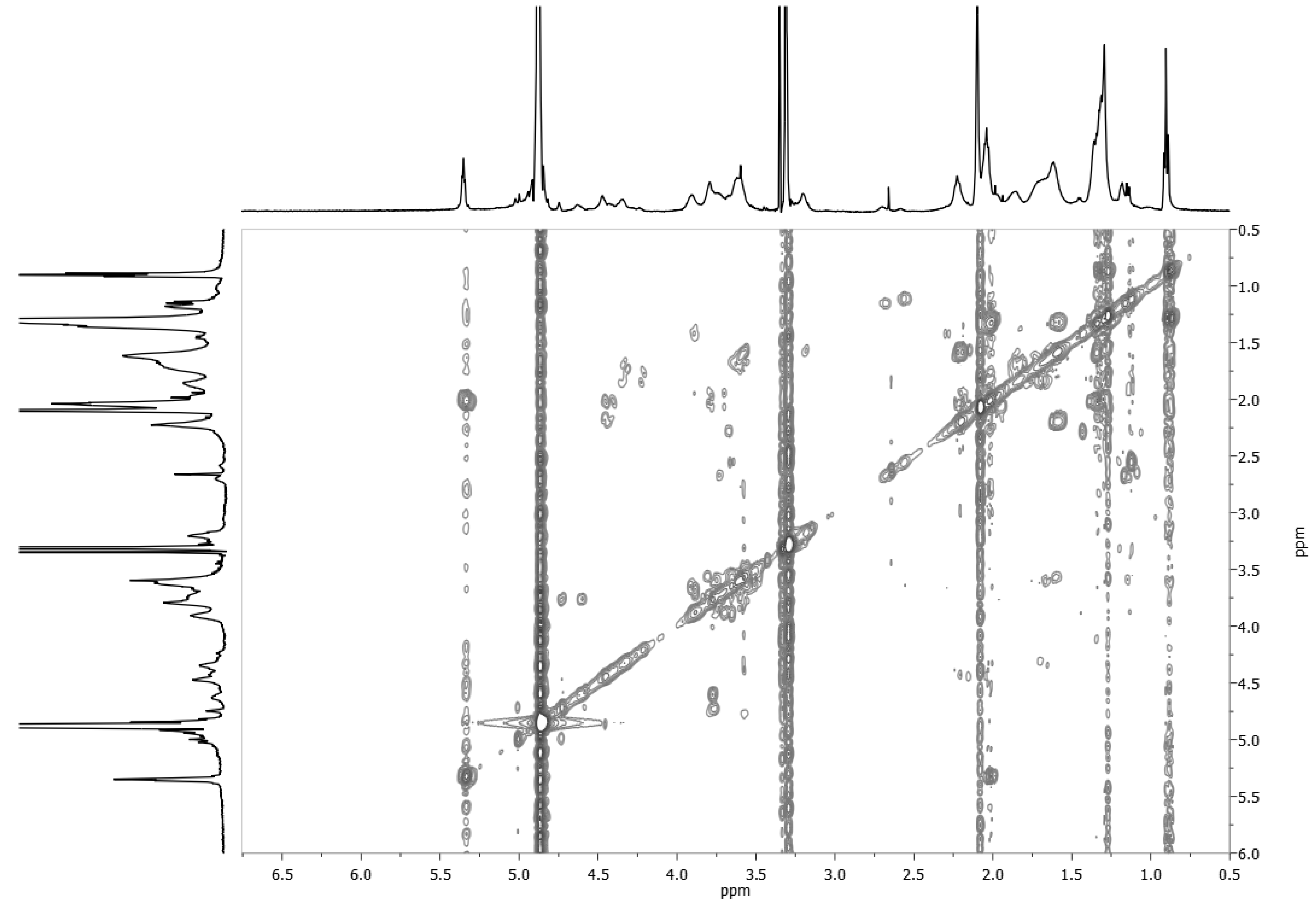

Figure S30. COSY spectrum of variochelin D (**4**). (500 MHz, methanol-d_4).

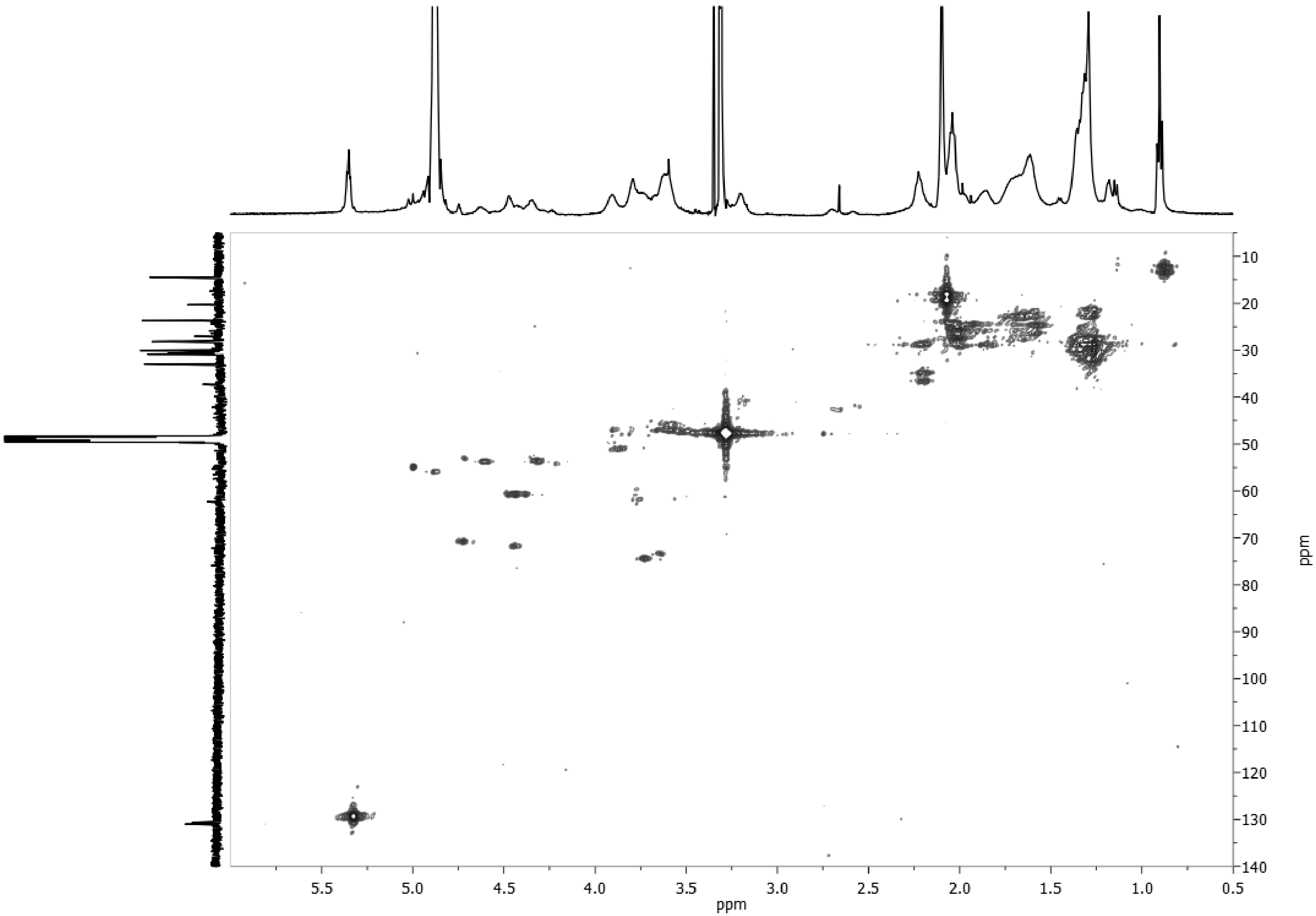

Figure S31. HSQC spectrum of variochelin D (**4**). (500 MHz, methanol-d_4).

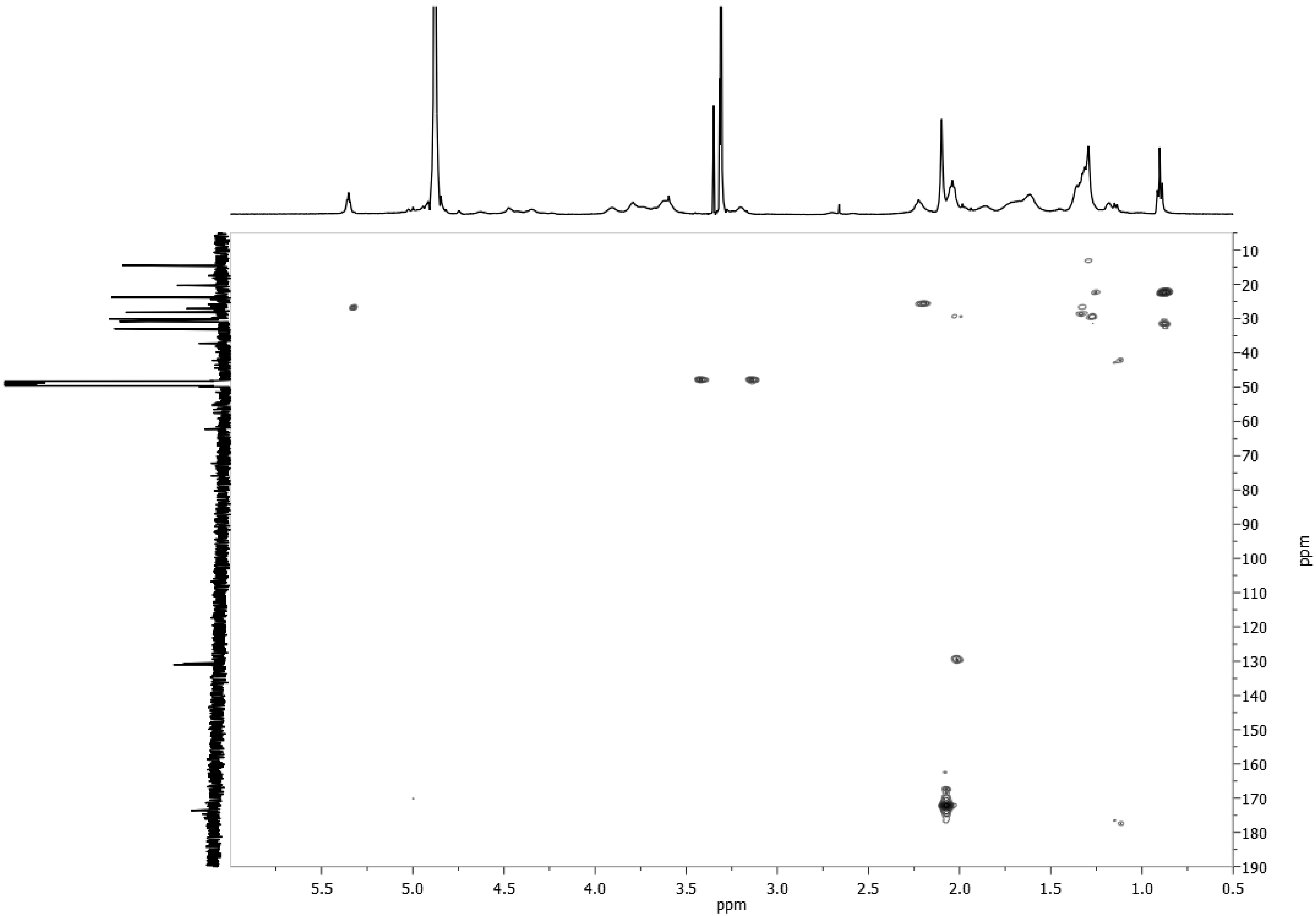

Figure S32. HMBC spectrum of variochelin D (**4**). (500 MHz, methanol-d_4).

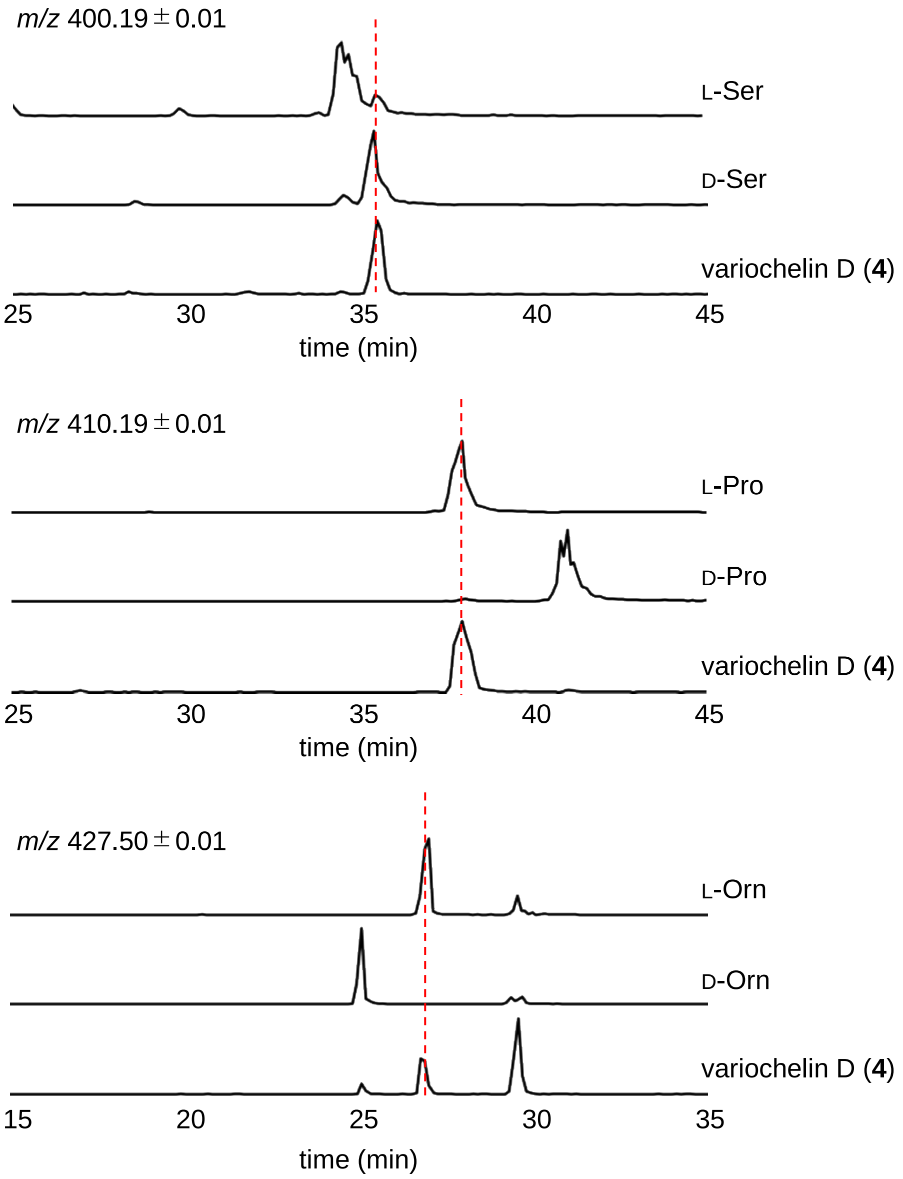

Figure S33. Extracted ion chromatograms from advanced Marfey's analyses of variochelin D (**4**).

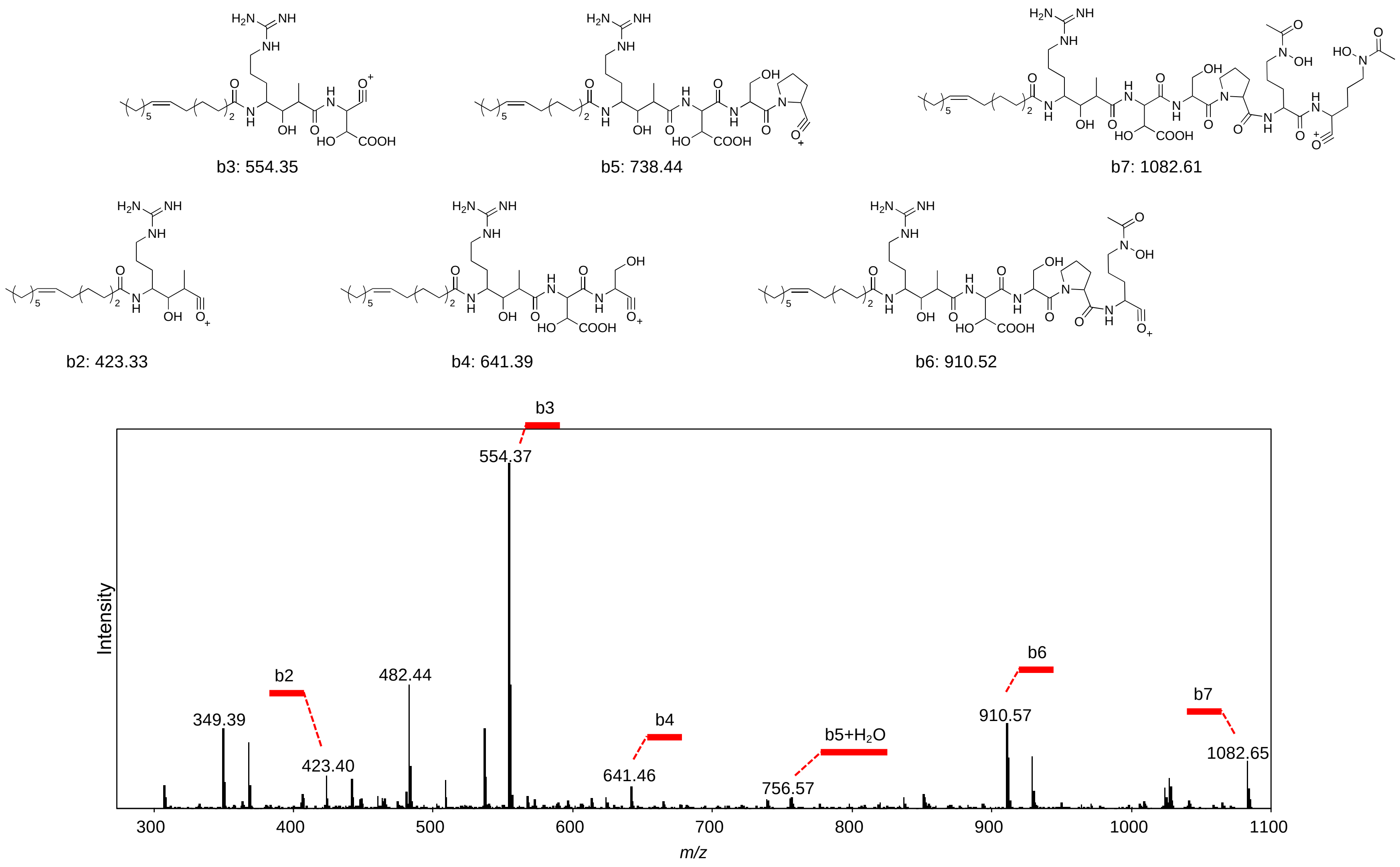

Figure S34. ESIMS/MS analysis of variochelin E (**5**). Structures and *m/z* of predicted fragmentation ions were described the top. An ion peak of $[M + H]^+$ at *m/z* 1101.14 was chosen as a parent ion.

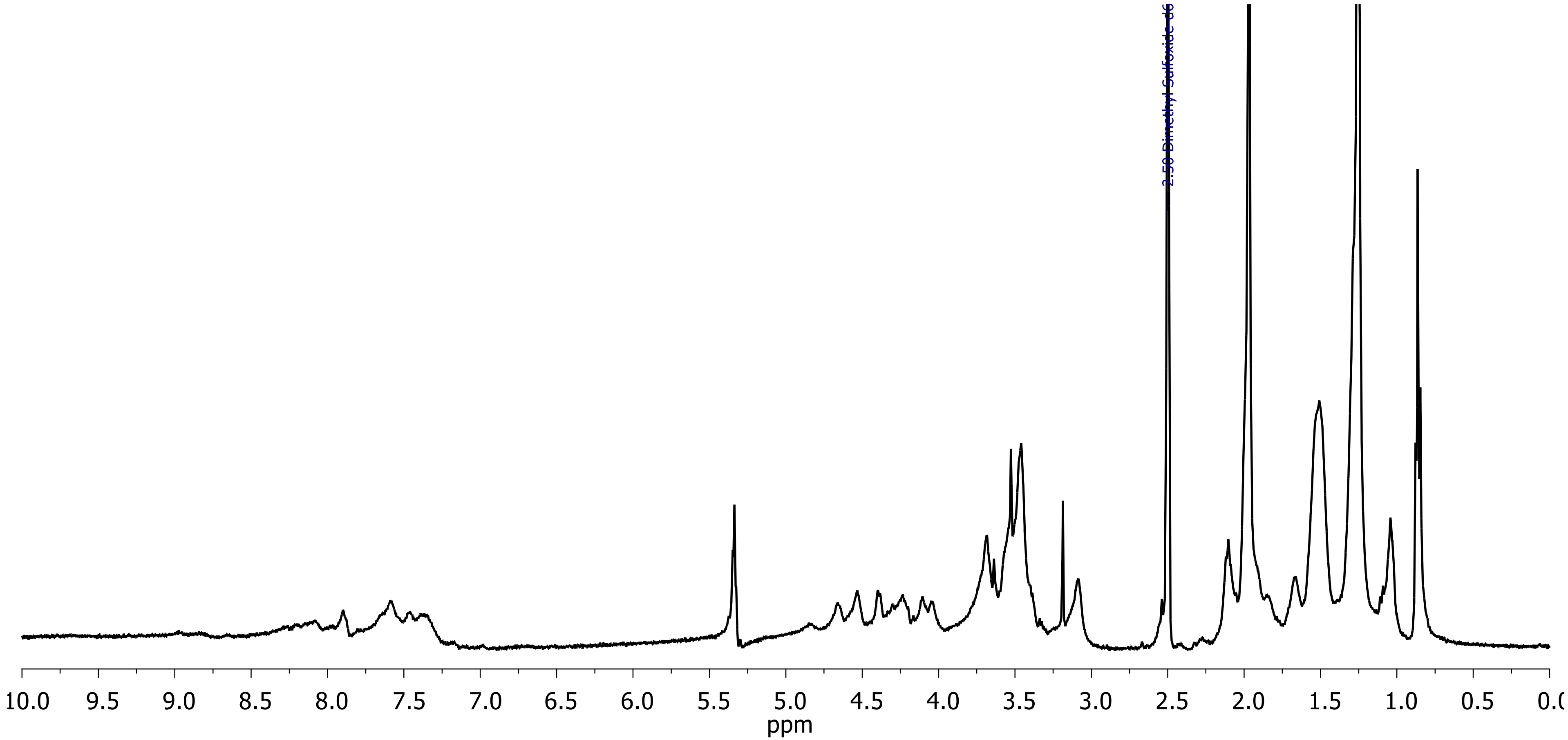

Figure S35. ^{1}H NMR spectrum of variochelin E (**5**) (500 MHz, DMSO-d_6).

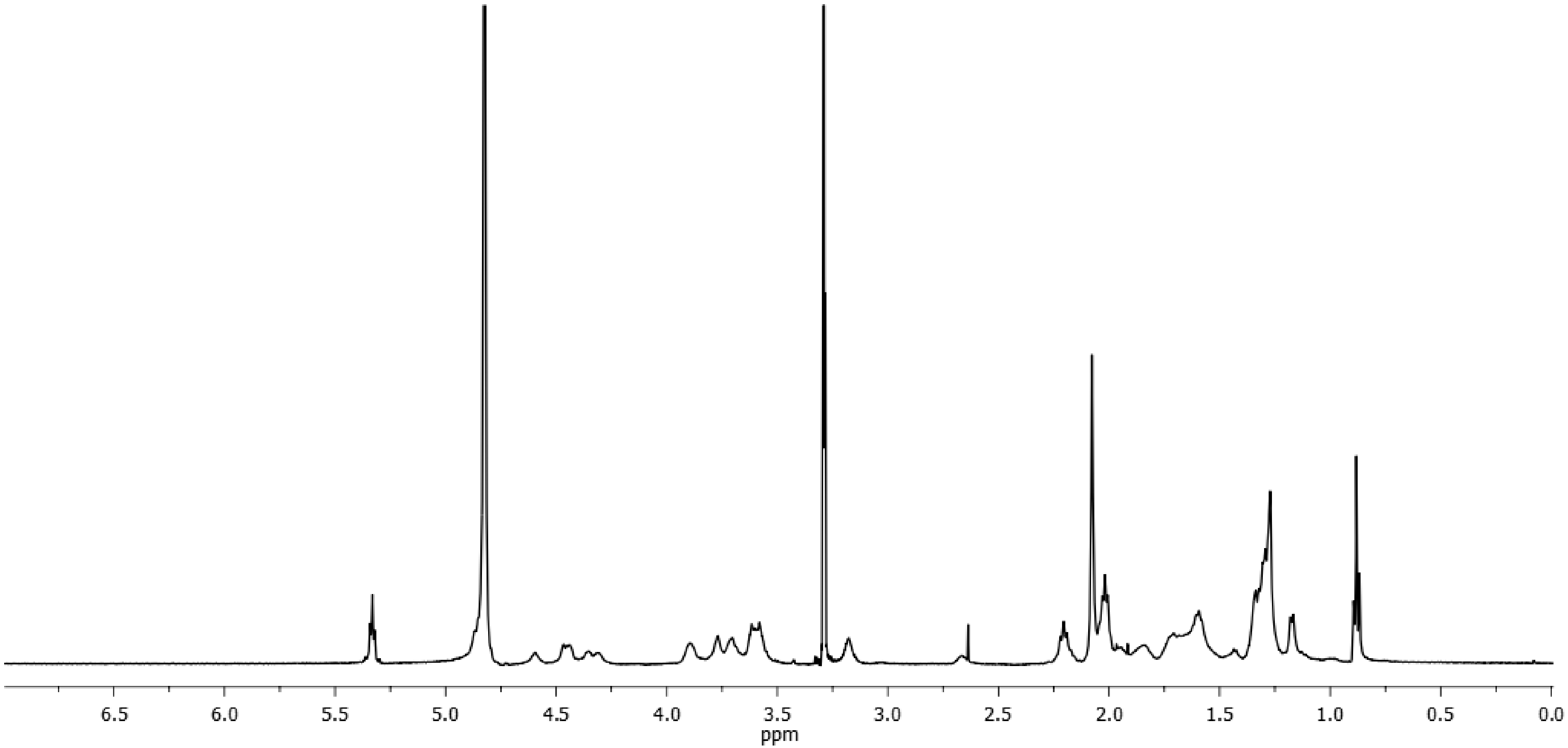

Figure S36. ^{1}H NMR spectrum of variochelin E (**5**). (500 MHz, methanol-d_4).

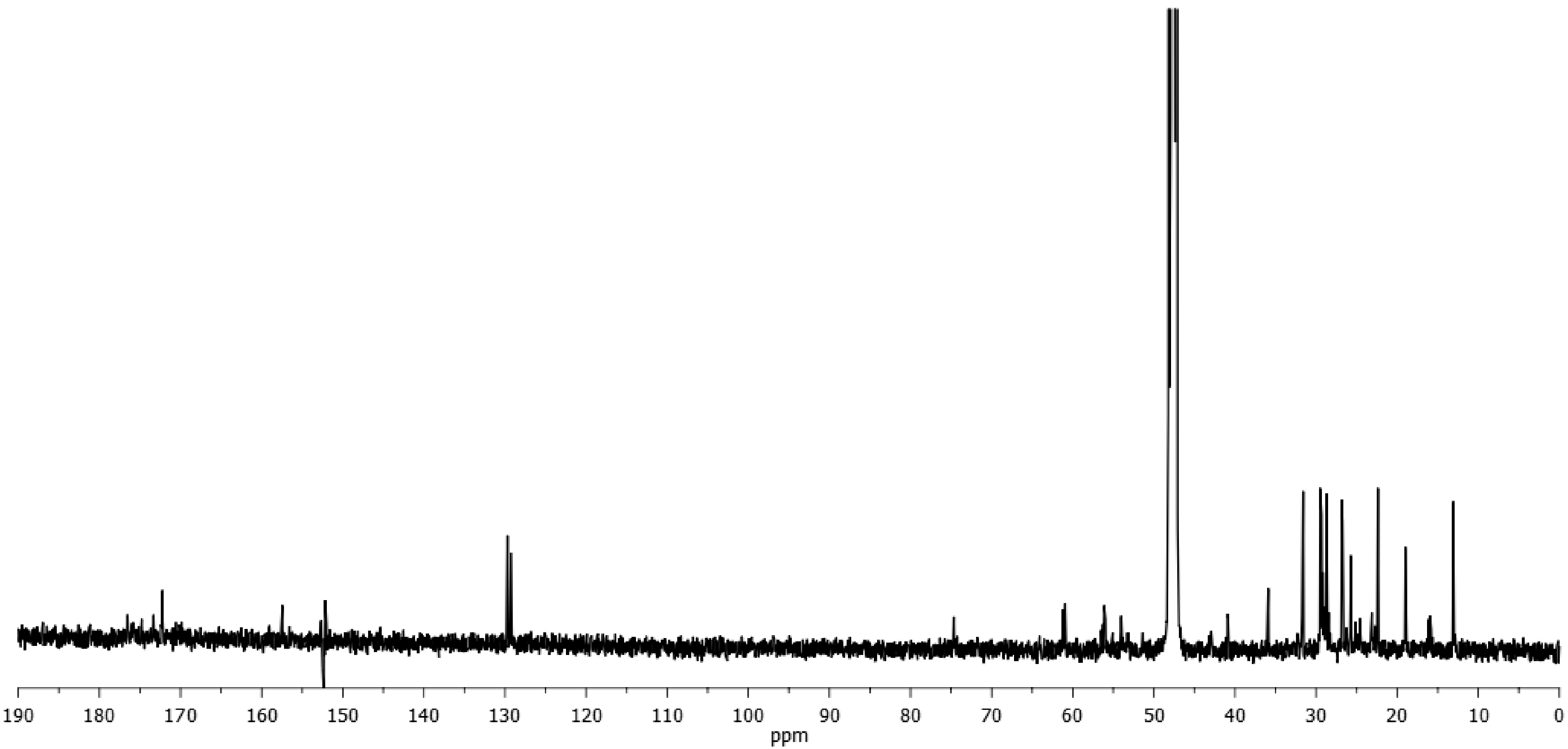

Figure S37. ^{13}C NMR spectrum of variochelin E (**5**). (125 MHz, methanol-d_4).

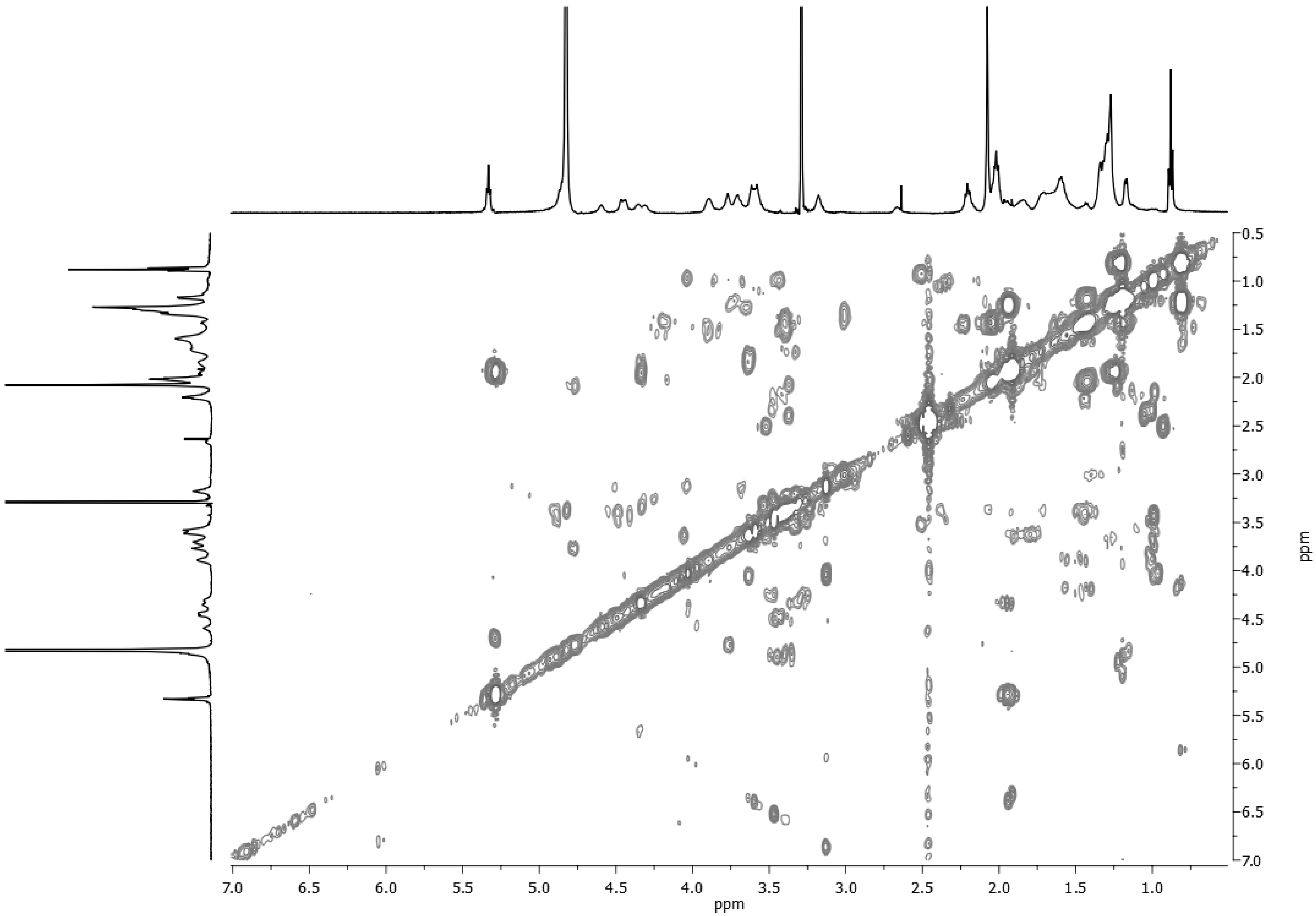

Figure S38. COSY spectrum of variochelin E (**5**). (500 MHz, methanol-d_4).

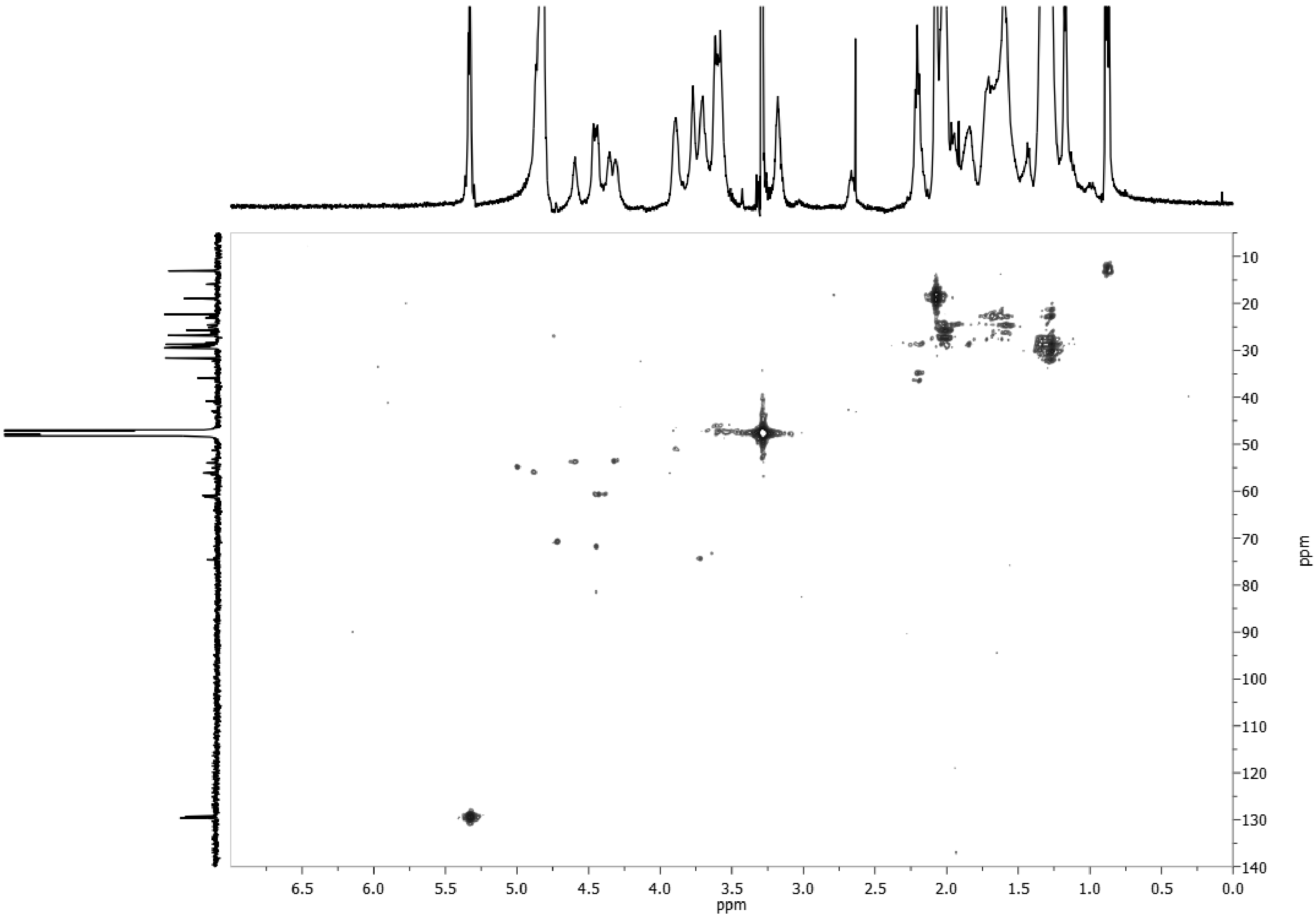

Figure S39. HSQC spectrum of variochelin E (**5**). (500 MHz, methanol-d_4).

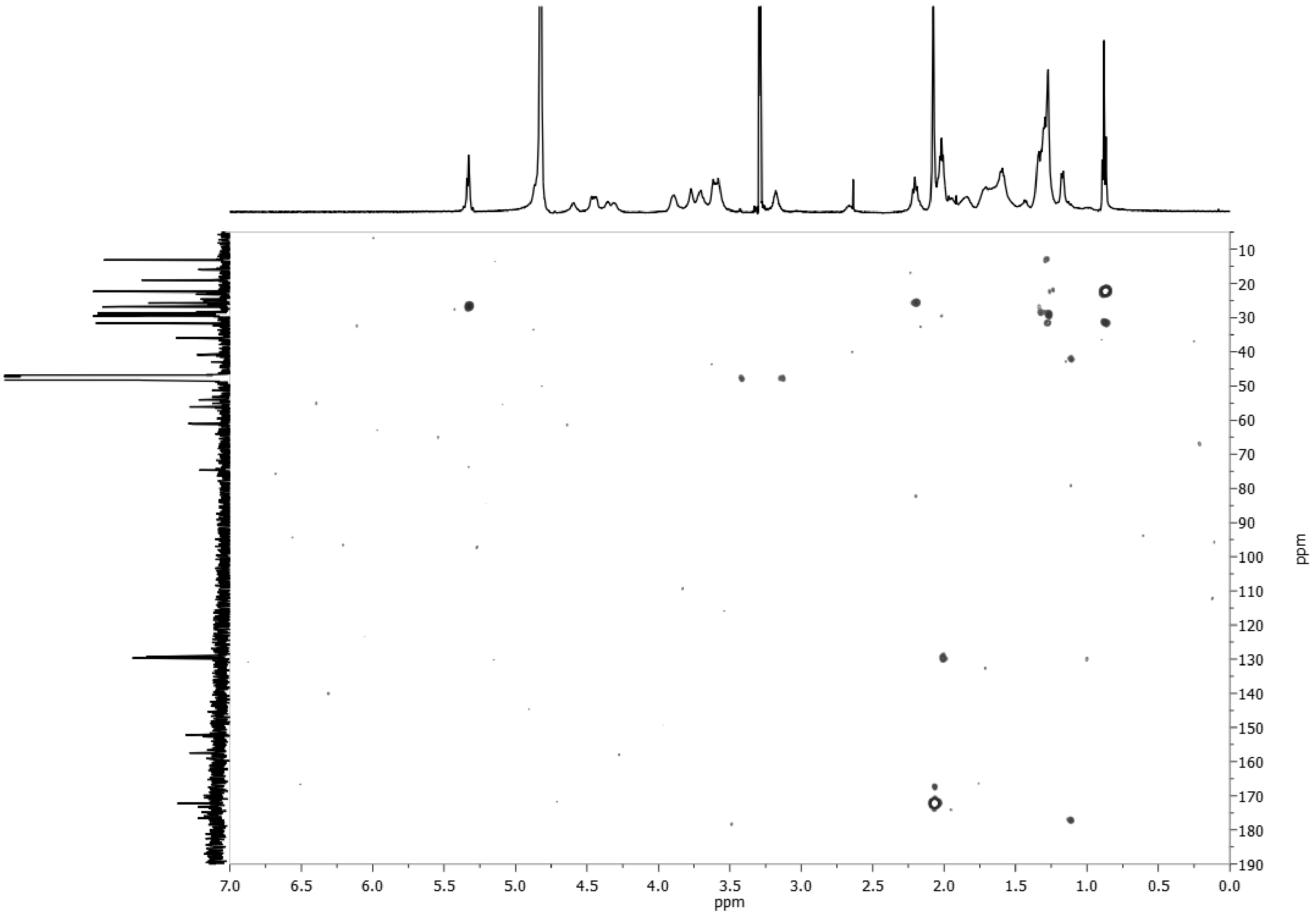

Figure S40. HMBC spectrum of variochelin E (**5**). (500 MHz, methanol-d_4).

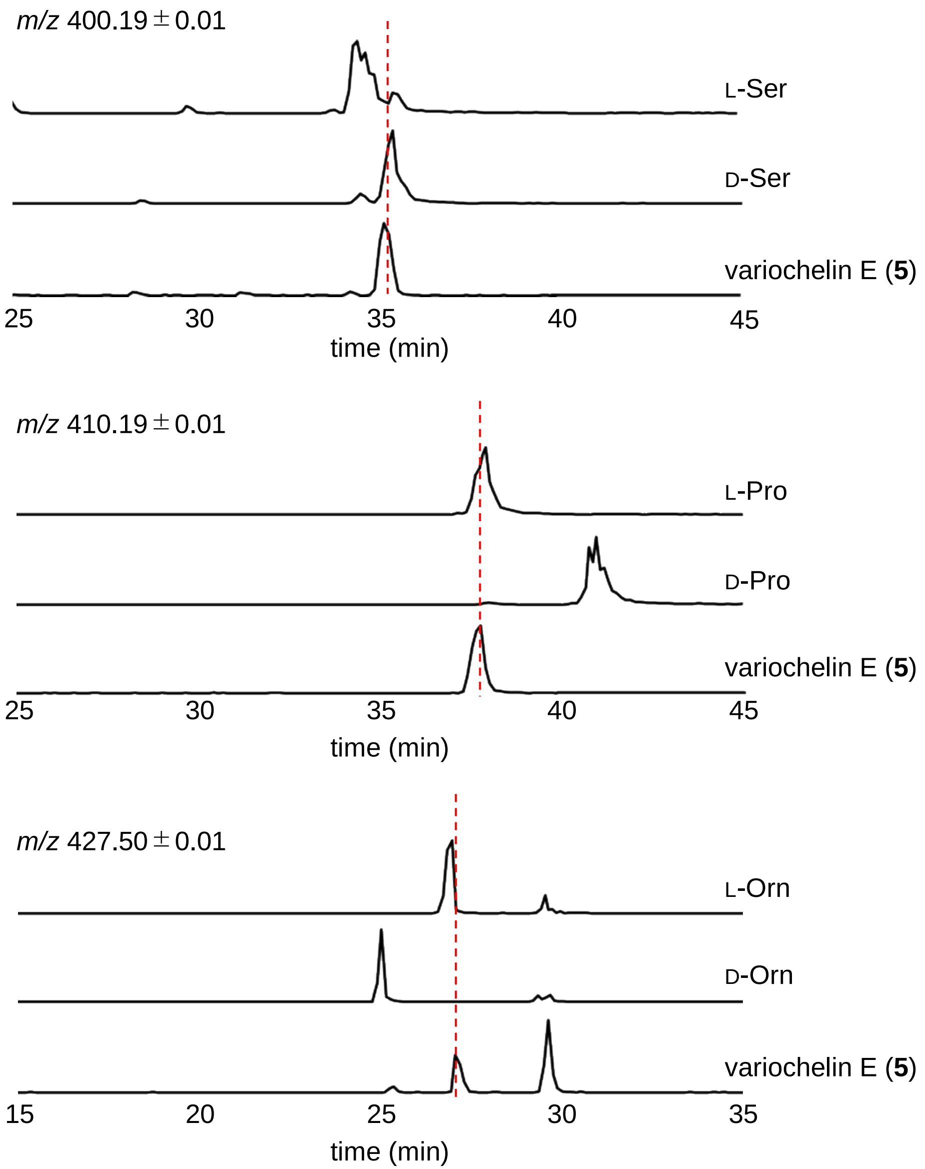

Figure S41. Extracted ion chromatograms from advanced Marfey's analyses of variochelin E (**5**).

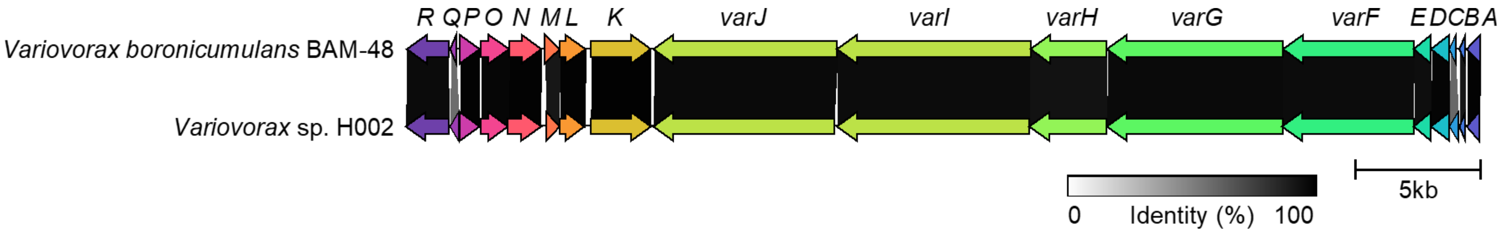

Figure S42. Variochelin BGCs comparison identified in *Variovorax boronicumulans* BAM-48 (up) and *Variovorax* sp. H002 (bottom) generated by clinker. Links between homologous genes are shown according to the percentage identity (see identity scale bar).

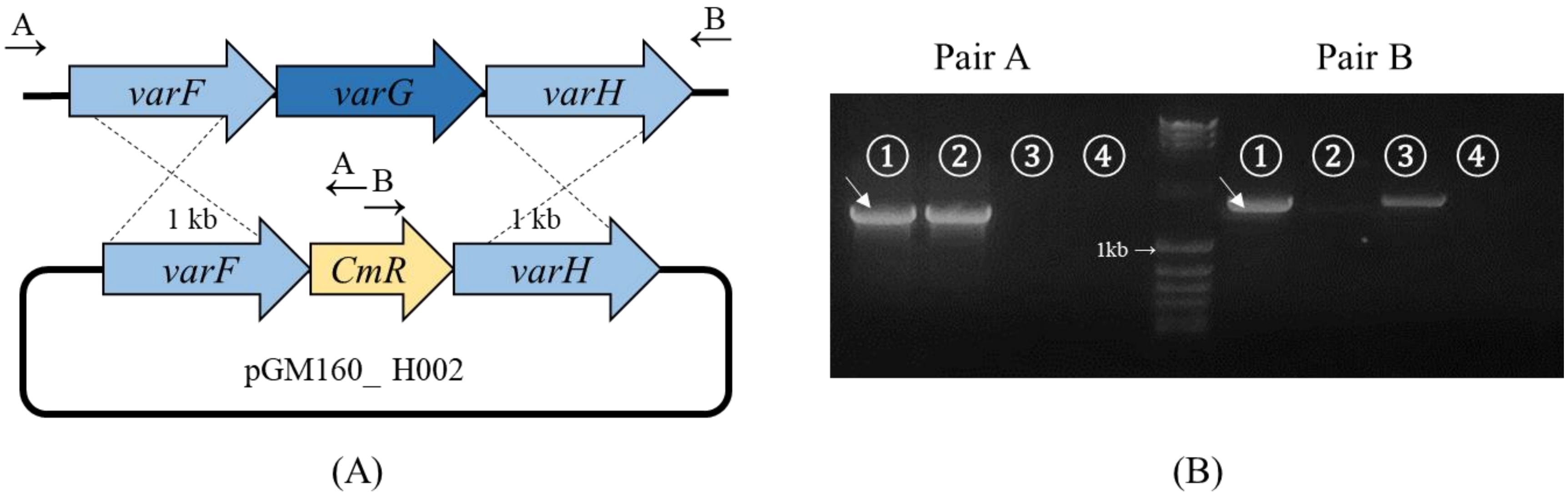

Figure S43. Generation of *Variovorax* sp. H002 *varG* null-mutant; (a) Illustration of *varG* knock-out using homolog recombination technology; dark blue-colored arrow is target gene to be replaced by yellow-colored chloramphenicol resistant gene (*cmR*) embedded in plasmid pGM160_H002; light blue-colored arrows are homologous flanking genes; small black arrows, pair A and pair B, represent primer sites employed for confirmation of successful recombination. (b) Confirmation of successful recombination in four selected mutants; PCR amplification followed by visualization on 1% Agarose S gel showed mutant number 1 generated positive band (white arrows) in both primers pair A and B, while other mutants only give one band of either primers (number 2 and 3) or no band detected (number 4). The mutant number 1 is used for further experiments.

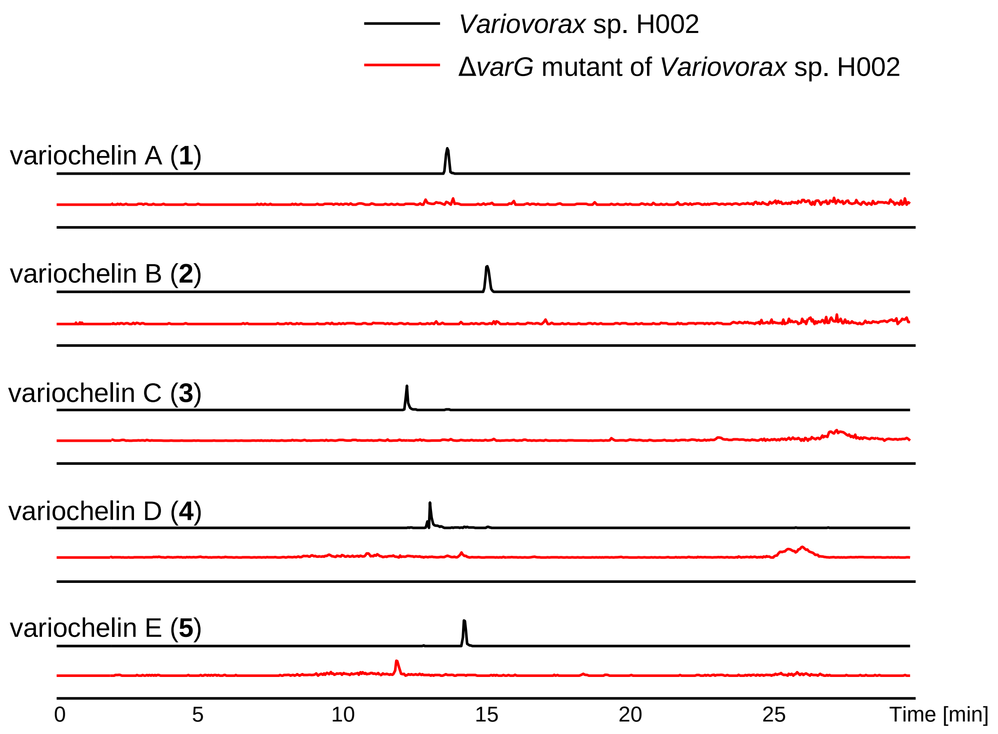

Figure S44. Extracted ion chromatogram showing deletion of *varG* abolishes variochelin A–E (**1**–**5**) production in *Variovorax* sp. H002. LC–MS-monitored production of variochelin A (*m/z* 1074.59 $[M + H]^+$), variochelin B (*m/z* 1102.62 $[M + H]^+$), variochelin C (*m/z* 1046.56 $[M + H]^+$), variochelin E (*m/z* 1072.58 $[M + H]^+$), and xenoamicin E (*m/z* 1100.61 $[M + H]^+$).

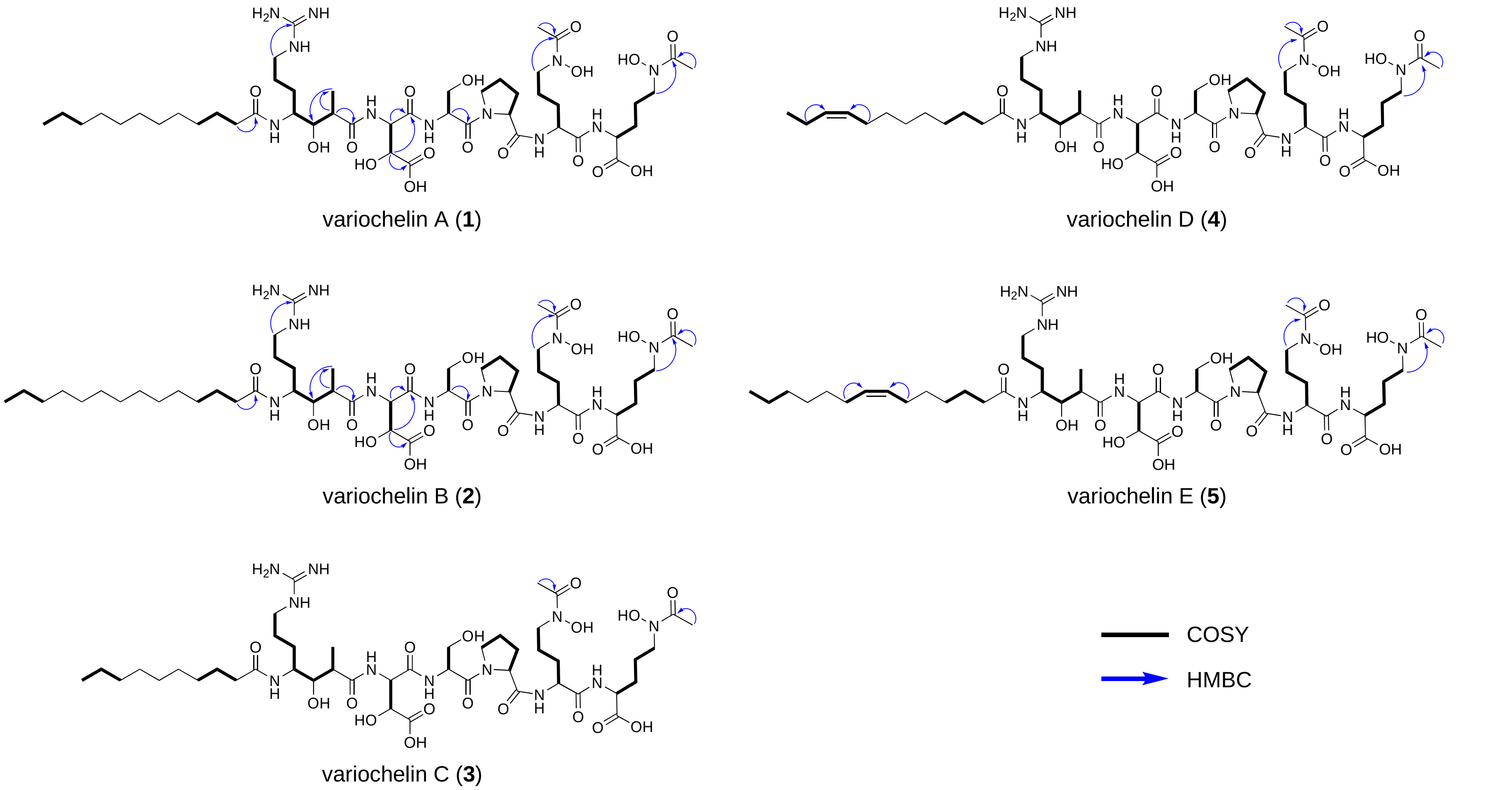

Figure S45. Selected COSY and HMBC correlations of variochelin A–E (**1**–**5**) in methanol-d_4.

Table S1. ^{1}H (500 MHz) and ^{13}C (125 MHz) NMR data of variochelins A–E (**1**–**5**) in methanol-d_4 (δ in ppm).

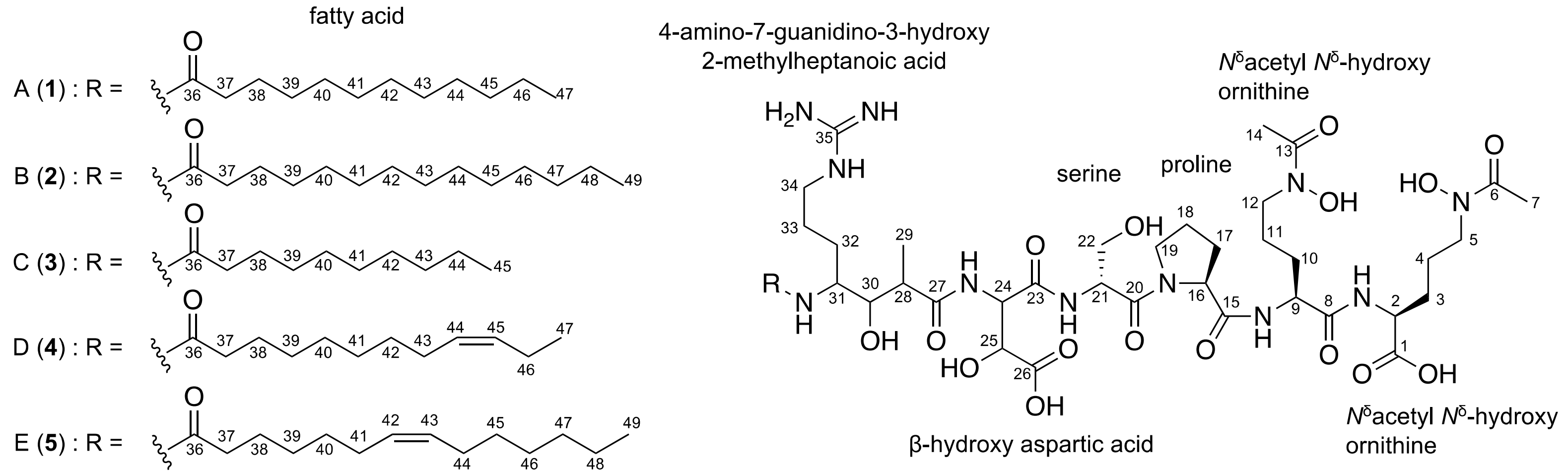

	Pos.	variochelin A (**1**)		variochelin B (**2**)		variochelin C (**3**)		variochelin D (**4**)		variochelin E (**5**)	
		δ_H, M (*J* in Hz)	δ_C	δ_H, M (*J* in Hz)	δ_C	δ_H, M (*J* in Hz)	δ_C	δ_H, M (*J* in Hz)	δ_C	δ_H, M (*J* in Hz)	δ_C
N$^{\delta}$acetyl *N*$^{\delta}$-hydroxy ornithine	1		n.r.		n.r.		n.r.		n.r.		n.r.
	2	4.33 (m)	55.2	4.33 (m)	55.1	4.33 (m)	55.3	4.32 (m)	55.3	4.34 (m)	55.1
	3	1.72-1.88 (br)	29.6	1.64-1.86 (br)	29.7	1.70-1.88 (br)	29.7	1.66-1.85 (br)	29.7	1.66-1.85 (br)	29.6
	4	1.59-171 (br)	25.9	1.59-171 (br)	25.9	1.59-171 (br)	25.9	1.62 (br)	25.9	1.62 (br)	25.7
	5	3.71 (br)	48.9*	3.73 (br)	48.9*	3.59 (br)	48.4*	3.59 (br)	48.4*	3.59 (br)	48.4*
	6		173.6		173.7		n.r.		173.6		173.6
	7	2.09 (s)	20.3	2.09 (s)	20.3	2.09 (s)	20.1	2.10 (s)	20.5	2.10 (s)	20.3
N$^{\delta}$acetyl *N*$^{\delta}$-hydroxy ornithine	8		n.r.		n.r.		n.r.		n.r.		n.r.

	9	4.39 (m)	54.4	4.39 (m)	54.5	4.36 (m)	55.1	4.36 (m)	55.1	4.38 (m)	55.1
	10	1.72-1.88 (br)	29.6	1.64-1.86 (br)	29.7	1.70-1.88 (br)	29.7	1.66-1.85 (br)	29.7	1.66-1.85 (br)	29.6
	11	1.53-1.81 (br)	25.9	1.52-1.71 (br)	25.9	1.65 (br)	25.9	1.65 (br)	25.9	1.65 (br)	25.7
	12	3.61 (br)	48.4*	3.61 (br)	48.4*	3.60 (br)	48.4*	3.60 (br)	48.4*	3.60 (br)	48.4*
	13		173.6		173.5		n.r.		173.6		173.6
	14	2.09 (s)	20.3	2.09 (s)	20.3	2.09 (s)	20.1		20.5	2.10 (s)	20.3
Proline (Pro)	15		n.r.		n.r.		n.r.		n.r.		n.r.
	16	4.46 (m)	62.3	4.46 (m)	62.3	4.47 (m)	62.0	4.46 (m)	62.1	4.46 (m)	62.1
	17	a. 2.22 (m)	30.1	a. 2.23 (m)	30.2	a. 2.22(m)	30.2	a. 2.20(m)	30.2	a. 2.20(m)	30.1
		b. 2.06 (m)		b. 2.07 (m)		b. 2.06 (m)		b. 2.04 (m)		b. 2.04 (m)	
	18	1.62 br	26.4	1.62 br	26.4	1.61 (br)	26.5	1.61 (br)	26.5	1.61 (br)	26.6
	19	3.61 (br)	48.7*	3.62 (br)	48.7*	3.60 (br)	48.4*	3.59 (br)	48.4*	3.59 (br)	48.4*
Serine (Ser)	20		171.2		171.2		n.r.		n.r.		n.r.
	21	4.61 (m)	55.3	4.62 (m)	55.3	4.64 (m)	55.0	4.60 (m)	55.0	4.62 (m)	55.2
	22	3.79 (m)	62.5	3.79 (m)	62.5	3.78 (m)	63.4	3.78 (m)	62.6	3.79 (m)	64.5
β-hydroxy aspartic acid (Hya)	23		171.8		171.9		n.r.		n.r.		n.r.
	24	4.89 (br)	57.4	4.89 (br)	57.4	4.90 (m)	57.2	4.91 (m)	57.5	4.91 (m)	57.3
	25	4.49 (br)	72.9	4.49 (br)	73.0	4.44 (m)	73.4	4.46 (m)	73.1	4.49 (m)	73.2
	26		177.3		177.3		n.r.		n.r.		n.r.
4-amino-7-guanidino -3-hydroxy	27		177.8		177.8		n.r.		n.r.		n.r.

2-methylheptanoic acid											
	28	2.69 (m)	44.3	2.67 (m)	44.3	2.70 (m)	43.9	2.69 (m)	44.1	2.69 (m)	44.5
	29	1.19 (d, 6.8)	14.4	1.20 (d, 6.8)	14.4	1.18 (d, 6.4)	n.r.	1.17 (br)	14.4	1.2 (br)	n.r.
	30	3.72 (m)	75.9	3.74 (m)	75.9	3.78 (m)	75.7	3.72 (m)	75.7	3.73 (m)	75.8
	31	3.92 (m)	52.6	3.92 (m)	52.6	3.90 (m)	52.6	3.89 (m)	52.6	3.91 (m)	52.5
	32	a. 1.45 (m)	29.8	a. 1.46 (m)	29.8	1.48-1.54 (br)	29.8	1.44-1.97 (br)	29.8	1.44-1.97 (br)	29.8
		b. 1.71 (m)		b. 1.71 (m)							
	33	1.61 (m)	24.1	1.61 (m)	24.1	1.54-1.70 (br)	24.2	1.58 (br)	24.2	1.58 (br)	24.2
	34	3.19 (m)	42.2	3.19 (m)	42.2	3.20 (m)	42.1	3.21 (m)	42.5	3.21 (m)	41.9
	35		158.7		158.7		n.r.		n.r.		n.r.
fatty acid	36		176.1		176.4		n.r.		n.r.		n.r.
	37	2.22 (m)	37.2	2.22 (m)	37.2	2.22 (m)	37.1	2.20 (m)	37.3	2.23 (m)	37.2
	38	1.62 (br)	23.7	1.61 (br)	23.7	1.62 (br)	24.1	1.61 (br)	24.1	1.61 (br)	24.3
	39	1.33 (br)	29.7-30.8	1.31 (br)	29.8-30.7	1.32 (br)	29.7-30.8	1.33 (br)	30.0-30.8	1.29 (br)	29.7-30.8
	40	1.33 (br)	29.7-30.8	1.31 (br)	29.8-30.7	1.32 (br)	29.7-30.8	1.33 (br)	30.0-30.8	1.29 (br)	29.7-30.8
	41	1.33 (br)	29.7-30.8	1.31 (br)	29.8-30.7	1.32 (br)	29.7-30.8	1.33 (br)	30.0-30.8	2.04 (br)	28.1
	42	1.33 (br)	29.7-30.8	1.31 (br)	29.8-30.7	1.32 (br)	29.7-30.8	1.33 (br)	30.0-30.8	5.35 (m, 4.6)	131.1
	43	1.33 (br)	29.7-30.8	1.31 (br)	29.8-30.7	1.32 (br)	29.7-30.8	2.04 (br)	28.1	5.35 (m, 4.6)	130.6
	44	1.33 (br)	29.7-30.8	1.31 (br)	29.8-30.7	1.32 (br)	29.7-30.8	5.35 (m, 4.6)	131.1	2.04 (br)	28.1

45	1.33 (br)	29.7-30.8	1.31 (br)	29.8-30.7	0.90 (t, 6.9)	14.1	5.35 (m, 4.6)	130.6	1.29 (br)	29.7-30.8
46	1.33 (br)	29.7-30.8	1.31 (br)	29.8-30.7			2.04 (br)	28.1	1.29 (br)	29.7-30.8
47	0.90 (t, 7.0)	14.4	1.31 (br)	29.8-30.7			0.90 (t, 6.9)	14.1	1.29 (br)	29.7-30.8
48			1.31 (br)	29.8-30.7					1.29 (br)	29.7-30.8
49			0.90 (t, 7.0)	14.4					0.90 (t, 6.9)	14.4

n.r. (not resolved due to broadening signals)

* assigned chemical shifts according to HSQC coupling

Table S2. List of primers, strains and plasmids used for generation of *varG* null-mutant.

Primers	Sequence (5' → 3'):
varG-up_fw	gccaaaagttggcccagggcTCAGAGGGTTCTTTCCGTGGG
varG -up_rv	tcttcctggcagataagcttCGTGAACGCCTACGGCCC
CmR_fw	CACCAATAACTGCCTTAAAAAAATTACG
CmR_rv	GCCCTGGGCCAACTTTTG
varG-down_fw	cggatgaatgggataagcttTGGGCGGCGCCTCGACGA
varG-down_rv	ttttaaggcagttattggtgATGACGACCATGACCGACCACTTCGAC
H002_checkA_fw	TCCAGCAGGCAGAGGTATTG
H002_checkA_rv	GCAAGATGTGGCGTGTTACG
H002_checkB_fw	CACCAGCTCACCGTCTTTCA
H002_checkB_ rv	ACAGCTCAGTGAACGGAACC
pRED_fw	ATCTGCCAGGAAGATACTTAAC
pRED_rv	ATCCCATTCATCCGCTTATTATC
**Strains and plasmids**	**Genotype or description**
E. coli DH5a	F^- φ80*lacZ*ΔM15 Δ(*lacZYA-argF*) U169 *endA1 recA1 hsdR17* (rk^-, mk^+) *supE44* thi^{-1} *gyrA996 relA1 phoA*
E. coli S17-1 λ*pir*	λ *pir hsdR pro thi*; chromosomal integrated RP4-2 Tc::Mu m::Tn7
Variovorax sp. H002	Wild type
pRED	Cloning vector; Cm^r
pGM160	Conjugation vector; Amp^r, Tsr^r, Neo^r, Kan^r
pRED_H002	H002 Knock-out cassette on pRED
pGM160_ H002	H002 Knock-out cassette on pGM160

Table S3. Annotation of *var* biosynthetic gene cluster in *Variovorax* sp. H002.

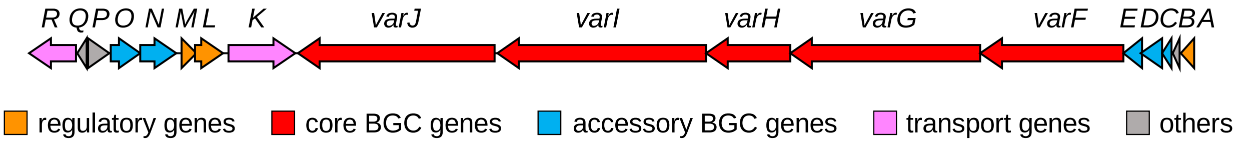

ORF	Nucl.	AA	Homolog	Cov./Id.	Accession
A	534	177	RNA polymerase sigma factor	99/83.5	WP_092757814.1
B	246	81	FecR/PupR family sigma factor regulator	91/59.5	WP_166300416.1
C	249	82	MbtH family	98/82.7	WP_211958485.1
D	747	248	Thioesterase	92/70.4	SDO97538.1
E	702	233	4'-phosphopantetheinyl transferase	95/57.7	WP_272548812.1
F	5265	1754	NRPS (FAAL-A-PCP)	-	-
G	7056	2351	PKS (KS-AT-KR-ACP-C)	-	-
H	3105	1034	NRPS (C-A-PCP)	-	-
I	7764	2587	NRPS (C-A-PCP-E-C-A-PCP)	-	-
J	7377	2458	NRPS (C-A-PCP-C-A-PCP-TE)	-	-
K	2448	815	TonB-dependent siderophore receptor	96/60.3	WP_198971676.1
L	1029	342	Iron dicitrate transport regulator FecR	97/66.3	WP_121454243.1
M	579	192	RNA polymerase sigma factor	99/78.4	WP_092759873.1
M	1320	439	L-Ornithine *N*(5)-oxygenase	99/71.5	ODT73388.1
O	1107	368	Acetyltransferase	94/58.0	WP_090907594.1
P	786	261	Siderophore-iron reductase	99/89.6	RIX80240.1
Q	234	77	Hypothetical protein	77/75.4	WP_092759869.1
R	1680	559	Cyclic peptide export ABC transporter	98/74.73	WP_211956633.1
